# Elevated *wzc* mutation frequency promotes carbapenem resistance evolution in hypervirulent *Klebsiella pneumoniae* ST23

**DOI:** 10.1080/22221751.2026.2726043

**Published:** 2026-08-27

**Authors:** Tao Chen, Xueting Wang, Luying Xiong, Zhuoqi Lou, Ping Lu, Zhien He, Qi Ge, Haoxing Wu, Qixia Luo, Ping Shen, Yunbo Chen, Hui Ding, Yan Geng, Kunpeng Liang, Baorui Yang, Tingting Xiao, Wei Yu, Yonghong Xiao

**Affiliations:** a State Key Laboratory for Diagnosis and Treatment of Infectious Diseases, National Clinical Research Center for Infectious Diseases, China-Singapore Belt and Road Joint Laboratory on Infection Research and Drug Development, National Medical Center for Infectious Diseases, Collaborative Innovation Center for Diagnosis and Treatment of Infectious Diseases, The First Affiliated Hospital, Zhejiang University School of Medicine, Hangzhou, People’s Republic of China; b Department of Clinical Laboratory, Zhejiang Cancer Hospital, Hangzhou, People’s Republic of China; c Department of Structure and Morphology, Jinan Microecological Biomedicine Shandong Laboratory, Jinan, People’s Republic of China; d Department of Clinical Laboratory, Lishui Municipal Central Hospital, Lishui, People’s Republic of China; e Department of Clinical Laboratory, The Second Affiliated Hospital of Xi'an Jiaotong University, Xi'an, People’s Republic of China; f Department of Clinical Laboratory, The Second Affiliated Hospital of Bengbu Medical University, Bengbu, People’s Republic of China; g Department of Clinical Laboratory, The Second People's Hospital of Yunnan Province, Kunming, People’s Republic of China; h Peking Union Medical College & Institute of Pathogen Biology, Chinese Academy of Medical Sciences & Research Units of Infectious Disease and Microecology, Chinese Academy of Medical Sciences, Beijing, People’s Republic of China

**Keywords:** *Klebsiella pneumoniae*, ST23, hypervirulent, carbapenem resistance, *wzc* mutation, evolution

## Abstract

The worldwide expansion of carbapenemase-producing hypervirulent *K. pneumoniae* (CP-hvKP) raises serious concerns about potentially untreatable invasive infections. ST23, an archetypal hvKP clone, has been highlighted due to the rising prevalence of carbapenem resistance largely driven by horizontal plasmid transfer. However, the factors facilitating carbapenemase acquisition in this lineage remained unclear. We characterized 1159 ST23 isolates across 49 countries, including nine clinical carbapenemase-producing strains from China’s national bloodstream infection surveillance network. Global phylogenetic analysis revealed two distinct sublineages, namely the globally distributed, hypervirulent ST23-I and the geographically restricted, multidrug-resistant ST23-II. Temporal analysis showed that ST23-I concurrently accumulated virulence and antimicrobial resistance traits. Among ST23-I isolates, carbapenemase plasmids exhibited notable genetic diversity and clear geographic segregation, with IncL-*bla*_OXA-48_ dominated in Europe and IncFII-*bla*_KPC-2_ in Asia. Conjugation assays revealed that IncFII_K34_-*bla*_KPC-2_ and IncL-*bla*_OXA-48_ plasmids transferred more efficiently than IncX3-*bla*_NDM-1_ and IncFII_K2_-*bla*_NDM-1_ plasmids, contributing to their high prevalence. Non-synonymous mutations in the capsular polysaccharide synthesis locus, particularly in *wzc*, were accumulated in ST23-KL1 CP-hvKP. Isogenic mutants carrying two identified *wzc* mutations (either *wzc*^2042A^ ^>^ ^G^ or *wzc*^1738T^ ^>^ ^A^) exhibited partially reduced capsule production and enhanced conjugation efficiency of carbapenemase plasmids, confirming the functional impact of these mutations. Collectively, these findings demonstrate that an elevated *wzc* mutation frequency represents an adaptive evolutionary pathway that facilitates carbapenemase acquisition in hvKP ST23. The interplay between capsule-associated chromosomal mutations and plasmid-mediated horizontal gene transfer may shape the evolutionary adaptation of resistance in this clinically important pathogen.

## Introduction

*Klebsiella pneumoniae* is a major cause of infections, including pneumonia, bacteremia, and liver abscesses [1,2]. *K. pneumoniae* can be bifurcated into two groups based on phenotypic and genotypic differences: the classic *K. pneumoniae* (cKP) and the hypervirulent *K. pneumoniae* (hvKP), each posing unique challenges to healthcare providers [3]. hvKP strains are capable of causing severe community-acquired infections in healthy hosts and increasingly reported in recent years. Unlike cKP, hvKP strains generally display a more susceptible antimicrobial profile [4]. However, the emergence of *K. pneumoniae* strains that concurrently harbor hypervirulence traits and carbapenemase-producing capabilities (CP-hvKP) raises serious concerns about potentially untreatable infections. The dissemination of CP-hvKP strains within healthcare settings poses a significant threat to vulnerable patient populations and may substantially elevate morbidity and mortality rates. Furthermore, the development of effective vaccines against *K. pneumoniae* remains technically challenging. The extensive genetic diversity of capsular polysaccharides (CPS) and O antigen, which serve as the primary targets for serotype-specific immunity, complicates the design of broad-spectrum vaccines [5,6]. Additionally, the high antigenic variability and the potential for capsule switching pose significant hurdles to achieving durable, cross-protective immunity [7,8].

Among hvKP lineages, sequence type (ST) 23 with the KL1 capsular serotype is the archetypal hvKP clone, defined by its carriage of the pK2044-like virulence plasmid and yersiniabactin-encoding integrative and conjugative element ICE*Kp* [9–11]. Historically, ST23 hvKP strains were predominantly isolated in Asia, primarily associated with community-onset infections, and rarely exhibited antimicrobial resistance [12]. However, recent reports indicated an expanding geographic distribution, a shift toward healthcare-associated infections, and a growing prevalence of carbapenem resistance [13,14]. Notably, multiple clusters of ST23 CP-hvKP isolates have been linked to sustained transmission within healthcare facilities across several European countries, signaling a concerning trend of localized outbreaks with the potential for international dissemination [14–16]. The global emergence and dissemination of these high-risk ST23 CP-hvKP clones pose a formidable challenge to both clinical management and public health, an issue explicitly flagged by the World Health Organization as an emerging pathogen threat [17]. Recent studies have increasingly described *K. pneumoniae* isolates that successfully combine hypervirulence and multidrug resistance [18]. Given the potential for these clones to traverse between community and hospital, implementing surveillance and intervention strategies within a One Health framework is critical to curb the spread of such high-risk lineages.

We previously characterized the spatiotemporal distribution and molecular epidemiology of ST23 carbapenem-resistant hvKP (CR-hvKP) in China [19]. Globally, however, most reports of CP-hvKP ST23 remain sporadic, often limited to single-center studies focusing on individual clinical isolates or case series [16,20–22]. Although a few studies have examined the phylogeny and evolutionary history of the ST23-KL1 sublineage [12,23], critical gaps persist in our understanding of the adaptive mechanisms underlying the emergence of CP-hvKP. In addition, some researchers have suggested that hvKP strains carrying carbapenemase plasmids may struggle to maintain both high-level virulence and carbapenem resistance simultaneously, a phenomenon referred to as the virulence–resistance trade-off [24,25]. Nevertheless, evidence supporting this hypothesis, derived from large-scale genomic analyses coupled with functional validation, remains lacking.

In this study, we performed a global comparative analysis incorporating an international collection of 1159 *K. pneumoniae* ST23 isolates from 49 countries, with sampling dates ranging from 1980 to 2023. By integrating large-scale genomic epidemiology with experimental validation, we demonstrated that an elevated frequency of *wzc* mutations represented an adaptive mechanism that facilitated the acquisition of epidemic plasmids, promoting the emergence of CP-hvKP ST23-KL1.

## Materials and methods

### Identification of ST23 CRKP isolates in China

From January 2014 to December 2023, CRKP isolates were collected from a national bloodstream infection surveillance network in China. Carbapenem resistance was defined as a minimum inhibitory concentration (MIC) of imipenem or meropenem ≥4 μg/mL, or an MIC of ertapenem ≥2 μg/mL. From this collection, we identified ten ST23 CRKP isolates for this study.

### Antimicrobial susceptibility testing

The agar dilution method using Mueller-Hinton (MH) agar was employed to determine the MICs of piperacillin/tazobactam, cefoperazone/sulbactam, ceftriaxone, ceftazidime, cefuroxime, cefepime, moxalactam, aztreonam, imipenem, meropenem, ertapenem, ceftazidime/avibactam, ciprofloxacin, levofloxacin, amikacin, gentamicin, sulfamethoxazole/trimethoprim, and fosfomycin. For polymyxin B and tigecycline, the broth microdilution method utilizing Cation-Adjusted Mueller-Hinton broth (CAMHB) was applied. Susceptibility results were interpreted according to the Clinical and Laboratory Standards Institute (CLSI) 2023 guidelines, with the exception of tigecycline, which was evaluated based on the European Committee on Antimicrobial Susceptibility Testing (EUCAST) v13.0 breakpoints.

### Whole genome sequencing and bioinformatic analysis

Genomic DNA from CRKP isolates was extracted using Gentra Puregene Yeast/Bact. Kit (Qiagen). The genomes were sequenced using the Illumina HiSeq X-Ten platform with a 2 × 150 bp paired-end read. The quality control of raw sequenced reads was conducted using FastQC v0.11.5. Raw reads were trimmed using Trimmomatic v0.33 and assembled de novo using SPAdes v3.10.0. The STs were assigned using Kleborate v2.3.2 [26], and the K/O-type was determined using Kaptive v2.0 from the de novo assembly. We performed long-read sequencing on ten ST23 CRKP isolates using a Nanopore PromethION platform following a 10 kb library protocol. A hybrid assembly was generated by using Unicycler v0.4.8 with short and long reads. The quality of the assembled genomes was assessed using QUAST v5.2.0. Annotation was performed using Prokka **v**1.13 [27,28].

Virulence genes (VGs) and plasmid incompatibility (Inc) types were identified using ABRicate v1.0.1, with annotations drawn from the VFDB and PlasmidFinder databases, respectively. Analyses were performed under stringent thresholds of ≥95% nucleotide identity and ≥80% query coverage. For plasmids assigned to the IncF group, plasmid multi-locus sequence typing (pMLST) was conducted to refine incompatibility classification [29]. Antibiotic resistance genes (ARGs) and chromosomal resistance-associated mutations were detected using AMRFinderPlus v3.12.8 [30]. To characterize plasmids in ST23 strains, including their mobility potential, we employed MOB-typer from the MOB-suite v1.4.9 [31].

The phylogenetic analysis incorporated 10 ST23 CRKP genomes from this study, 105 carbapenem-susceptible isolates from liver abscess patients previously sequenced by our group, and 1044 GenBank genomes. The hypervirulent serotype K1 reference strain NTUH-K2044 was used as the genomic reference. All isolates were aligned to NTUH-K2044 using Snippy v4.6.0. Recombinant regions were identified and removed with Gubbins v2.4.1 to generate high-quality multiple sequence alignments (MSAs) and a core-genome SNP matrix. The population structure of the global ST23 collection was inferred using the rhierBAPS algorithm [32]. A maximum-likelihood phylogenetic tree was inferred using IQ-TREE2 under the GTR + GAMMA substitution model and visualized with iTOL v5 [33]. The capsular polysaccharide synthesis (*cps*) loci of all ST23-KL1 isolates were aligned against the reference strain NTUH-K2044 using Snippy v4.6.0 to identify isolate-specific mutations. The SNP frequency per site between CP-hvKP and non-CP-hvKP isolates was analyzed in R v4.3.2.

To minimize computational burden, a representative subset of 255 ST23-KL1 genomes from 12 major countries was selected for BEAST analysis. From each country, at least ten strains were randomly sampled; additionally, six strains were randomly chosen per calendar year of isolation. These sequences underwent the same SNP extraction pipeline described above. The resulting recombination-filtered multiple sequence alignments (MSAs) were visualized using BEAST v1.10.4 [34]. The most appropriate demographic model for this study was defined as the GTR nucleotide substitution model with four gamma categories, combined with an uncorrelated relaxed clock model and a coalescent tree prior with constant population size. Temporal signal in molecular evolution was assessed with TempEst v1.5.3, and MCMC convergence was evaluated in Tracer v1.7.2, where all parameters achieved effective sample sizes (ESS) exceeding 200. Finally, phylogeographic reconstruction was performed using SpreaD3 [35].

### Capsule extraction and quantification assay

Overnight cultures grown in LB broth were harvested, and a total bacterial biomass equivalent to an OD_600_ of 10 was used for capsular polysaccharide (CPS) extraction. CPS was extracted using a standard protocol by Bacterial Extracellular Polysaccharide Extraction Kit (BBproExtra). CPS was then analyzed by Alcian blue staining and the carbazole-sulfuric acid method. For Alcian blue staining, 20 μL of the CPS sample was mixed with bromophenol blue dye and subjected to SDS-PAGE at 100 V. The gel was washed four times with ddH_2_O and then stained with Alcian blue overnight for visualization. For the carbazole-sulfuric acid quantification, 20 μL of extracted CPS was mixed with 120 μL of 12.5 mM sodium tetraborate in concentrated sulfuric acid, incubated at 100 °C for 5 min, and then rapidly cooled on ice for 5 min. Subsequently, 20 μL of 0.15% 3-phenylphenol in 0.5% NaOH was added, and the mixture was incubated overnight at 4 °C. The absorbance at 520 nm was measured with a spectrophotometer.

### Transferability and stability of carbapenemase-encoding plasmids

Conjugative transfer of carbapenemase-encoding plasmids was assessed using filter mating assays. For conjugation from ST23 CP-hvKP donors, rifampicin-resistant *E. coli* EC600 served as the recipient strain. Transconjugants were selected on LB agar containing meropenem (0.5 μg/mL) and rifampicin (100 μg/mL). In reciprocal experiments, plasmid-carrying EC600 donors were mated with dipotassium tellurite-resistant hvKP NTUH-K2044, and transconjugants were selected on LB agar supplemented with meropenem (0.1 μg/mL) and dipotassium tellurite (3 μg/mL). Successful plasmid transfer was confirmed by PCR amplification of plasmid replicon types and carbapenemase genes (*bla*_KPC-2_, *bla*_NDM-1_, or *bla*_OXA-48_), followed by Sanger sequencing. Conjugation frequency was calculated as the ratio of transconjugant to recipient CFU.

Plasmid stability was evaluated through a 10-day serial passage experiment in the absence of antibiotics. Briefly, three independent colonies of each strain were inoculated into antibiotic-free M9 minimal medium and cultured at 37 °C with shaking (200 rpm). Each day, 1 μL of overnight culture was transferred into fresh medium at a 1:1000 dilution. Every five days, cultures were serially diluted in PBS and plated onto MH agar without antibiotics. Fifty randomly selected colonies from each lineage were then replica-plated onto MH agar containing meropenem (0.1 μg/mL) to assess plasmid retention. Plasmid stability was expressed as the proportion of meropenem-resistant colonies relative to the total viable count on antibiotic-free plates. The presence of carbapenemase genes, serving as plasmid markers, was verified by PCR.

### Construction of mutant and complementation strains

Mutants were generated using the λ Red recombinase-mediated recombineering system, as previously reported [36]. The plasmid pACBSR, which expresses the λ Red genes under an arabinose-inducible promoter, was used for recombination. Approximately 500-bp DNA fragments flanking the *wzc* locus were amplified from NTUH-K2044 genomic DNA using primers listed in Table S1. An apramycin resistance cassette, flanked by FRT sites for subsequent removal, was amplified from plasmid pCAP03-*acc(3)IV* and inserted between the homology arms to replace the target gene. After selection, the apramycin cassette was excised from the chromosome by expressing Flp recombinase from the helper plasmid pFLP. The precise deletion of the target gene and the complete removal of the resistance cassette were confirmed via Sanger sequencing of the flanking regions.

For genetic complementation, the wild-type *wzc* allele was PCR-amplified from NTUH-K2044 genomic DNA, while the point-mutated variant was generated by site-directed mutagenesis using mutagenic primers (Table S1). Both fragments were then cloned into the pBBR1-*hygR* vector via homologous recombination. The resulting plasmids were introduced via electroporation into the Δ*wzc* mutant strain of NTUH-K2044. Finally, Sanger sequencing was performed to verify the successful integration of both the wild-type and point-mutated *wzc* alleles.

### Murine infection model

A murine intraperitoneal infection model was employed to evaluate the virulence potential of each strain, following a previously established protocol [37]. Five-week-old female BALB/c mice (n = 5 per group) were inoculated intraperitoneally with 100 μL of PBS containing 10^5^ CFU of the tested strains. As a control, a separate group of mice received an intraperitoneal injection of 100 μL of sterile PBS. Following infection, the mice were monitored every 12 h for physical condition and survival. The survival rate was recorded over a five-day observation period.

### In vitro competition assays

*In vitro* competition index (CI) values were calculated to evaluate relative fitness as previously described [38]. Two sets of competition assays were performed. First, to assess *wzc* mutant fitness, exponential-phase cultures of NTUH-K2044 *wzc* mutant-complemented strains (apramycin-resistant) and wild-type *wzc*-complemented strains were mixed at a 1:1 ratio (2.5 × 10⁷ CFU each) in 4 mL of LB broth and incubated at 37°C with shaking for 24 h. Second, to evaluate the fitness costs of plasmid acquisition, wild-type NTUH-K2044 was competed against isogenic transconjugants carrying various carbapenemase-encoding plasmids under identical conditions. For both assays, aliquots (100 μL) were sampled at 0 and 24 h, and CFUs were enumerated via serial dilution plating on LB agar with or without selective antibiotics (apramycin at 20 μg/mL for *wzc* mutants; meropenem at 0.2 μg/mL for plasmid-bearing strains).

### Electrotransformation efficiency assay

Electrotransformation efficiency was determined as previously described [39]. Briefly, bacterial cells in the exponential phase were washed twice with ice-cold 10% glycerol, resuspended in 1 mL of 10% glycerol, and adjusted to a concentration of approximately 2 × 10^10^ cells/mL. A 500 ng (1 μL) aliquot of carbapenemase-encoding plasmid DNA was added to 50 μL of competent cells. Electroporation was performed at 1300 V/cm, 25 μF, and 100 Ω. Immediately after electroporation, 600 μL of precooled SOC medium was added, and the suspension was incubated at 37°C for 30 min before being plated on LB agar supplemented with 0.2 μg/mL meropenem. Colonies were counted after 16 h of incubation at 37°C.

### Statistical analysis

All statistical analyses were conducted using SPSS v22.0. Student’s t-test was performed for continuous variables, and Chi-square or Fisher’s exact test was conducted for categorical data as applicable. Multiple comparisons were corrected using the Bonferroni procedure. A *P*-value < 0.05 was considered statistically significant. The association between isolation year and gene counts was evaluated using nonparametric regression.

## Results

### Geographic, microbiological, and genomic characteristics of ST23 CRKP in China

Ten ST23 CRKP isolates were recovered from ten distinct patients treated at eight different hospitals across five provinces in China as part of the national bloodstream infection surveillance network, with four isolates from Zhejiang, two each from Shaanxi and Anhui, and one each from Yunnan and Jiangxi, reflecting a broad geographic distribution (Figure 1(A)).

**Figure 1.**
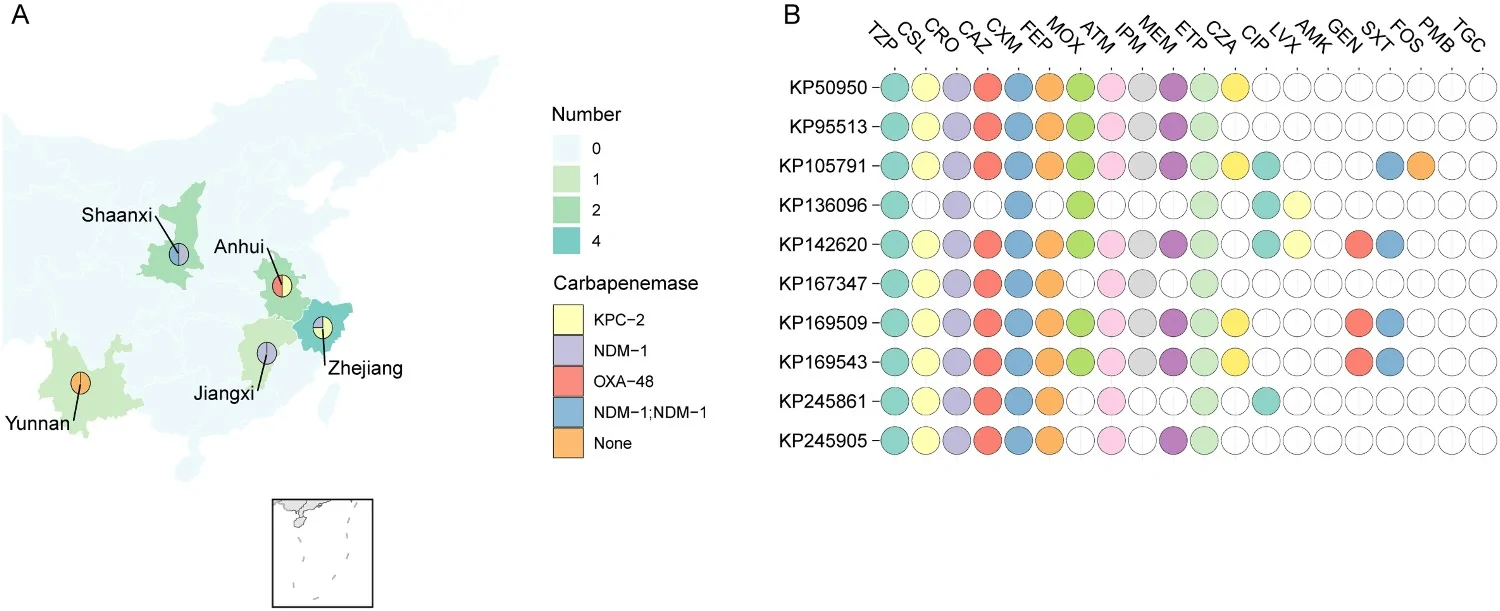
Geographic distribution and microbiological characteristics of ST23 CRKP bloodstream infection (BSI) isolates in China. (A) Geographic distribution of ten ST23 CRKP BSI strains within China. (B) Antimicrobial resistance profile of the ten ST23 CRKP isolates against commonly used antibiotics. Colored circles indicate resistance or intermediate susceptibility; open circles indicate susceptibility. TZP, piperacillin/tazobactam; CSL, cefoperazone/sulbactam; CRO, ceftriaxone; CAZ, ceftazidime; CXM, cefuroxime; FEP, cefepime; MOX, moxalactam; ATM, aztreonam; IPM, imipenem; MEM, meropenem; ETP, ertapenem; CZA, ceftazidime/avibactam; CIP, ciprofloxacin; LVX, levofloxacin; AMK, amikacin; GEN, gentamicin; SXT, sulfamethoxazole/trimethoprim; FOS, fosfomycin; PMB, polymyxin B; TGC, tigecycline.

The results of antimicrobial susceptibility testing revealed that the ten ST23 CRKP isolates displayed diverse antimicrobial resistance profiles. All ten ST23 isolates were susceptible to polymyxin B, tigecycline, and amikacin (Figure 1(B)). Notably, all isolates were resistant to ertapenem (MIC values ranging from 2 to 32 μg/mL), but four strains (KP136096, KP167347, KP245861, and KP245905) remained susceptible to imipenem and/or meropenem (MIC values ranging from 0.25 to 2 μg/mL) (Table S2). Furthermore, four strains (KP50950, KP105791, KP169509, and KP169543) exhibited resistance to ceftazidime/avibactam, and three strains (KP142620, KP169509, and KP169543) were resistant to gentamicin.

The ten ST23 isolates underwent MinION (Oxford Nanopore) sequencing to generate long-read data for genomic characterization. Hybrid assemblies were performed by integrating Illumina short reads with Nanopore long reads. All ten isolates belonged to serotype K1/O1. Virulence profiling using Kleborate showed that nine isolates carried the yersiniabactin (*ybt*), colibactin (*clb*), and aerobactin (*iuc*) gene clusters, yielding a virulence score of 5. The remaining isolate, KP95513, lacked *iuc* and thus had a reduced score of 2, harboring only *ybt* and *clb*. To investigate the carbapenem resistance mechanisms of the ten ST23 CRKP isolates, we analyzed their acquired ARGs, which ranged from 4 to 16 per isolate (Table S3). Among the ten isolates, carbapenemase genes were detected in nine: four harbored *bla*_NDM-1_, four carried *bla*_KPC-2_, and one possessed *bla*_OXA-48_, while the remaining isolate (KP136096) lacked any known carbapenemase gene, suggesting that resistance in this isolate may be mediated by other mechanisms, such as alterations in membrane permeability (Table S3). BLAST analysis of the KP136096 genome revealed three mutations in OmpK36: p.Asp49Ser (GAC→AGC), p.Leu59Val (CTT→GTA), and p.Ser109fs (TCTTTCGACTAC→T----------C). Additionally, three missense mutations were identified in OmpK37: p.Ile70Met (ATT→ATG), p.Ile128Met (ATT→ATG), and p.Thr261Ala (ACC→GCC) (Figure S1). Notably, the p.Ile70Met and p.Ile128Met substitutions in OmpK37 have been previously implicated in carbapenem resistance [40], indicating that these porin structural alterations could potentially contribute to the resistance phenotype observed in KP136096.

### Global population structure and geographic distribution of ST23K. pneumoniae

To explore the population structure of global ST23*K. pneumoniae* isolates, we compiled a comprehensive dataset of 1159 genomes, comprising ten CRKP and 105 carbapenem-susceptible isolates previously sequenced by our group [41] along with 1044 publicly available GenBank genomes (Table S4). These 1159 ST23 isolates were collected from 49 countries between 1980 and 2023, with the majority originating from China (n = 456), followed by Ireland (n = 95), Japan (n = 90), and others (Figure 2(A)). Capsular locus typing revealed that KL1 was overwhelmingly dominant (94.74%, 1098/1159), followed by KL57 (4.06%, 47/1159). Geographically, KL57 strains were significantly enriched in Europe compared to other continents (15.85%, 45/284 vs. 0.12%, 1/847; Fisher’s exact test, *P* < 0.001) (Figure S2A). Phylogenetic analysis based on cgSNPs resolved the global ST23 population into two genetically distinct clades: a major, globally distributed hypervirulent clade (ST23-I) dominated by ST23-KL1 (n = 1111) and a smaller clade (ST23-II) primarily composed of ST23-KL57 (n = 48) (Figure S2B). The inter-clade cgSNP distances between ST23-I and ST23-II ranged from 11,172 to 11,953, whereas intra-clade pairwise cgSNP differences were markedly lower (0–844 within the ST23-I clade and 2–91 within the ST23-II clade), indicating that ST23-I and ST23-II represent two evolutionarily divergent lineages despite sharing an identical seven-locus MLST profile.

**Figure 2.**
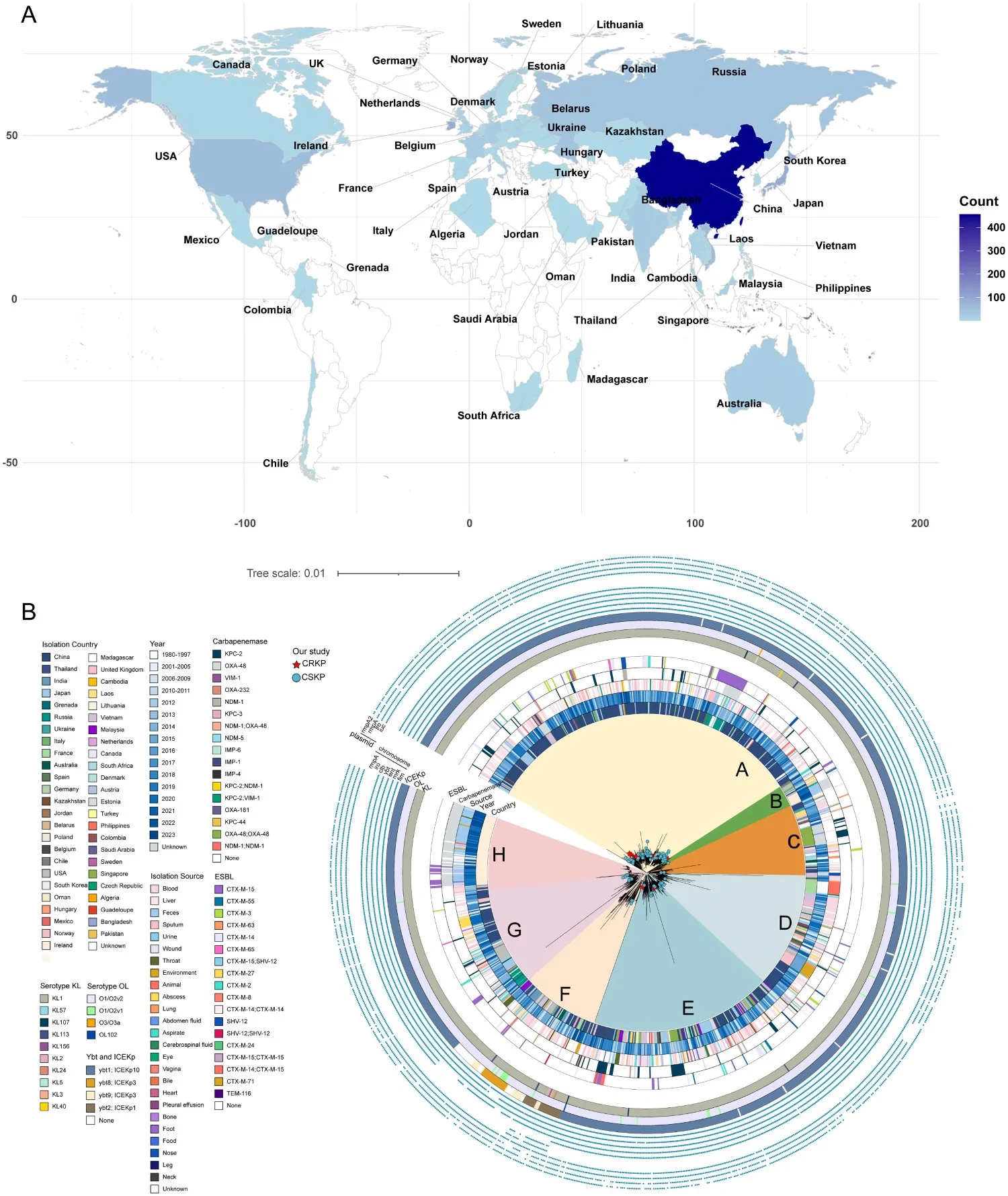
Phylogenetic analysis of global ST23*K. pneumoniae* isolates. (A) Geographical distribution of 1159 global ST23*K. pneumoniae* isolates. (B) Maximum-likelihood phylogenetic tree of 1111 isolates within the ST23-KL1 lineage. Metadata including country, year, source, carbapenemase, extended-spectrum beta-lactamase (ESBL), KL type, OL type, ICE*Kp* element, and the presence of virulence gene clusters are displayed as concentric rings from the inner to the outer circle.

We therefore reconstructed separate phylogenies for the ST23-I and ST23-II lineages. Within the ST23-I lineage, isolates were predominantly from Asia (66.79%, 742/1111) and overwhelmingly associated with the KL1-O1/O2v2 serotype (97.30%, 1081/1111), interspersed with minor serotypes such as KL2-O1/O2v2, KL5-O3/O3a, KL107-OL102, and KL113-O1/O2v1, likely resulting from frequent recombination events in the capsular polysaccharide synthesis locus (Figure 2(B)). Eight major clades (A–H) were identified, each exhibiting distinct geographic patterns: Clade A was predominant in China, Clades B and H in Europe, and Clades C–G primarily in Asia. Our ten ST23 CRKP isolates were distributed across Clade A (n = 8) and Clade E (n = 2), while the 105 CSKP isolates were scattered throughout the tree (absent only from Clades B and H), suggesting independent evolutionary trajectories and extensive dissemination (Figure 2(B)). Notably, strains in Clade F displayed remarkable diversity in ICE*Kp* and *ybt* combinations: 28.09% (25/89) carried ICE*Kp1*-*ybt2*, 22.47% (20/89) ICE*Kp3*-*ybt8*, 19.10% (17/89) ICE*Kp3*-*ybt9*, and 30.34% (27/89) lacked ICE*Kp* entirely. In contrast, isolates in all other clades almost universally harbored ICE*Kp10* and *ybt1* (99.02%, 1012/1022; Fisher’s exact test, all *P* < 0.001). Furthermore, all Clade F strains were *clb*-negative (0/89), whereas *clb* was present in 99.71% (1019/1022) of strains in other clades (*P* < 0.001), collectively suggesting that ICE*Kp* in Clade F has undergone more extensive evolutionary diversification. Intriguingly, Clade H exhibited a striking enrichment of carbapenemase genes: 81.93% (68/83) of its isolates carried at least one carbapenemase, significantly higher than the 15.18% (156/1028) observed in other clades (*P* < 0.001), highlighting its association with high-level antimicrobial resistance. Further analysis revealed that Clade H was predominantly distributed in Ireland (92.77%, 77/83) and exhibited an increase in prevalence since 2019. Notably, this clade primarily carried IncL-*bla*_OXA-48_ plasmids, which accounted for 98.53% of the carbapenemase-positive isolates in this clade.

In contrast, the ST23-II lineage was highly homogeneous, with 97.92% (47/48) of isolates carrying the KL57-O1/O2v2 capsular type and consistently associated with ICE*Kp3*. This lineage showed strong geographic restriction, with 95.83% (46/48) of isolates originating from Europe, particularly Germany (n = 16), Poland (n = 11), and Russia (n = 11) between 2014 and 2022. Phylogenetic reconstruction of this lineage resolved five major clades (a–e) with distinct regional and resistance profiles: Clade a, dominant in Poland, was linked to *bla*_KPC-2_ and/or *bla*_NDM-1_; Clades b and c, prevalent in Germany, carried *bla*_OXA-48_ and/or *bla*_NDM-1_; and Clade e was exclusive to Russia (Figure S3). Moreover, while most clades predominantly harbored *bla*_CTX-M-55_ (91.89%, 34/37), Clade d was uniquely associated with *bla*_CTX-M-15_ (85.71%, 6/7; *P* < 0.001), underscoring further diversification in resistance gene acquisition within this lineage.

### ST23-I exhibits higher virulence but lower antimicrobial resistance compared to ST23-II

Compared with the ST23-I lineage, isolates belonging to ST23-II exhibited significantly higher levels of antimicrobial resistance. A markedly greater proportion of ST23-II strains carried ESBL genes (93.75%, 45/48 vs. 13.68%, 152/1111) and carbapenemase genes (81.25%, 39/48 vs. 20.16%, 224/1111; both *P* < 0.001). Moreover, ST23-II universally harbored chromosomal resistance-associated mutations (GyrA p.Ser83Ile, ParC p.Ser80Ile, and OmpK36 p.Asp135_Asp136insGlyAsp) in all 48 isolates (100%), whereas these mutations were completely absent in ST23-I (0%; *P* < 0.001) (Figure 3(A)). Consistent with this, ST23-II isolates carried a substantially higher number of ARGs than ST23-I (mean: 21.71 vs. 6.09; *P* < 0.001), further supporting its enhanced resistance profile (Figure 3(B)). Temporal analysis revealed an increasing trend in ARG counts over time in the ST23-I lineage (*P* < 0.001), while no significant change was observed in ST23-II (*P* = 0.11) (Figure 3(C)). Notably, since 2007, ST23 strains carrying more than 10 ARGs have emerged and persisted, signaling a progressive accumulation of resistance determinants within this sequence type.

**Figure 3.**
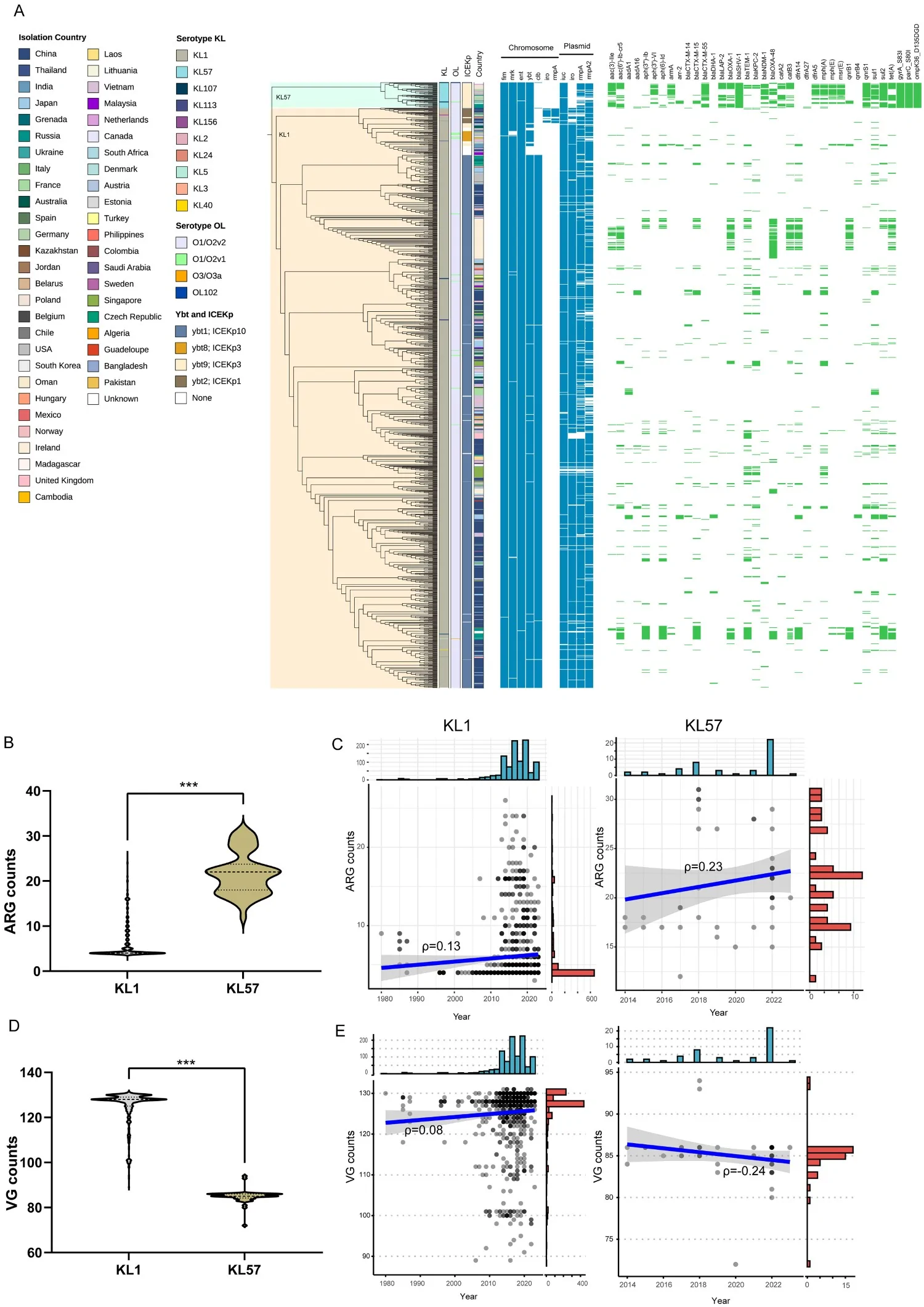
The comparisons between global ST23-KL1 and ST23-KL57 lineages. (A) Strains in ST23-KL1 and ST23-KL57 lineages displayed distinct VG and ARG profiles. (B) The ARG counts each strain comparison between ST23-KL1 and ST23-KL57 lineages. (C) Temporal distribution of ARG counts each isolate within ST23-KL1 and ST23-KL57 lineages. (D) The VG counts each strain comparison between ST23-KL1 and ST23-KL57 lineages. (E) Temporal distribution of VG counts each isolate within ST23-KL1 and ST23-KL57 lineages. ***, *P* < 0.001.

In contrast, the ST23-I lineage demonstrated markedly higher virulence potential. Based on Kleborate-based scoring incorporating the presence of *ybt*, *clb*, and *iuc* loci, 87.67% (974/1111) of ST23-I isolates achieved the maximum virulence score of 5, whereas 97.92% (47/48) of ST23-II isolates scored 4, reflecting the presence of *iuc* and *ybt* but consistent absence of *clb*. Additionally, the plasmid-borne *iro* locus was detected in 93.61% (1040/1111) of ST23-I strains but was entirely absent in ST23-II (*P* *<* 0.001) (Figure 3(A)). Quantitative analysis confirmed that ST23-I harbored significantly more virulence genes (VGs) than ST23-II (mean: 125.2 vs. 85.0; *P* < 0.001) (Figure 3(D)). Over time, VG counts in ST23-I showed a significant upward trend (*P* < 0.01), whereas ST23-II exhibited a non-significant decline (*P* = 0.10) (Figure 3(E)). Overall, ST23-I exhibited higher virulence but lower antimicrobial resistance compared to ST23-II, highlighting divergent evolutionary trajectories within the same MLST type.

### Temporospatial propagation of ST23-I lineage

We further focused on the ST23-I lineage due to its global prevalence and hypervirulent phenotype. Using BEAST, we constructed a time-scaled phylogenetic tree based on 255 representative ST23-I isolates from 12 major countries. This analysis revealed that the ST23-I lineage originated in China around 1873 (95% highest posterior density [HPD] interval: 1820–1923) (Figure 4), with a temporal signal confirmed by root-to-tip regression analysis (Figure S4). The dominant integrative and conjugative element in this lineage, ICE*Kp10*, was acquired approximately in 1931 (95% HPD: 1885–1944). Furthermore, the most recent common ancestor of the major clade, Clade A, emerged in China in 1945 (95% HPD: 1907–1955) and subsequently disseminated internationally, undergoing local diversification (Figure 4). Phylogeographic reconstruction indicated that China served as the primary source for the international spread of ST23-I between 1976 and 2011 (Figure S5).

**Figure 4.**
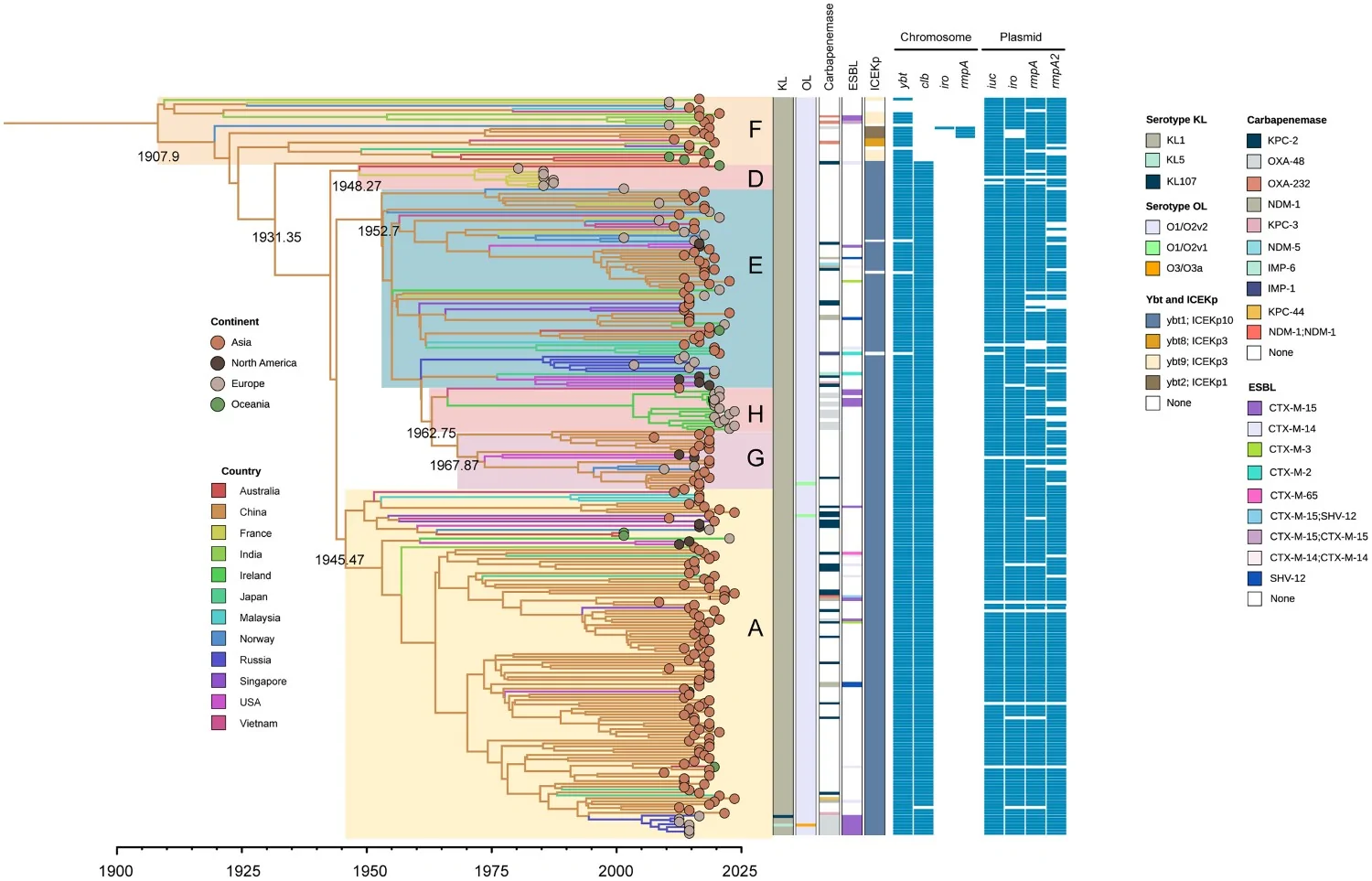
Dated phylogeny of ST23-KL1 strains from 12 major countries. Temporal phylogenetic tree of 255 representative ST23-KL1 isolates from 12 major countries, with branches colored according to the country of origin as indicated in the legend. Major clades are labeled on the right.

### Geographic segregation of carbapenemase genes in ST23-I CP-hvKP

Within the ST23-I lineage, we identified 224 CP-hvKP isolates (20.16%, 224/1111), which predominantly harbored *bla*_OXA-48_ (45.09%, 101/224), *bla*_KPC-2_ (37.05%, 83/224), *bla*_NDM-1_ (8.48%, 19/224), and *bla*_OXA-232_ (3.57%, 8/224). These strains were phylogenetically dispersed yet formed distinct clusters across the ST23-I tree (Figure 2(B)), indicative of multiple independent acquisitions and losses of carbapenemase genes within this lineage. Time-series analysis revealed that the earliest ST23-I CP-hvKP isolate was detected in 2012. Notably, *bla*_KPC-2_, the dominant carbapenemase between 2013 and 2018, was largely supplanted by *bla*_OXA-48_ from 2019 onward (Figure 5(A)). Carbapenemase distribution exhibited striking geographic segregation: *bla*_OXA-48_ predominated in Europe (92.93%, 92/99), whereas *bla*_KPC-2_ was most prevalent in Asia, North America, and South America (64.15%–85.71%) (Figure 5(B)). Additionally, *bla*_NDM-1_ (94.44%, 17/18) and *bla*_OXA-232_ (100%, 8/8) were almost exclusively confined to Asia (Figure 5(C)).

**Figure 5.**
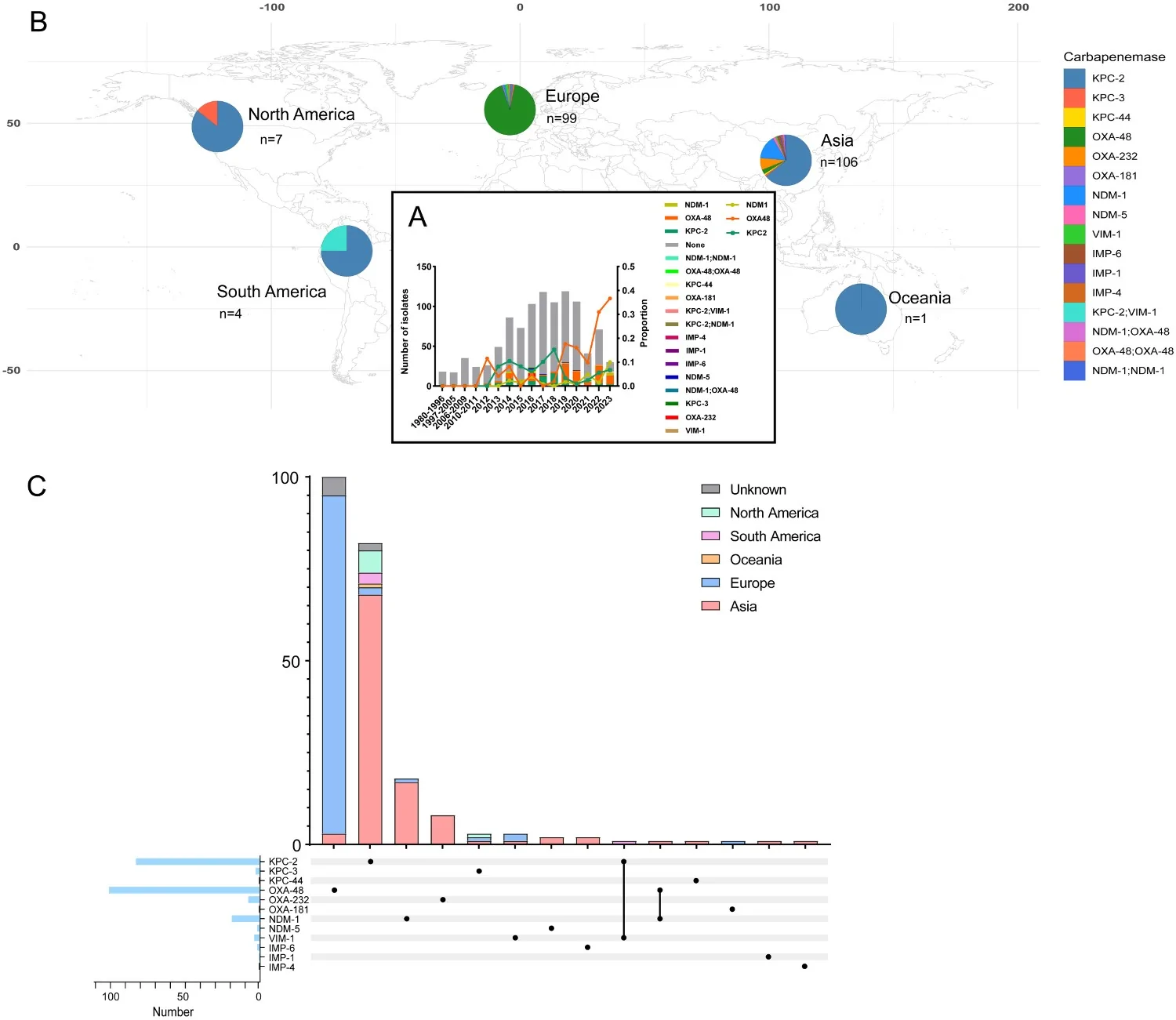
Geographic distribution and regional specificity of carbapenemases in ST23-KL1 strains. (A) Temporal distribution of all 1021 ST23-KL1 strains with available isolation dates. The exclusion of the 90 ST23-KL1 isolates was due to the absence of collection date information in the publicly available metadata. (B) Global spatial distribution of 224 ST23-KL1 CP-hvKP strains. The pie charts represent the cumulative proportions of different carbapenemase genes on each continent. (C) UpSet plot illustrating the geographic distribution of individual carbapenemase gene variants.

### Global epidemiology and conjugative frequency of carbapenemase-encoding plasmids in ST23-I CP-hvKP

To investigate the global epidemiology of carbapenemase-encoding plasmids, we characterized the plasmid replicon types among 224 ST23-I CP-hvKP isolates. Among the 101 *bla*_OXA-48_-positive genomes, the *bla*_OXA-48_ gene was exclusively located on IncL-type plasmids, predominantly identified in 77 isolates from Ireland and ten from Russia. In contrast, the 83 *bla*_KPC-2_-harboring genomes carried ten distinct plasmid types. The most prevalent was IncFII (50.60%, 42/83), with the IncFII_K2_ subtype (21.69%, 18/83; mainly 12 from China and three from the USA) and IncFII_K34_ (19.28%, 16/83; mainly 14 from China) dominating, followed by IncFII_35_ (4.82%, 4/83; all from China) (Figure 6(A)). Notably, six of the ten plasmid types were detected exclusively in China, underscoring the high evolutionary dynamism of *bla*_KPC-2_-bearing plasmids in this region. Among the 19 *bla*_NDM-1_-positive genomes (carrying 20 distinct NDM-1 plasmids), IncX3 was the dominant plasmid type (70.00%, 14/20), primarily found in 17 isolates from China.

**Figure 6.**
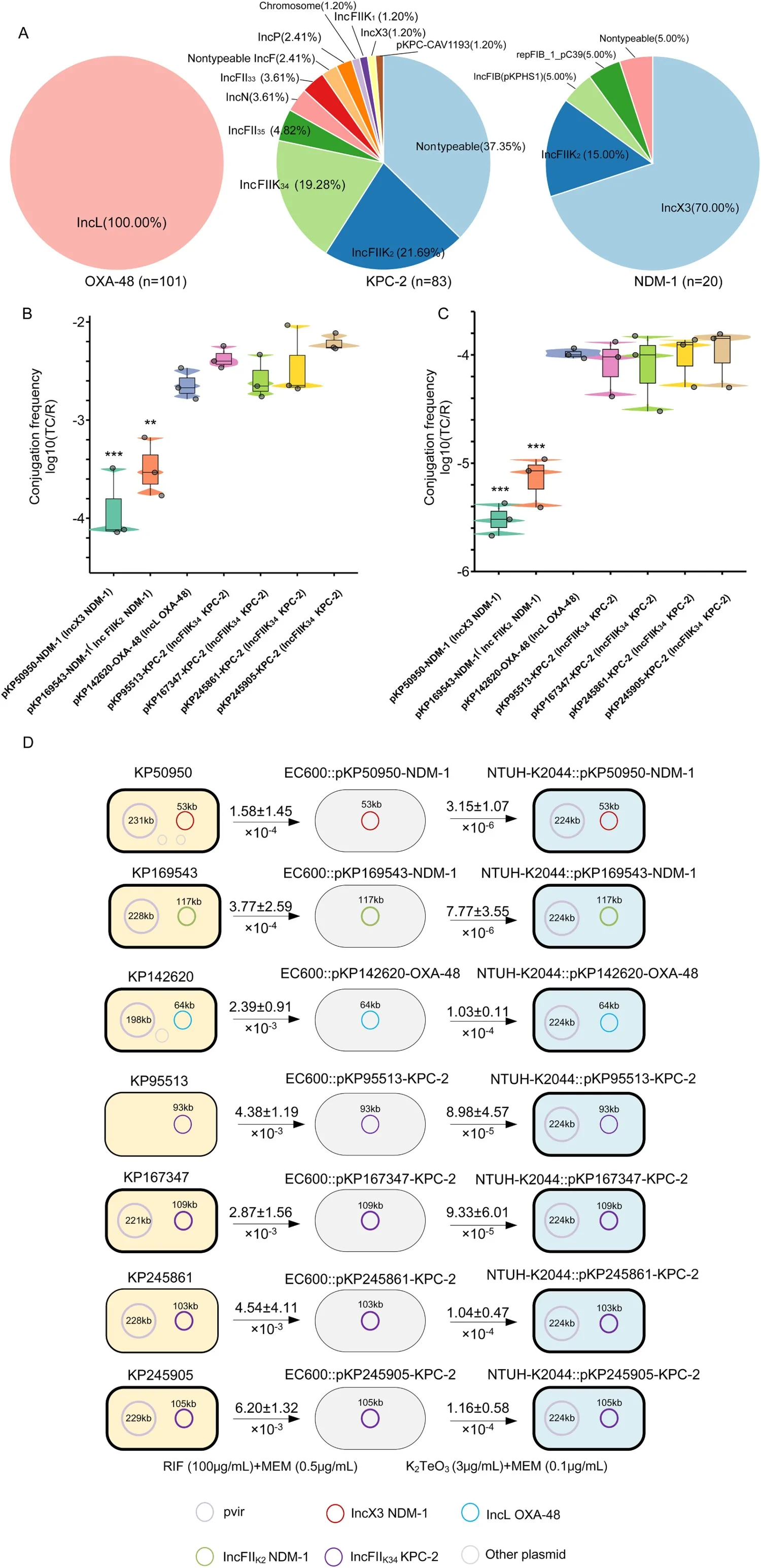
Global epidemiology and conjugative frequency of carbapenemase-encoding plasmids in ST23-KL1 CP-hvKP. (A) Distribution of carbapenemase-encoding plasmid replicons among ST23-KL1 CP-hvKP isolates. (B) Conjugation frequencies of carbapenemase-encoding plasmids from CP-hvKP clinical isolates to the recipient *E. coli* EC600. (C) Conjugation frequencies from *E. coli* EC600 (donor) to *K. pneumoniae* NTUH-K2044 (recipient). IncFII_K34_-*bla*_KPC-2_ and IncL-*bla*_OXA-48_ plasmids exhibited significantly higher transfer efficiencies than those of IncX3- and IncFII_K2_-*bla*_NDM-1_ plasmids. **, *P* < 0.01. ***, *P* < 0.001. (D) Schematic representation of the conjugation experiments. Arrow labels indicate conjugation frequencies (transconjugants per recipient). Antibiotics used for selection in each experiment are shown at the bottom: MEM, meropenem; RIF, rifampicin; K₂TeO₃, potassium tellurite.

Given the marked geographic and genetic heterogeneity of carbapenemase-encoding plasmids in ST23-I CP-hvKP, we experimentally assessed the conjugative transfer efficiency of four representative plasmids: IncX3-*bla*_NDM-1_, IncFII_K2_-*bla*_NDM-1_, IncFII_K34_-*bla*_KPC-2_, and IncL-*bla*_OXA-48_. Due to the coexistence of two distinct conjugative *bla*_NDM-1_-encoding plasmids in strain KP169509 and rifampicin resistance in KP105791, the individual conjugation frequencies of these plasmids could not be precisely quantified in matings with rifampicin-resistant *E. coli* EC600. Nevertheless, comparative conjugation assays revealed that IncFII_K34_-*bla*_KPC-2_ and IncL-*bla*_OXA-48_ plasmids exhibited significantly higher transfer efficiencies (7- to 40-fold higher) when mobilized from clinical CP-hvKP donors into *E. coli* EC600 (Figure 6(B, D)). Similarly, when transferred from EC600 transconjugants into the hvKP strain NTUH-K2044, their conjugation frequencies were 12- to 37-fold higher than those of IncX3- and IncFII_K2_-*bla*_NDM-1_ plasmids (Figure 6(C, D)).

To evaluate the *in vitro* fitness cost associated with plasmid acquisition, we assessed the competitive ability and plasmid stability of NTUH-K2044 transconjugants. Our results indicate that strains harboring IncX3-*bla*_NDM-1_, IncFII_K2_-*bla*_NDM-1_, and IncL-*bla*_OXA-48_ plasmids did not exhibit a significant fitness cost compared to the wild-type strain, as indicated by CI values close to 1 (*P* > 0.05) (Figure S6A). Conversely, the acquisition of the IncFII_K34_-*bla*_KPC-2_ plasmid resulted in a measurable fitness cost (*P* < 0.05). Furthermore, IncX3-*bla*_NDM-1_, IncFII_K2_-*bla*_NDM-1_, and IncL-*bla*_OXA-48_ plasmids were stably maintained, with 100% retention after 10 days of serial passage without selection. In contrast, IncFII_K34_-*bla*_KPC-2_ plasmids showed substantial instability, with 70–100% loss over the same period (*P* < 0.05) (Figure S6B).

### Prevalent wzc mutations in global ST23-KL1 CP-hvKP facilitated acquisition of carbapenemase plasmids

Given that high-level capsule production may inhibit plasmid transfer [42], we hypothesized that an elevated mutation burden in the capsular polysaccharide synthesis (*cps*) locus might be a key genetic determinant associated with the emergence of CP-hvKP. To test this, we compared *cps* region mutations between 224 CP-hvKP and 874 non-CP-hvKP ST23-KL1 isolates, excluding 13 isolates with alternative KL types from the ST23-I clade due to significant *cps* genetic divergence. Using the KL1 *cps* locus of the hypervirulent reference strain NTUH-K2044 as a baseline, we identified 703 mutations in the *cps* locus of CP-hvKP isolates and 3092 mutations in non-CP-hvKP isolates. This corresponds to average mutation frequencies of 3.14 and 3.54 mutations per strain, respectively. Notably, CP-hvKP strains exhibited a higher proportion of non-synonymous mutations in KL1 protein-coding genes than non-CP-hvKP strains (2.38 vs. 2.32 per strain), suggesting an increased likelihood of functionally consequential alterations in capsule biosynthesis among CP-hvKP isolates. Further analysis revealed *wzc*, encoding a tyrosine autokinase essential for capsule export, as a prominent mutation hotspot in CP-hvKP relative to non-CP-hvKP (Figure 7(A) and Figure S7A). Using Fisher’s exact test, we identified 42 SNP sites with significantly different frequencies between CP-hvKP and non-CP-hvKP (P < 0.05; Figure 7(B)), 40 of which were more prevalent in CP-hvKP. After excluding synonymous SNPs and those located in intergenic regions, 29 non-synonymous SNPs remained. Notably, 11 of them were located in the *wzc* gene. Mapping the geographic origins of isolates carrying these significant non-synonymous SNPs revealed marked geographic specificity (Figure 7(C)). For instance, the CP-hvKP-enriched variant *wzc*^2042A^ ^>^ ^G^ (p.Glu681Gly) was predominantly found in Ireland, whereas *wzc*^1738T^ ^>^ ^A^ (p.Ser580Thr) and *wzc*^112A^ ^>^ ^T^ (p.Thr38Ser) were predominantly detected in China. To account for potential lineage expansion within ST23-KL1, *P*-values from Fisher’s exact test were subjected to Bonferroni multiple-testing corrections. Variants retaining significant (*wzc*^2042A^ ^>^ ^G^ and *wzc*^1738T^ ^>^ ^A^) were selected as candidates and subsequently subjected to experimental verification.

**Figure 7.**
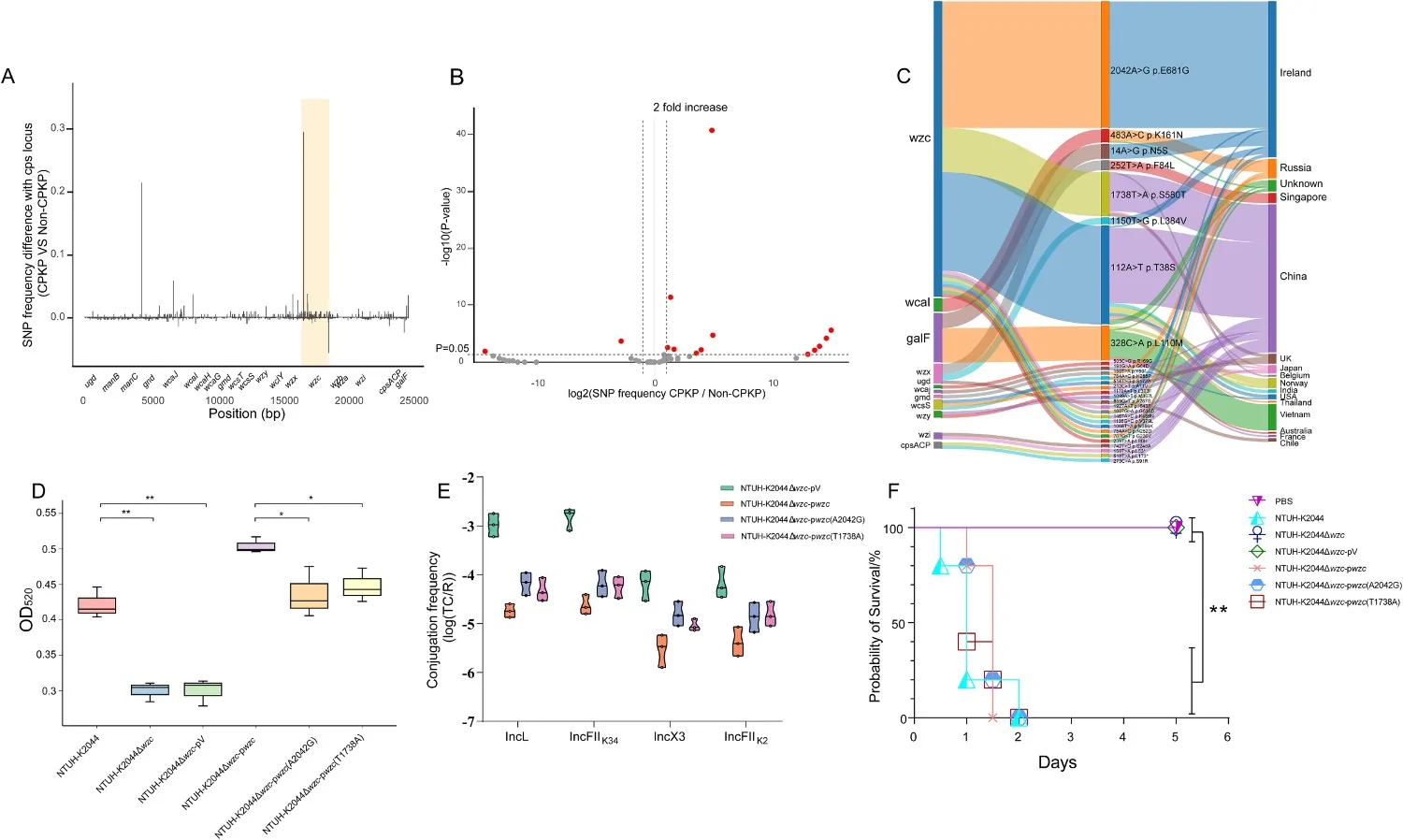
Elevated capsule mutation frequency correlates with CP-hvKP ST23. (A) Comparative analysis of SNP frequency differences between CP-hvKP and non-CP-hvKP isolates. The light orange shaded region denotes the *wzc* gene within the *cps* locus. (B) Volcano plot depicting genomic positions with significantly different SNP frequencies between CP-hvKP and non-CP-hvKP, as determined by Fisher’s exact test. The dashed vertical line indicates a twofold increase or decrease in mutation frequency in CP-hvKP relative to non-CP-hvKP. (C) Sankey diagram illustrating the geographic distribution of significant SNP positions. (D) Quantification of uronic acid in NTUH-K2044, its isogenic Δ*wzc* mutant, and complemented strains carrying either an empty vector, wild-type *wzc*, or mutated *wzc* alleles (*wzc*^2042A^ ^>^ ^G^ and *wzc*^1738T^ ^>^ ^A^). (E) Conjugation frequencies of NTUH-K2044 strains expressing different *wzc* variants. Donor strain: *E. coli* EC600 harboring various carbapenemase-encoding plasmids. (F) Mortality and survival dynamics of mice infected intraperitoneally with NTUH-K2044 strains harboring different *wzc* mutations. *, *P* < 0.05. **, *P* < 0.01.

To assess the phenotypic impact of the two prevalent non-synonymous variants, *wzc*^2042A^ ^>^ ^G^ (p.Glu681Gly) and *wzc*^1738T^ ^>^ ^A^ (p.Ser580Thr), we generated an isogenic Δ*wzc* mutant in the hypervirulent ST23-KL1 strain NTUH-K2044 and performed allelic complementation with: (i) wild-type *wzc*, (ii) *wzc* harboring either the p.Glu681Gly or p.Ser580Thr substitution, or (iii) an empty vector control. Multiple sequence alignment of Wzc proteins across diverse *K. pneumoniae* capsular types revealed that residues E681 and S580 are evolutionarily conserved in Wzc (Figure S7B), underscoring their potential functional importance. Uronic acid quantification and Alcian blue staining showed that strains complemented with either *wzc*^2042A^ ^>^ ^G^ or *wzc*^1738T^ ^>^ ^A^ produced significantly less CPS than those complemented with wild-type *wzc* (*P* < 0.05). In contrast, the empty vector control exhibited CPS levels comparable to those of the Δ*wzc* mutant (*P* > 0.05) and significantly lower than all *wzc*-complemented strains (*P* < 0.05), confirming that both the p.Glu681Gly and p.Ser580Thr variants retain partial Wzc function (Figure 7(D) and Figure S7C). Furthermore, when serving as recipients in plasmid transfer assays using *E. coli* EC600 donors carrying four different carbapenemase plasmids, both *wzc* variant mutants displayed significantly higher conjugation frequencies than the wild-type *wzc*-complemented strain (Two-way ANONA, *P* < 0.05; Figure 7(E)). Additionally, electrotransformation assays revealed that both *wzc* mutants showed a slightly higher transformation efficiency compared to the wild-type (Figure S7D). Collectively, these findings suggest that prevalent *wzc* mutations may enhance the ability of hvKP to acquire epidemic carbapenemase-encoding plasmids.

To investigate whether *wzc* mutations affect the virulence potential and impose a fitness cost on ST23 *K. pneumoniae*, we conducted murine infection models and *in vitro* competition assays. *In vivo* infection experiments demonstrated that all mice infected with NTUH-K2044Δ*wzc* or its empty vector control survived throughout the monitoring period. In contrast, both the *wzc* mutant-complemented strains and the wild-type *wzc*-complemented strain exhibited 100% mortality (*P* > 0.05), comparable to that of the parental NTUH-K2044 strain (Figure 7(F)). Furthermore, *in vitro* competition assays revealed that the *wzc* mutant-complemented strains displayed competitive fitness comparable to that of the wild-type *wzc*-complemented strain under antibiotic-free LB conditions (*P* > 0.05). (Figure S7E). Collectively, these results indicate that the *wzc* mutations neither compromise the hypervirulent phenotype of NTUH-K2044 nor impose a significant fitness cost.

## Discussion

Our comprehensive genomic epidemiological investigation of global ST23 *K. pneumoniae* isolates, coupled with functional validation, provided a crucial insight into the evolution of CP-hvKP. We identified a markedly elevated frequency of capsule-related *wzc* mutations as a crucial and widespread mechanism that could facilitate the acquisition of epidemic carbapenemase-encoding plasmids by globally dominant ST23-KL1 hvKP lineage. This adaptive trait enabled the emergence of difficult-to-treat invasive infections, posing a serious threat to public health.

This work significantly advances our understanding beyond previous studies that have primarily described the phylogeny or clinical features of individual CP-hvKP isolates [43,44]. By constructing a large-scale, temporospatially resolved dataset, we were able to delineate two distinct evolutionary trajectories within ST23. While ST23-I was predominantly hypervirulent and Asian in origin, the minor ST23-II sublineage, concentrated in Europe, exhibited higher antimicrobial resistance burdens, including chromosomal mutations and a near-ubiquitous ESBL/carbapenemase profile. This stark divergence, despite identical MLST designations, underscored the limitations of sequence typing alone and emphasized the need for serotype- and plasmid-aware surveillance [45]. In addition, we observed divergent temporal trends in resistance and virulence evolution between the two lineages. While ST23-I showed a significant accumulation of ARGs and VGs over time, ST23-II exhibited a resistance and virulence profile with no significant temporal trend. This absence of a detectable trend in ST23-II may be attributed to limited statistical power due to the small sample size (n = 48). Future studies with a larger collection of ST23-II isolates are warranted to validate these evolutionary dynamics.

Our temporal phylogeny placed the origin of ST23-KL1 in China around 1873, with international dissemination beginning in the late twentieth century. It is important to note that the root-to-tip regression analysis revealed a limited temporal signal (R^2^ = 0.2171) in the current dataset. This weak temporal structure indicates that the molecular clock signal lacks the strength to precisely resolve the timing of all divergence events. Consequently, this uncertainty is reflected in the wide 95% Highest Posterior Density (HPD) intervals of our tMRCA estimates (1820–1923). Although this limits the precision of the dating, the estimated emergence of ST23-KL1 in the late 19th to early twentieth century is consistent with previous studies [23,46], supporting the general validity of the phylogenetic reconstruction despite the dating uncertainty. In addition, isolates from China accounted for 39.3% of the total collection, reflecting a regional sampling bias rather than a strictly randomized global surveillance. Although the downsizing strategy employed for phylogenetic reconstruction was necessary to ensure computational tractability, it introduces a potential sampling bias. This imbalance, particularly the overrepresentation of Chinese isolates, may influence the resolution of phylogeographical inferences and could lead to an ascertainment bias. Future studies incorporating more balanced, longitudinal sampling across diverse regions are warranted to validate these phylogeographical trajectories.

We demonstrated that IncL-*bla*_OXA-48_ and IncFII_K34_-*bla*_KPC-2_ plasmids exhibited markedly higher conjugative efficiency (up to 40-fold) than IncX3- or IncFII_K2_-*bla*_NDM-1_ plasmids in both *E. coli* and *K. pneumoniae* recipients. This superior transfer capacity likely contributes to their global predominance, with OXA-48 plasmids dominating in Europe and KPC-2 variants prevailing in Asia and the Americas. A previous study identified the IncFII_K34_ *bla*_KPC-2_ plasmid as the most common KPC plasmid in CR-hvKP, showing higher conjugation frequency than the epidemic IncFII_K2_ *bla*_KPC-2_ plasmid [47]. Our findings further revealed that the IncFII_K34_-*bla*_KPC-2_ plasmid likely achieved widespread prevalence through rapid dissemination rather than long-term maintenance, despite the fitness cost and instability observed during serial passage.

Concurrently, we identified an elevated burden of non-synonymous mutations in the *cps* locus (particularly in *wzc*, a key regulator of CPS export [48]) within ST23-KL1 CP-hvKP. The geographic clustering of specific *wzc* alleles [e.g. *wzc*^2042A^ ^>^ ^G^ (p.Glu681Gly) in Ireland, *wzc*^1738T^ ^>^ ^A^ (p.Ser580Thr) in China] suggested parallel evolution under region-specific selective pressures. Due to the technical challenges in constructing scarless single-nucleotide mutants on the chromosome of the wild-type *K. pneumoniae* NTUH-K2044 strain, we employed a plasmid-based complementation system for our functional validation. While our functional validation demonstrated that both *wzc*^2042A^ ^>^ ^G^ and *wzc*^1738T^ ^>^ ^A^ mutations were associated with attenuated CPS production and concurrently enhanced conjugation efficiency of carbapenemase plasmids, the precise molecular mechanism linking these phenotypes remains to be fully elucidated. Previous studies have indicated that specific mutations in *wzc* can either reduce CPS production or, conversely, lead to a hypercapsule/hypermucoviscous phenotype [48–50]. Importantly, reduced CPS has been associated with improved plasmid transfer efficiency both *in vitro* and in the gut environment [51,52]. Our data align with these findings, suggesting that the observed *wzc* mutations likely lead to a reduction in capsule. We postulate that the observed reduction in CPS likely diminishes physical barriers to conjugation, such as steric hindrance caused by the dense polysaccharide layer, thereby facilitating horizontal gene transfer. Although alternative mechanisms cannot be ruled out, this model provides a plausible explanation for how hvKP acquires carbapenem resistance without completely sacrificing its capsule or pathogenic potential. Together, these findings imply that the observed *wzc* mutations represent adaptive changes that enabled ST23-KL1 to transition toward a successful carbapenem-resistant phenotype.

In summary, elevated *wzc* mutation frequency represented an adaptive evolutionary pathway that facilitated carbapenemase acquisition while maintaining virulence in hvKP ST23. Collectively, these findings illustrated that the interplay between capsule-associated chromosomal mutations and plasmid-mediated horizontal gene transfer shaped the resistance evolutionary adaptation of this clinically important pathogen.

## Supplementary Material

Supplementary materials.pdf

## Data Availability

The whole-genome sequences of the ten isolates newly sequenced in this study are available for download from NCBI under BioProject accession number PRJNA1206662.

## References

[1] Pendleton JN, Gorman SP, Gilmore BF. Clinical relevance of the ESKAPE pathogens. Expert Rev Anti Infect Ther. 2013;11(3):297–308. doi:10.1586/eri.13.12 23458769

[2] Tzouvelekis LS, Markogiannakis A, Psichogiou M, et al. Carbapenemases in *Klebsiella pneumoniae* and other Enterobacteriaceae: an evolving crisis of global dimensions. Clin Microbiol Rev. 2012;25(4):682–707. doi:10.1128/CMR.05035-11 PMC3485753 23034326

[3] Chen T, Ying L, Xiong L, et al. Understanding carbapenem-resistant hypervirulent *Klebsiella pneumoniae*: key virulence factors and evolutionary convergence. hLife. 2024;2(12):611–624. doi:10.1016/j.hlife.2024.06.005

[4] Han X, Yao J, He J, et al. Clinical and laboratory insights into the threat of hypervirulent *Klebsiella pneumoniae*. Int J Antimicrob Agents. 2024;64(3):107275. doi:10.1016/j.ijantimicag.2024.107275 39002700

[5] Alaimo C, Karaky N, Lawrence R, et al. Safety and immunogenicity of a *Klebsiella pneumoniae* tetravalent bioconjugate vaccine (Kleb4 V) administered to healthy adults: a first-in-human phase I/II randomized and controlled study. J Infect Dis. 2026;233(2):e342–e351. doi:10.1093/infdis/jiaf600 PMC13017035 41289032

[6] Douradinha B. Exploring the journey: a comprehensive review of vaccine development against *Klebsiella pneumoniae*. Microbiol Res. 2024;287:127837. doi:10.1016/j.micres.2024.127837 39059097

[7] Assoni L, Girardello R, Converso TR, et al. Current stage in the development of *Klebsiella pneumoniae* vaccines. Infect Dis Ther. 2021;10(4):2157–2175. doi:10.1007/s40121-021-00533-4 PMC8412853 34476772

[8] Chen T, Wang Y, Zhou Y, et al. Recombination drives evolution of carbapenem-resistant *Klebsiella pneumoniae* sequence type 11 KL47 to KL64 in China. Microbiol Spectr. 2023;11(1):e0110722. doi:10.1128/spectrum.01107-22 PMC9927301 36622219

[9] Luo Y, Wang Y, Ye L, et al. Molecular epidemiology and virulence factors of pyogenic liver abscess causing *Klebsiella pneumoniae* in China. Clin Microbiol Infect. 2014;20(11):O818–O824. doi:10.1111/1469-0691.12664 24804560

[10] Yan W, Xu D, Shen Y, et al. Molecular epidemiology of string test-positive *Klebsiella pneumoniae* isolates in Huzhou, China, 2020-2023. Front Cell Infect Microbiol. 2024;14:1411658. doi:10.3389/fcimb.2024.1411658 PMC11333340 39165917

[11] Wyres KL, Lam MMC, Holt KE. Population genomics of *Klebsiella pneumoniae*. Nat Rev Microbiol. 2020;18(6):344–359. doi:10.1038/s41579-019-0315-1 32055025

[12] Zhou H, Guo C, Cui Z, et al. The epidemiology and hypervirulence of *Klebsiella pneumoniae* ST23 unveil epidemic risks in China and worldwide. Lancet Reg Health West Pac. 2025;62:101661. doi:10.1016/j.lanwpc.2025.101661 PMC12398865 40896369

[13] Gu Y, Wang X, Zhang W, et al. Dissemination of bla(NDM)-harboring plasmids in carbapenem-resistant and hypervirulent *Klebsiella pneumoniae*. Microbiol Spectr. 2025;13(3):e0196824. doi:10.1128/spectrum.01968-24 PMC11878072 39936929

[14] European Centre for Disease Prevention and Control. Emergence of hypervirulent *Klebsiella pneumoniae* ST23 carrying carbapenemase genes in EU/EEA countries, first update. 14 February 2024. ECDC: Stockholm; 2024.

[15] Maguire M, DeLappe N, Clarke C, et al. Genomic and phylogenetic analysis of hypervirulent *Klebsiella pneumoniae* ST23 in Ireland. Microb Genom. 2025;11(3):001373. doi:10.1099/mgen.0.001373 PMC12452180 40106330

[16] Biedrzycka M, Izdebski R, Urbanowicz P, et al. MDR carbapenemase-producing *Klebsiella pneumoniae* of the hypervirulence-associated ST23 clone in Poland, 2009-19. J Antimicrob Chemother. 2022;77(12):3367–3375. doi:10.1093/jac/dkac326 36177793

[17] World Health Organization. Disease outbreak news; antimicrobial resistance, hypervirulent *Klebsiella pneumoniae* - global situation; 2024. [accessed 2025 May 20]. Available at: https://www.who.int/emergencies/disease-outbreak-news/item/2024-DON527.

[18] Tang N, Li Y, Yao S, et al. Epidemicity and clonal replacement of hypervirulent carbapenem-resistant *Klebsiella pneumoniae* with diverse pathotypes and resistance profiles in a hospital. J Glob Antimicrob Resist. 2023;32:4–10. doi:10.1016/j.jgar.2022.11.007 36400407

[19] Chen T, Wang X, Xiong L, et al. Emergence and molecular evolution of carbapenem-resistant hypervirulent ST23 *Klebsiella pneumoniae*: the superbug phenomenon in China. Virulence. 2025;16(1):2545556. doi:10.1080/21505594.2025.2545556 PMC12351723 40802580

[20] Karlsson M, Stanton RA, Ansari U, et al. Identification of a carbapenemase-producing hypervirulent *Klebsiella pneumoniae* isolate in the United States. Antimicrob Agents Chemother. 2019;63(7):e00519–19. doi:10.1128/AAC.00519-19 PMC6591612 31061159

[21] Dong N, Sun Q, Huang Y, et al. Evolution of carbapenem-resistant serotype K1 hypervirulent *Klebsiella pneumoniae* by acquisition of bla(VIM-1)-bearing plasmid. Antimicrob Agents Chemother. 2019;63(9):e01056–19. doi:10.1128/AAC.01056-19 PMC6709456 31307980

[22] Lin YT, Chuang C, Chou SH, et al. Emergence of OXA-48-producing hypervirulent *Klebsiella pneumoniae* strains in Taiwan. Eur J Clin Microbiol Infect Dis. 2024;43(2):389–393. doi:10.1007/s10096-023-04733-3 38062176

[23] Lam MMC, Wyres KL, Duchêne S, et al. Population genomics of hypervirulent *Klebsiella pneumoniae* clonal-group 23 reveals early emergence and rapid global dissemination. Nat Commun. 2018;9(1):2703. doi:10.1038/s41467-018-05114-7 PMC6045662 30006589

[24] Tian D, Liu X, Chen W, et al. Prevalence of hypervirulent and carbapenem-resistant *Klebsiella pneumoniae* under divergent evolutionary patterns. Emerg Microbes Infect. 2022;11(1):1936–1949. doi:10.1080/22221751.2022.2103454 PMC9359173 35844192

[25] Wu Y, Pu F, Yan Z, et al. Geographic containment and virulence-resistance trade-offs drive the evolution of hypervirulent *Klebsiella pneumoniae*. iMeta. 2025;4(5):e70077. doi:10.1002/imt2.70077 PMC12528002 41112046

[26] Lam MMC, Wick RR, Watts SC, et al. A genomic surveillance framework and genotyping tool for *Klebsiella pneumoniae* and its related species complex. Nat Commun. 2021;12(1):4188. doi:10.1038/s41467-021-24448-3 PMC8263825 34234121

[27] Seemann T. Prokka: rapid prokaryotic genome annotation. Bioinformatics. 2014;30(14):2068–2069. doi:10.1093/bioinformatics/btu153 24642063

[28] Wick RR, Judd LM, Gorrie CL, et al. Unicycler: resolving bacterial genome assemblies from short and long sequencing reads. PLoS Comput Biol. 2017;13(6):e1005595. doi:10.1371/journal.pcbi.1005595 PMC5481147 28594827

[29] Jolley KA, Bray JE, Maiden MCJ. Open-access bacterial population genomics: BIGSdb software, the PubMLST. org website and their applications. Wellcome Open Res. 2018;3:124. doi:10.12688/wellcomeopenres.14826.1 PMC6192448 30345391

[30] Feldgarden M, Brover V, Haft DH, et al. Validating the AMRFinder tool and resistance gene database by using antimicrobial resistance genotype-phenotype correlations in a collection of isolates. Antimicrob Agents Chemother. 2019;63(11):e00483–19. doi:10.1128/AAC.00483-19 PMC6811410 31427293

[31] Robertson J, Nash JHE. MOB-suite: software tools for clustering, reconstruction and typing of plasmids from draft assemblies. Microb Genom. 2018;4(8):e000206. doi:10.1099/mgen.0.000206 PMC6159552 30052170

[32] Tonkin-Hill G, Lees JA, Bentley SD, et al. RhierBAPS: an R implementation of the population clustering algorithm hierBAPS. Wellcome Open Res. 2018;3:93. doi:10.12688/wellcomeopenres.14694.1 PMC6178908 30345380

[33] Minh BQ, Schmidt HA, Chernomor O, et al. IQ-TREE 2: new models and efficient methods for phylogenetic inference in the genomic era. Mol Biol Evol. 2020;37(5):1530–1534. doi:10.1093/molbev/msaa015 PMC7182206 32011700

[34] Drummond AJ, Rambaut A. BEAST: Bayesian evolutionary analysis by sampling trees. BMC Evol Biol. 2007;7:214. doi:10.1186/1471-2148-7-214 PMC2247476 17996036

[35] Bielejec F, Baele G, Vrancken B, et al. Spread3: interactive visualization of spatiotemporal history and trait evolutionary processes. Mol Biol Evol. 2016;33(8):2167–2169. doi:10.1093/molbev/msw082 PMC6398721 27189542

[36] Chen T, Wang Y, Chi X, et al. Genetic, virulence, and antimicrobial resistance characteristics associated with distinct morphotypes in ST11 carbapenem-resistant *Klebsiella pneumoniae*. Virulence. 2024;15(1):2349768. doi:10.1080/21505594.2024.2349768 PMC11093053 38736039

[37] Chen T, Wang X, Xiong L, et al. Genomic analyses identify nosocomial transmission of ST23 carbapenem-resistant hypervirulent *Klebsiella pneumoniae* mediated by a conjugative IncFII(K2) NDM-1 plasmid. Virulence. 2026;17(1):2668167. doi:10.1080/21505594.2026.2668167 PMC13138083 42070149

[38] Wang Y, Luo Q, Chen T, et al. Clinical, biological and genome-wide comparison of carbapenem-resistant *Klebsiella pneumoniae* with susceptibility transformation to polymyxin B during therapy. Clin Microbiol Infect. 2023;29(10):1336 e1–1336 e8. doi:10.1016/j.cmi.2023.06.029 37423426

[39] Hu W, Wang Y, Yang B, et al. Bacterial YedK represses plasmid DNA replication and transformation through its DNA single-strand binding activity. Microbiol Res. 2021;252:126852. doi:10.1016/j.micres.2021.126852 34454309

[40] Ruiz E, Ocampo-Sosa AA, Rezusta A, et al. Acquisition of carbapenem resistance in multiresistant *Klebsiella pneumoniae* strains harbouring blaCTX-M-15, qnrS1 and aac(6’)-Ib-cr genes. J Med Microbiol. 2012;61(Pt 5):672–677. doi:10.1099/jmm.0.038083-0 22282462

[41] Ye J, Wang Y, Xiong LY, et al. Comparison of clinical and microbiological characteristics of community-acquired hypervirulent *Klebsiella pneumoniae* liver abscess in diabetic and non-diabetic patients. Yi Chuan. 2023;45(11):1052–1061. 10.16288/j.yczz.23-167 38764270

[42] Wyres KL, Wick RR, Judd LM, et al. Distinct evolutionary dynamics of horizontal gene transfer in drug resistant and virulent clones of *Klebsiella pneumoniae*. PLoS Genet. 2019;15(4):e1008114. doi:10.1371/journal.pgen.1008114 PMC6483277 30986243

[43] Xin L, Wang J, Qin C, et al. Emergence of ST23-K1 bla(KPC-3)-positive hypervirulent carbapenem-resistant *Klebsiella pneumoniae* from China: a molecular, biological, and epidemiological study. J Glob Antimicrob Resist. 2025;45:234–239. doi:10.1016/j.jgar.2025.09.007 41077277

[44] Shi Q, Huang W, Hu D, et al. The nationwide genomic characteristics and phylogenetic evolution of ST23-K1 hypervirulent *Klebsiella pneumoniae* in relation to virulence and antimicrobial resistance acquisition. J Infect. 2026;92(3):106709. doi:10.1016/j.jinf.2026.106709 41720308

[45] Lam MMC, Holt KE, Wyres KL. Comment on: MDR carbapenemase-producing *Klebsiella pneumoniae* of the hypervirulence-associated ST23 clone in Poland, 2009-19. J Antimicrob Chemother. 2023;78(4):1132–1134. doi:10.1093/jac/dkad028 PMC10068415 36760082

[46] Jiang X, Li S, Li C, et al. The global genomic landscape of hypervirulent *Klebsiella pneumoniae* from 1932 to 2021. mLife. 2025;4(4):378–396. doi:10.1002/mlf2.70029 PMC12395583 40893980

[47] Jiang J, Wang L, Hu Y, et al. Global emergence of carbapenem-resistant hypervirulent *Klebsiella pneumoniae* driven by an IncFII(K34) KPC-2 plasmid. EBioMedicine. 2025;113:105627. doi:10.1016/j.ebiom.2025.105627 PMC11919442 40024096

[48] Ernst CM, Braxton JR, Rodriguez-Osorio CA, et al. Adaptive evolution of virulence and persistence in carbapenem-resistant *Klebsiella pneumoniae*. Nat Med. 2020;26(5):705–711. doi:10.1038/s41591-020-0825-4 PMC9194776 32284589

[49] He J, Shi Q, Chen Z, et al. Opposite evolution of pathogenicity driven by in vivo wzc and wcaJ mutations in ST11-KL64 carbapenem-resistant *Klebsiella pneumoniae*. Drug Resist Updat. 2023;66:100891. doi:10.1016/j.drup.2022.100891 36427451

[50] Teng G, Qin Q, Ding S, et al. The *wzc* mutation mediates virulence changes in K1-type *Klebsiella pneumoniae* within the same patient. Front Microbiol. 2025;16:1577629. doi:10.3389/fmicb.2025.1577629 PMC12119577 40444005

[51] Yong M, Low WW, Mishra S, et al. Differential gut transmission of IncP plasmid clades involving hypervirulent *Klebsiella pneumoniae* reveals plasmid-specific ecological adaptation. Nat Commun. 2025;16(1):11353. doi:10.1038/s41467-025-66413-4 PMC12728208 41372160

[52] Wei DW, Song Y, Li Y, et al. Insertion sequences accelerate genomic convergence of multidrug resistance and hypervirulence in *Klebsiella pneumoniae* via capsular phase variation. Genome Med. 2025;17(1):45. doi:10.1186/s13073-025-01474-0 PMC12057282 40329368

