# Supplementary material for: Elevated wzc mutation frequency promotes carbapenem resistance evolution in hypervirulent Klebsiella pneumoniae ST23
Source: Emerg Microbes Infect. 2026 Aug 27;15(1):2726043. doi: 10.1080/22221751.2026.2726043 (PMC13647403; doi:10.1080/22221751.2026.2726043)
Supplement: Supplementary file 1 [file TEMI_A_2726043_SM0874.pdf]

Supplementary Materials

Table of contents

Supplementary Figures

Figure S1. Partial sequence alignments of the *ompK36* (A) and *ompK37* (B) genes in strain KP136096 against their respective reference sequences.

Figure S2. The KL geographic distribution (A) and phylogenetic analysis (B) of 1159 ST23 *K. pneumoniae* isolates.

Figure S3. Maximum-likelihood phylogenetic tree of 48 *K. pneumoniae* isolates in ST23-KL57 lineage.

Figure S4. Temporal signal analysis of the ST23-K1 lineage.

Figure S5. Phylogeographic analysis of ST23-KL1 strains from 12 major countries.

Figure S6. *In vitro* competitive ability of NTUH-K2044 transconjugants (A) and the stability of carbapenemase-encoding plasmids (B).

Figure S7. Genetic variation of the *cps* locus and the role of *wzc* variant in the phenotypic divergence of NTUH-K2044.

Supplementary Tables

Table S1. Plasmids and primers for construction of mutant and complementation strains.

Table S2. Minimum inhibitory concentrations (MICs) of commonly used antibiotics against ten ST23 CRKP strains.

Table S3. Genomic characteristics of ten ST23 BSI CRKP strains.

Table S4. Metadata and genotype of all ST23 genomes.

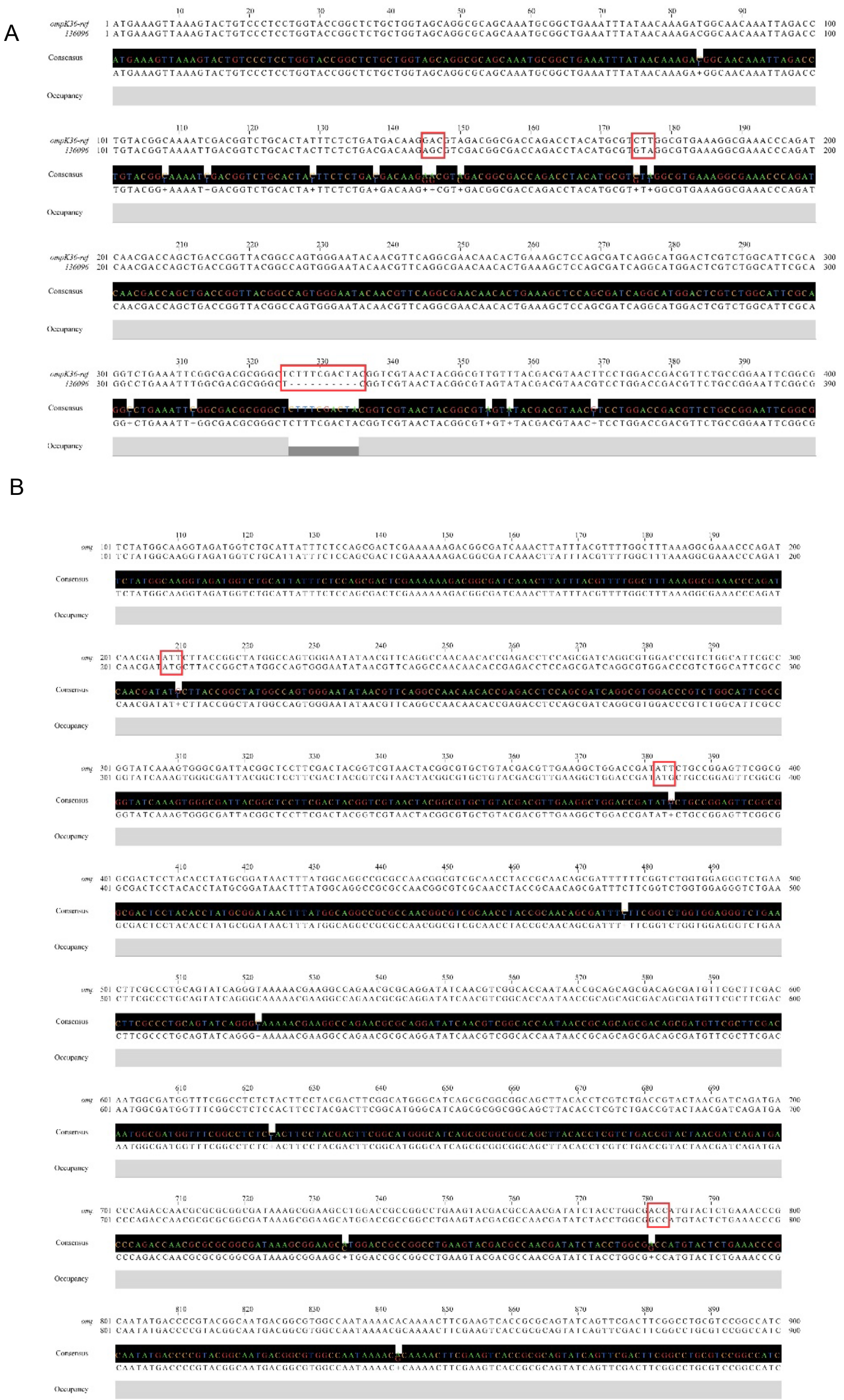

Figure S1. Partial sequence alignments of the *ompK36* (A) and *ompK37* (B) genes in strain KP136096 against their respective reference sequences (Accession: Z33506.1 for *ompK36* and AJ011502.1 for *ompK37*). For each panel, the reference sequence is shown on the upper line and the corresponding sequence from KP136096 is on the lower line. The red rectangle highlights a region where mutations are present.

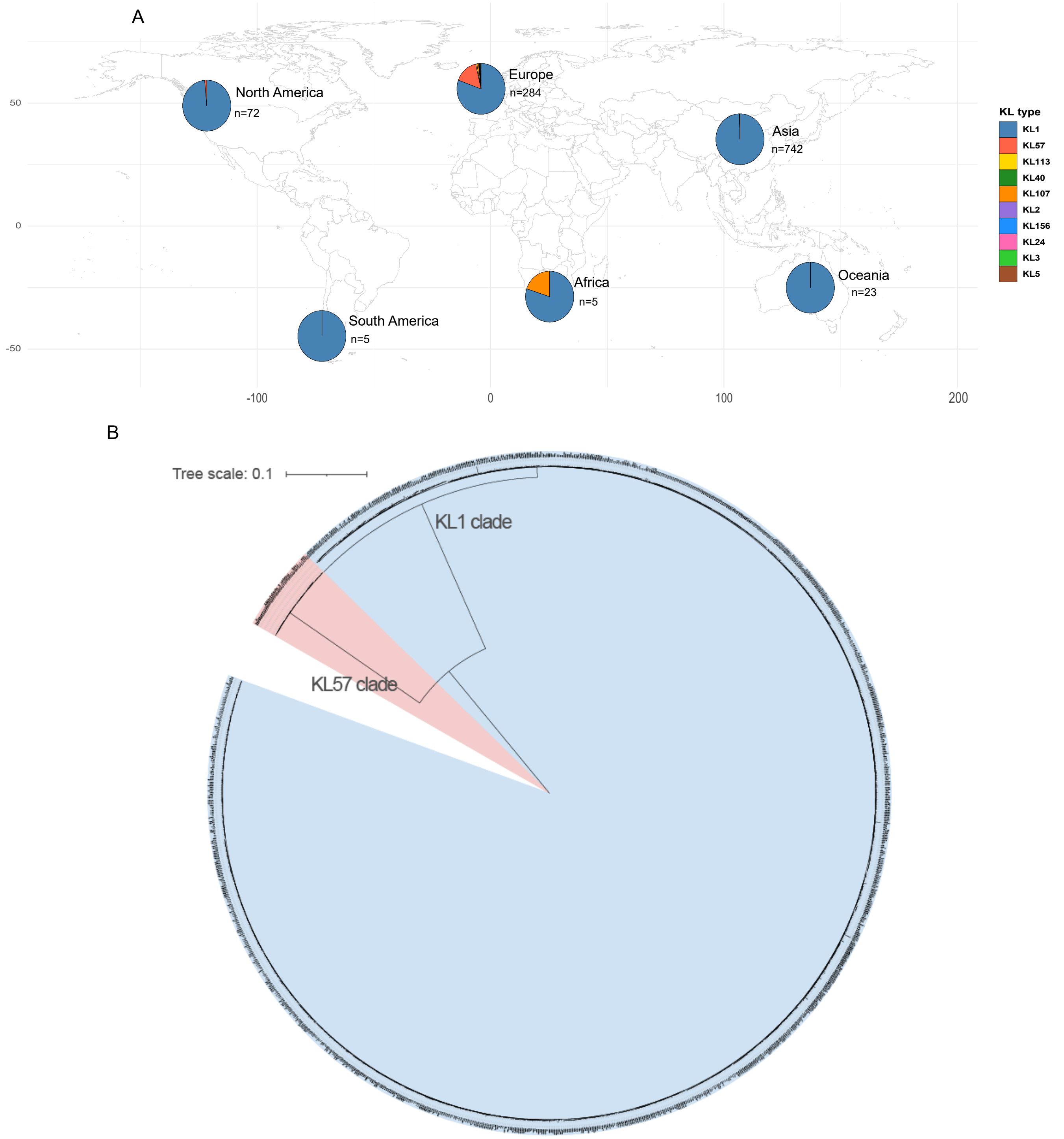

Figure S2. The KL geographic distribution (A) and phylogenetic analysis (B) of 1159 ST23 *K. pneumoniae* isolates. This phylogenetic tree illustrates the polyphyletic nature of ST23 *K. pneumoniae*. The largest clade, designated ST23-KL1 (highlighted in blue), contains the majority of isolates and predominantly consists of ST23-KL1 strains (n = 1111). A secondary clade, labeled ST23-KL57 (highlighted in pink), represents distinct phylogenetic branch primarily comprising ST23-KL57 isolates (n = 48).

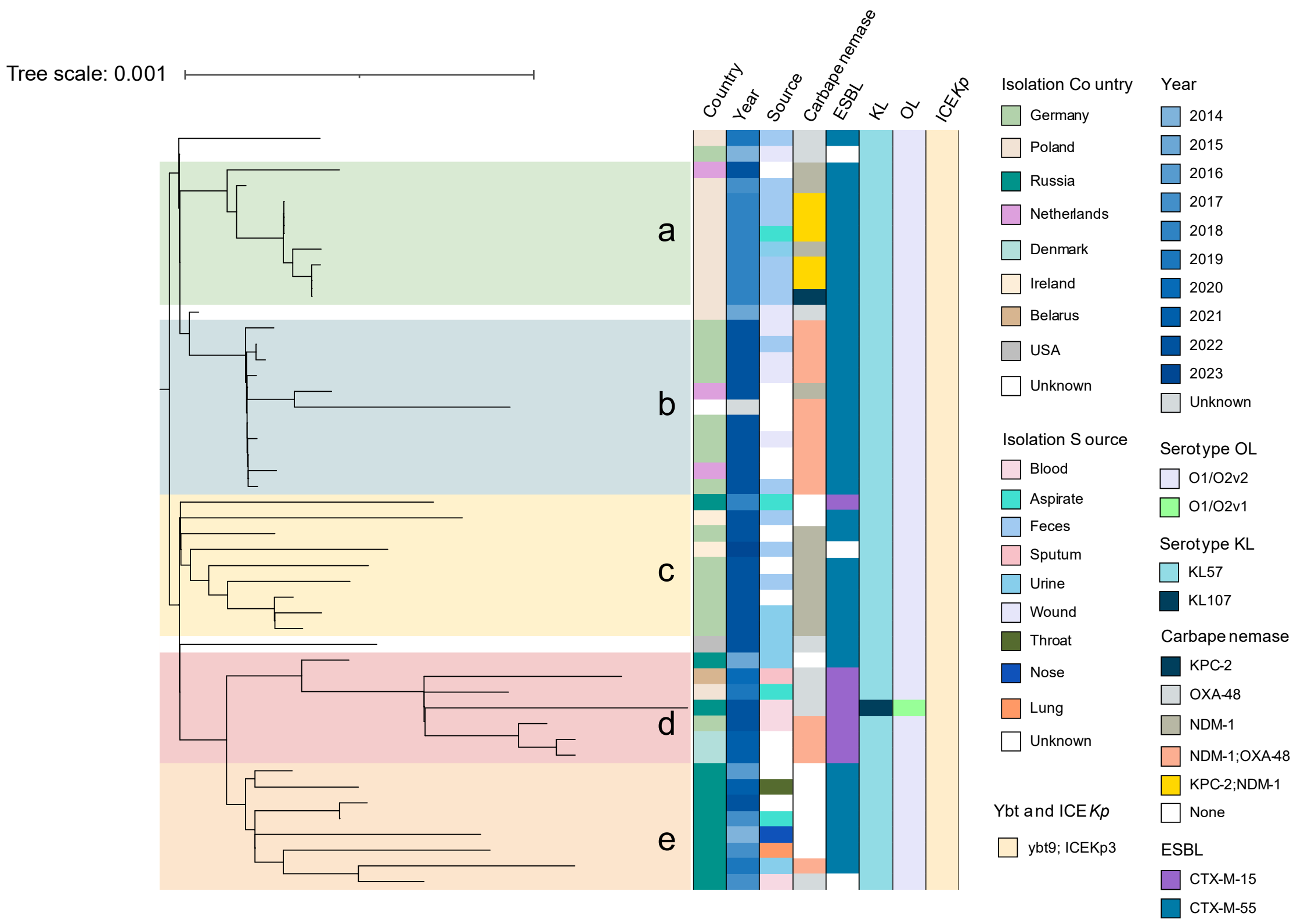

Figure S3. Maximum-likelihood phylogenetic tree of 48 *K. pneumoniae* isolates in ST23-KL57 lineage, revealing five distinct clades (Clades a-e) shown in different colors.

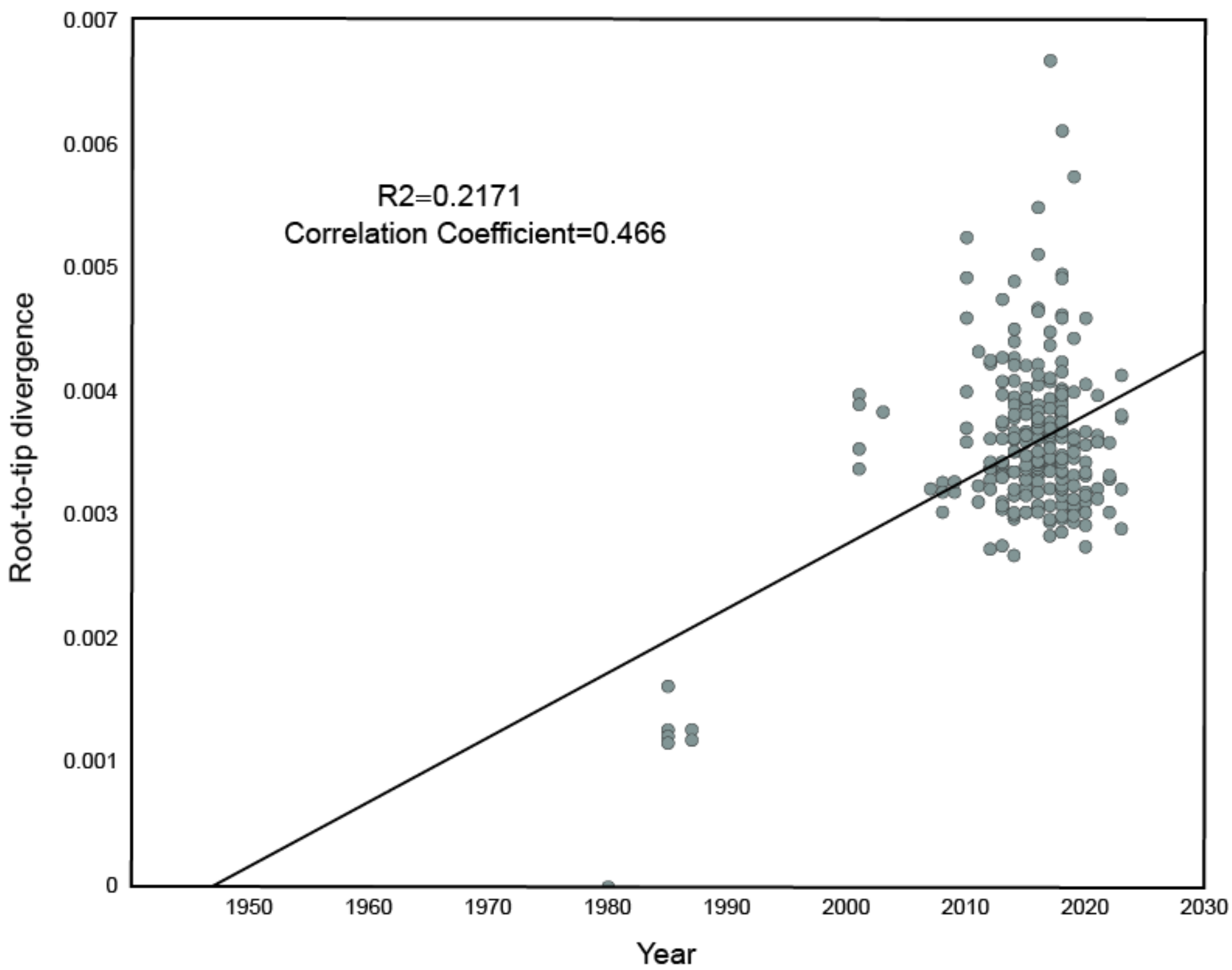

Figure S4. Temporal signal analysis of the ST23-KL1 lineage. Root-to-tip distance analysis of ST23-KL1 isolates, showing the correlation between genetic divergence and isolation year. The linear regression line indicates a significant temporal signal in the data, with isolates collected more recently showing greater genetic divergence from the root.

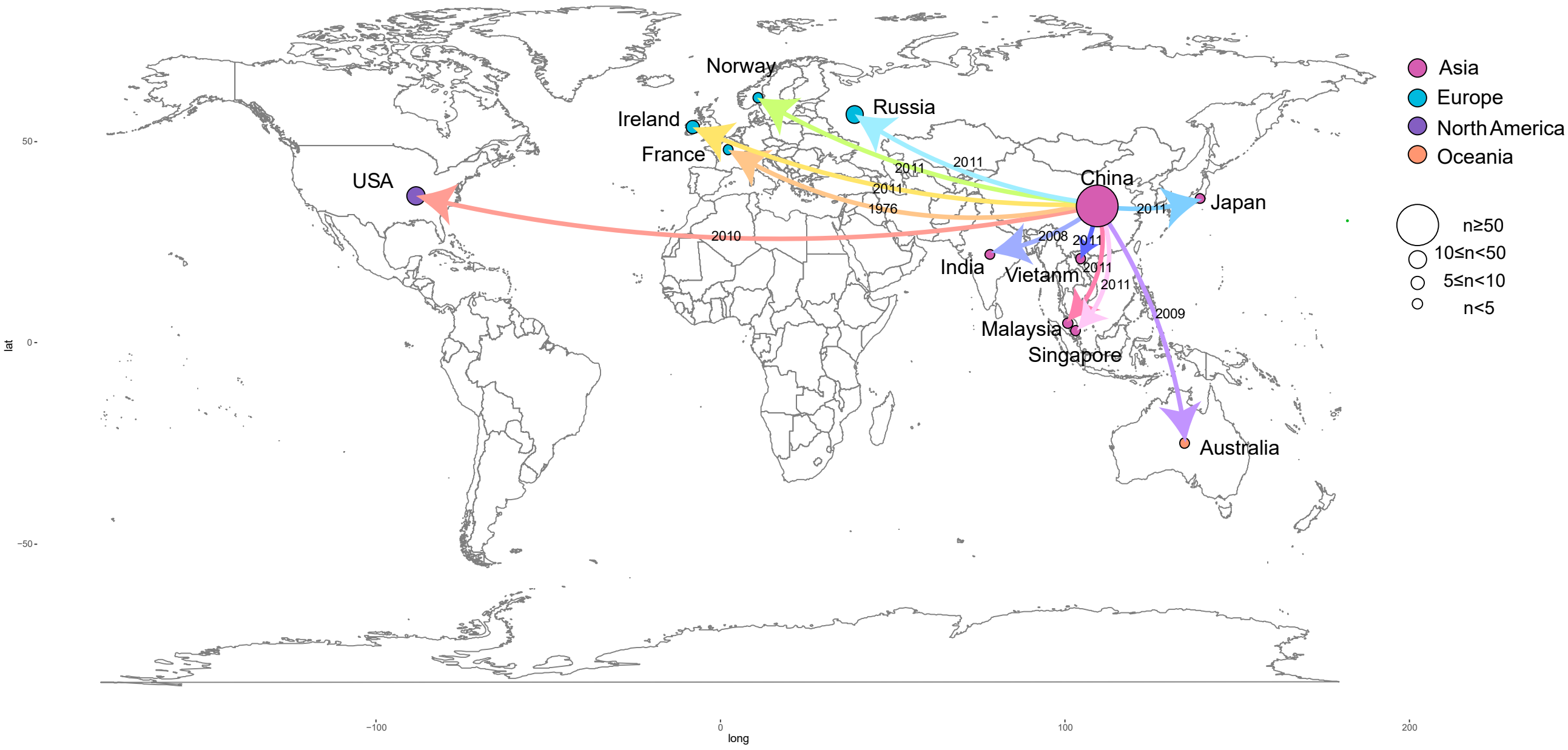

Figure S5. Phylogeographic analysis of ST23-KL1 strains from 12 major countries. Inferred phylogeographic patterns of the 255 global ST23-KL1 isolates.

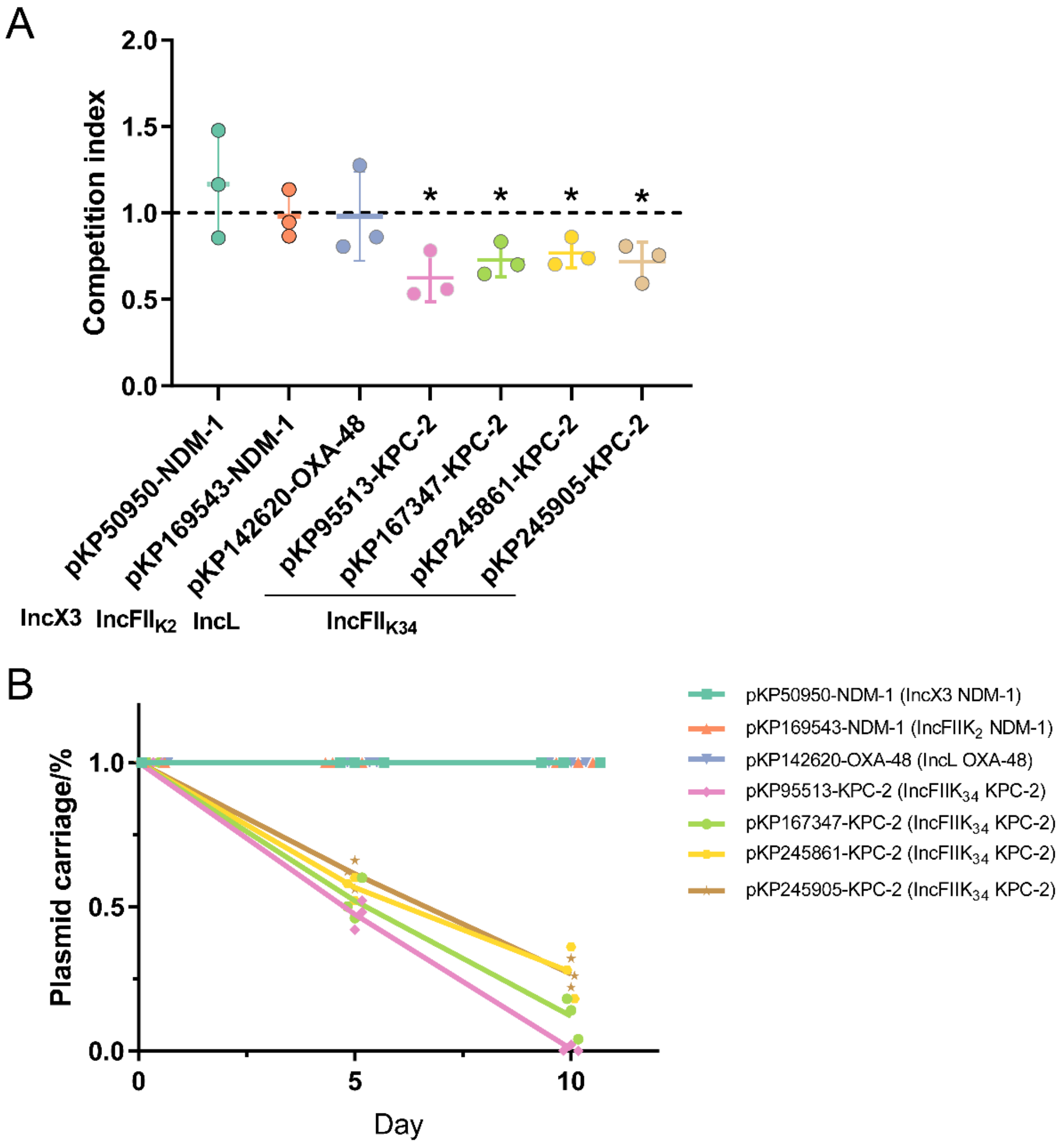

Figure S6. *In vitro* competitive ability of NTUH-K2044 transconjugants and the stability of carbapenemase-encoding plasmids. (A) Wild-type NTUH-K2044 was competed against isogenic transconjugants carrying various carbapenemase-encoding plasmids under antibiotic-free conditions (B) Plasmid stability of the NTUH-K2044 transconjugants in M9 minimal medium without antibiotics. Evolved lineages were serially passaged daily for 10 days. Every 5 days, cultures were serially diluted in phosphate-buffered saline (PBS) and plated onto Mueller–Hinton (MH) agar. Fifty randomly selected colonies from each time point were replica-plated onto MH agar supplemented with 0.1 μg/mL meropenem to determine plasmid retention. Plasmid stability was calculated as the ratio of colony-forming units (CFUs) recovered on meropenem-containing agar to those recovered on antibiotic-free agar.

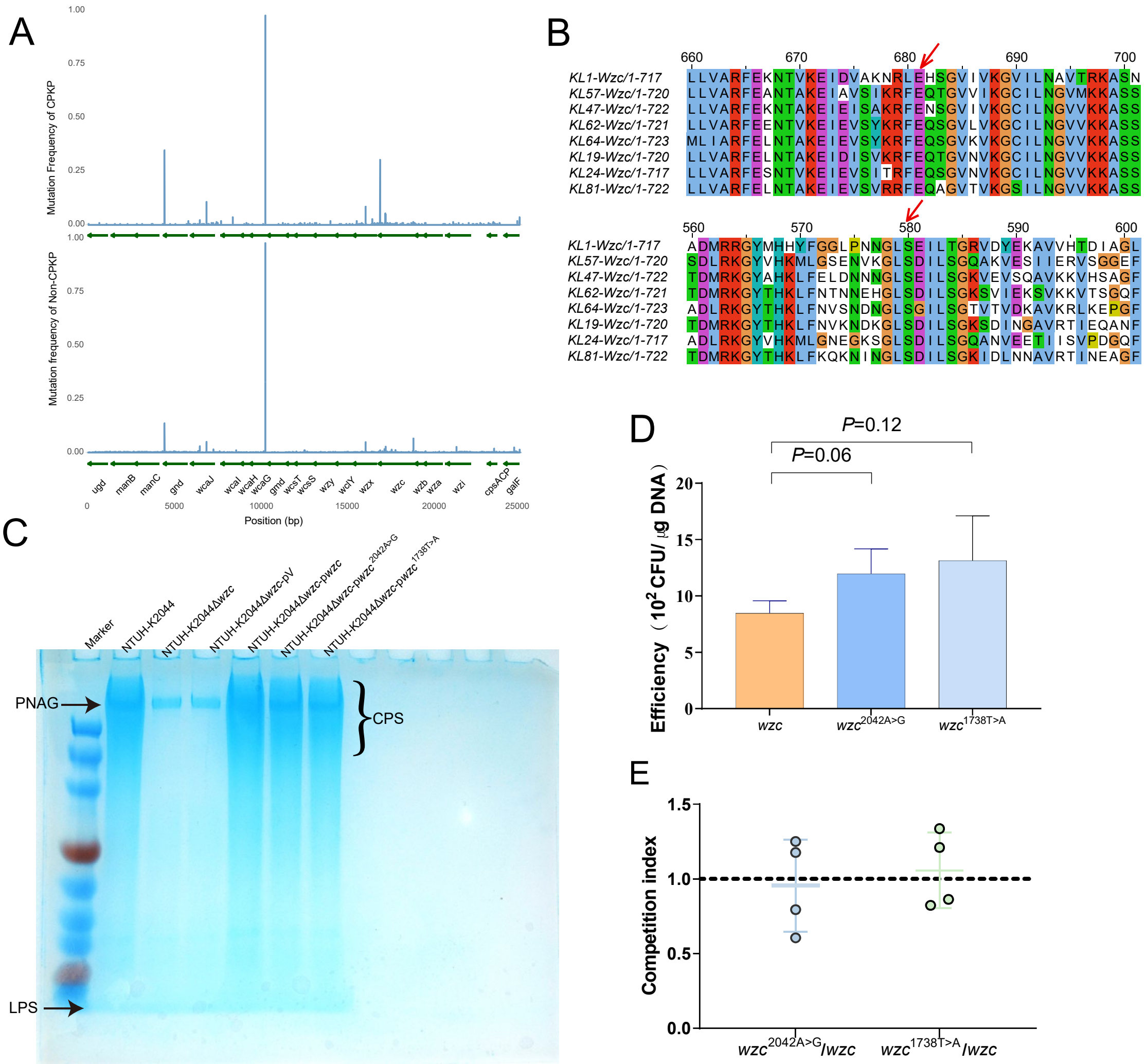

Figure S7. Genetic variation of the *cps* locus and the role of *wzc* variant in the phenotypic divergence of NTUH-K2044. (A) Distribution of single nucleotide polymorphisms (SNPs) across the *cps* locus in CP-hvKP and non-CP-hvKP strains. Green arrows indicate the positions and orientations of genes. The upper and lower panels share the same genomic position axis. (B) Multiple sequence alignment of Wzc proteins across diverse *K. pneumoniae* capsular types shows that residues E681 and S580 are evolutionarily conserved. The alignment includes 8 common KLs (KL1, KL57, KL47, KL64, KL62, KL19, KL24, and KL81). Reference sequences were obtained from the Kaptive database. (C) Quantification and visualization of capsular polysaccharide from NTUH-K2044 variants by Alcian blue-stained SDS-PAGE. (D) Electrotransformation efficiency of the KPC-2-bearing plasmid (pKP95513-KPC-2) into NTUH-K2044 expressing various *wzc* alleles. (E) *In vitro* competitive fitness of NTUH-K2044 strains complemented with mutant versus wild-type *wzc*.

Table S1. Plasmids and primers for construction of mutant and complementation strains

Plasmid/ Primers	Description/ Sequence	Use
pACBSR-*HygR*	A p15A replicon plasmid containing an arabinose-inducible λ-Red recombinase and hygromycin resistance selection marker	Homologous recombination
pCAP03-*acc(3)IV*	Template for amplification of the apramycin resistance cassette	Amplification of apramycin cassette
pFLP-*HygR*	Plasmid containing a heat-shock inducible FLP recombinase and hygromycin resistance selection marker	Excision of apramycin cassette
pBBR1-*HygR*	Cloning plasmid	Plasmid for complementation
WZCko-L-F	ACTGATTGCGTTTCCTGCATG	Amplification of *wzc* upstream for *wzc* deletion
WZCko-L-R	GGTCGACGGATCCCCGGAATATTTTAGCAGTGACCGATGC	Amplification of *wzc* upstream for *wzc* deletion
WZCkoApr-F	GCATCGGTCACTGCTAAAATATTCCGGGGATCCGTCGACC	Amplification of apramycin cassette
WZCkoApr-R	ATATGCATTTTGTAGGCTGGAGCTGCTTCG	Amplification of apramycin cassette
WZCko-R-F	CCAGCCTACAAAATGCATATAAAAAACCTAGCCCC	Amplification of *wzc* downstream for *wzc* deletion
WZCko-R-R	TTCTGCAGGACTCGGTGC	Amplification of *wzc* downstream for *wzc* deletion
WZCcom-L	AATAAACCGGTAGGATCCAGGGTCGCCTTTGAGAACAAT	Amplification of wild-type *wzc* allele for complementation
WZC681-L-R	ACCGCTATGTCCCAATCTATTTTTAGCAACATCGA	Amplification of point-mutated *wzc* variant for complementation
WZC681-R-F	ATAGATTGGGACATAGCGGTGTTATAGTTAAAGGC	Amplification of point-mutated *wzc* variant for complementation
WZCcom-R	CTTCTTAAAGTTAAACAAACACACCCAGTCCTTAAGTAAATTAAA	Amplification of wild-type *wzc* allele for complementation
WZC580-L-R	GTATCTCTGTCAAGCCATTGTTGGGCAAA	Amplification of point-mutated *wzc* variant for complementation
WZC580-R-F	CAATGGCTTGACAGAGATACTCACAGGTAGGGT	Amplification of point-mutated *wzc* variant for complementation
pbbrvec-PCR-F	CTGGATCCTACCGGTTTATTGA	Amplification of vector for complementation
pbbrvec-PCR-R	GTTTGTTTAACTTTAAGAAGGAGC	Amplification of vector for complementation

Table S2. Minimum inhibitory concentrations (MICs) of commonly used antibiotics against ten ST23 CRKP strains(μg/mL).

Isolates	TZP	CSL	CRO	CAZ	CXM	FEP	MOX	ATM	IPM	MEM	ETP	CZA	CIP	LVX	AMK	GEN	SXT	FOS	PMB	TGC
KP50950	128	128	128	128	128	64	128	128	8	4	16	> 64	0.06	0.06	2	1	0.125	4	0.25	0.25
KP95513	128	128	64	64	128	64	128	128	32	32	32	2	0.125	0.125	2	1	0.06	16	0.5	0.25
KP105791	128	128	128	128	128	32	128	64	8	4	16	> 64	0.5	0.5	1	1	4	128	0.5	0.125
KP136096	128	8	2	1	128	2	16	0.5	**0.5**	**0.25**	2	0.125	0.5	1	2	2	0.5	64	0.5	0.125
KP142620	128	64	64	32	128	32	32	128	2	4	8	0.125	32	8	4	64	8	4	0.25	0.5
KP167347	64	64	32	32	128	4	8	64	4	0.5	4	0.5	0.125	0.125	2	1	0.06	4	0.5	1
KP169509	128	128	64	64	128	64	128	64	16	4	32	32	0.06	0.06	1	64	8	4	0.5	0.125
KP169543	128	128	64	64	128	64	128	64	8	4	32	32	0.06	0.06	2	64	8	4	0.5	0.25
KP245861	>128	128	32	16	>128	4	4	32	0.5	1	4	0.25	1	0.5	2	1	0.25	1	0.5	0.125
KP245905	>128	64	16	16	>128	4	4	32	0.5	2	8	0.25	0.125	0.125	1	0.5	0.25	64	1	0.25

TZP, piperacillin/tazobactam; CSL, cefoperazone/sulbactam; CRO, ceftriaxone; CAZ, ceftazidime; CXM, cefuroxime; FEP, cefepime; MOX, moxalactam; ATM, aztreonam; IPM, imipenem; MEM, meropenem; ETP, ertapenem; CZA**,** ceftazidime/avibactam; CIP, ciprofloxacin; LVX, levofloxacin; AMK, amikacin; GEN, gentamicin; SXT, sulfamethoxazole/trimethoprim; FOS, fosfomycin; PMB, polymyxin B; TGC, tigecycline. Resistance to antibiotics is indicated by grey, while intermediate susceptibility is shown in light grey.

Table S3. Genomic characteristics of ten ST23 BSI CRKP strains.

Isolates	Total genome size (bp)	N50	Contig counts	VS	Number of virulence genes	Virulence plasmid replicons	Number of ARGs	CR plasmid replicons	Carbapenemases	Other ARGs in CR plasmid	Other replicons and ARGs	Chromosomal ARGs
KP50950	5774171	5434076	6	5	133	repB/IncHI1B(pNDM-MAR)	7	IncX3	NDM-1	*ble*、***bla***$_{\textbf{SHV-12}}$	-	*oqxB19*、*oqxA*、*bla*$_{\text{SHV-11}}$、*fosA*
KP95513	5549293	5425806	3	2	122	-	5	IncR/FII$_{\text{K34}}$	KPC-2	-	-	*oqxB19*、*oqxA*、*bla*$_{\text{SHV-11}}$、*fosA*
KP105791	5778340	5477069	3	5	133	repB/IncHI1B(pNDM-MAR)	14	IncX3	NDM-1	*ble*、***bla***$_{\textbf{CTX-M-14}}$、*fosA3*、*mph(A)*、*sul1*、*arr-3*、*catB3*、*bla*$_{\text{OXA-1}}$、*aac(6')-Ib-cr5*	-	*oqxB19*、*oqxA*、*bla*$_{\text{SHV-11}}$、*fosA*
KP136096	5687787	5459869	2	5	133	repB/IncHI1B(pNDM-MAR)	4	-	-	-	-	*oqxB19*、*oqxA*、*bla*$_{\text{SHV-11}}$、*fosA*
KP142620	5846983	5419603	4	5	133	repB/IncHI1B(pNDM-MAR)	16	IncL	OXA-48	-	IncR/FII$_{\text{K7}}$: *dfrA14*、*qnrB1*、*sul2*、*aph(3'')-Ib*、*aph(6)-Id*、*bla*$_{\text{TEM-1}}$、***bla***$_{\textbf{CTX-M-15}}$、*bla*$_{\text{OXA-1}}$、*aac(6')-Ib-cr5*、*aac(3)-IIe*、*tet(A)*	*oqxB19*、*oqxA*、*bla*$_{\text{SHV-11}}$、*fosA*
KP167347	5926752	5486369	4	5	133	repB/IncHI1B(pNDM-MAR)	5	IncR/FII$_{\text{K34}}$	KPC-2	-	-	*oqxB19*、*oqxA*、*bla*$_{\text{SHV-11}}$、*fosA*
KP169509	5817809	5449257	4	5	133	repB/IncHI1B(pNDM-MAR)	14	repB(R1701)/FII$_{\text{K2}}$、IncX3	NDM-1	repB(R1701)/FII$_{\text{K2}}$: *ble*、*sul1*、*aadA2*、*dfrA12*、*aac(3)-IId*、*bla*$_{\text{TEM-1}}$、***bla***$_{\textbf{CTX-M-15}}$ IncX3: *ble*、***bla***$_{\textbf{SHV-12}}$	-	*oqxB19*、*oqxA*、*bla*$_{\text{SHV-11}}$、*fosA*
KP169543	5762860	5448057	3	5	133	repB/IncHI1B(pNDM-MAR)	12	repB(R1701)/FII$_{\text{K2}}$	NDM-1	*ble*、*sul1*、*aadA2*、*dfrA12*、*aac(3)-IId*、*bla*$_{\text{TEM-1}}$、***bla***$_{\textbf{CTX-M-15}}$	-	*oqxB19*、*oqxA*、*bla*$_{\text{SHV-11}}$、*fosA*
KP245861	5732797	5401458	3	5	133	repB/IncHI1B(pNDM-MAR)	6	repB(R1701)/FII$_{\text{K34}}$	KPC-2	*qnrS1*	-	*oqxB19*、*oqxA*、*bla*$_{\text{SHV-11}}$、*fosA*
KP245905	5793281	5459132	3	5	133	repB/IncHI1B(pNDM-MAR)	5	repB(R1701)/FII$_{\text{K34}}$	KPC-2	-	-	*oqxB19*、*oqxA*、*bla*$_{\text{SHV-11}}$、*fosA*

Average Illumina depth of coverage was 167X, and average ONT depth of coverage was 80X. VS, Virulence score; CR, carbapenem-resistant; ARGs, antibiotic resistance genes. Extended-spectrum β-lactamase (ESBL) genes were bold

Table S4. Metadata and genotype of all ST23 genomes

Accession	ST	K_locus	O_locus	Country	Year	source	Num ARGs	Num VGs	CPKP	ESBL
SAMEA6375547	ST23	KL1	O1/O2v2	Colombia	2016	Urine	8	120	KPC-2	-
SAMEA4531948	ST23	KL1	O1/O2v2	Philippines	2014	Aspirate	16	131	-	CTX-M-15
SAMEA4531929	ST23	KL1	O1/O2v2	Philippines	2013	Sputum	14	129	-	CTX-M-15
SAMEA4531999	ST23	KL1	O1/O2v2	Philippines	2014	Aspirate	23	130	-	CTX-M-15
SAMN12392859	ST23	KL1	O1/O2v2	USA	2013	Blood	4	128	-	-
SAMN06112175	ST23	KL1	O1/O2v2	Singapore	2014	Liver	4	125	-	-
SAMN11878537	ST23	KL1	O1/O2v2	Russia	2013	Unknown	14	124	-	CTX-M-15
SAMN07256676	ST23	KL1	O1/O2v2	France	1985	Animal	9	126	-	-
SAMN07256686	ST23	KL1	O1/O2v2	Spain	1997	Blood	4	128	-	-
SAMN07256674	ST23	KL1	O1/O2v2	Netherlands	1997	Animal	4	128	-	-
SAMEA29232418	ST23	KL1	O1/O2v2	Germany	2012	Aspirate	16	128	OXA-48	CTX-M-15
SAMN12250621	ST23	KL1	O1/O2v1	USA	2015	Lung	4	123	-	-
SAMN11878533	ST23	KL1	O1/O2v2	Russia	2014	Urine	16	106	OXA-48	CTX-M-15
SAMN07256685	ST23	KL1	O1/O2v2	France	1987	Animal	8	123	-	-
SAMN12250622	ST23	KL1	O1/O2v2	USA	2015	Lung	4	123	-	-
SAMN11878532	ST23	KL1	O1/O2v2	Russia	2014	Urine	16	124	OXA-48	CTX-M-15
SAMN07256679	ST23	KL1	O1/O2v2	France	1985	Animal	8	129	-	-
SAMN11878529	ST23	KL1	O1/O2v2	Russia	2013	Aspirate	21	118	OXA-48	CTX-M-15
SAMN06606879	ST23	KL1	O1/O2v2	Russia	2003	Feces	4	118	-	-
SAMEA104414832	ST23	KL1	O1/O2v2	Italy	2016	Unknown	8	128	VIM-1	-
SAMN07311085	ST23	KL1	O1/O2v1	USA	2015	Unknown	4	100	-	-
SAMN07311089	ST23	KL1	O1/O2v1	USA	2015	Unknown	4	100	-	-
SAMN07311090	ST23	KL1	O1/O2v1	USA	2015	Unknown	4	100	-	-
SAMN07256678	ST23	KL1	O1/O2v2	France	1985	Animal	7	118	-	-
SAMN07311091	ST23	KL1	O1/O2v1	USA	2015	Unknown	4	100	-	-
SAMN07256682	ST23	KL1	O1/O2v2	France	1987	Animal	8	125	-	-
SAMN06112181	ST23	KL1	O1/O2v2	Singapore	2014	Liver	4	119	-	-
SAMN07256673	ST23	KL1	O1/O2v2	France	1985	Animal	5	129	-	-
SAMN11878536	ST23	KL1	O1/O2v2	Russia	2014	Wound	16	111	OXA-48	CTX-M-15
SAMN12349650	ST23	KL1	O1/O2v2	Russia	2017	Feces	16	128	-	CTX-M-15
SAMN06112132	ST23	KL1	O1/O2v2	Australia	Unknown	Lung	4	100	-	-
SAMN12349652	ST23	KL1	O1/O2v2	Russia	2017	Feces	4	128	-	-
SAMN11979485	ST23	KL1	O1/O2v2	Singapore	2014	Blood	8	123	KPC-2	-
SAMN12349653	ST23	KL1	O1/O2v2	Russia	2017	Feces	16	128	-	CTX-M-15
SAMN12349654	ST23	KL1	O1/O2v2	Russia	2017	Feces	16	128	-	CTX-M-15

SAMN12419069	ST23	KL1	O1/O2v2	Singapore	2014	Blood	8	123	KPC-2	-
SAMN12349649	ST23	KL1	O1/O2v2	Russia	2017	Feces	16	128	-	CTX-M-15
SAMEA4668426	ST23	KL1	O1/O2v2	Vietnam	2014	Blood	4	126	-	-
SAMN10763117	ST23	KL1	O1/O2v2	Czech Republic	2014	Blood	14	128	-	CTX-M-15
SAMN15541470	ST23	KL1	O1/O2v2	Vietnam	Unknown	Nose	4	125	-	-
SAMEA4362688	ST23	KL1	O1/O2v2	United Kingdom	2015	Environment	5	124	-	-
SAMEA6531483	ST23	KL1	O1/O2v2	Saudi Arabia	2018	Unknown	4	128	-	-
SAMN15541514	ST23	KL1	O1/O2v2	Vietnam	Unknown	Nose	4	127	-	-
SAMN08957370	ST23	KL1	O1/O2v2	China	2016	Unknown	4	128	-	-
SAMN05770794	ST23	KL1	O1/O2v2	Singapore	2014	Unknown	10	127	KPC-2	-
SAMEA3531823	ST23	KL1	O1/O2v2	Thailand	2015	Blood	6	129	-	CTX-M-55
SAMN05581771	ST23	KL1	O1/O2v2	Denmark	Unknown	Unknown	4	128	-	-
SAMEA6531362	ST23	KL1	O1/O2v2	Saudi Arabia	2018	Unknown	4	128	-	-
SAMEA4362686	ST23	KL1	O1/O2v2	United Kingdom	2015	Environment	5	125	-	-
SAMN06140912	ST23	KL57	O1/O2v2	Russia	2016	Unknown	17	85	-	CTX-M-55
SAMN06112186	ST23	KL1	O1/O2v2	Singapore	2014	Liver	4	125	-	-
SAMN06112172	ST23	KL1	O1/O2v2	Singapore	2014	Liver	4	126	-	-
SAMEA4362679	ST23	KL1	O1/O2v2	United Kingdom	2015	Environment	5	124	-	-
SAMN07311086	ST23	KL1	O1/O2v1	USA	2015	Unknown	4	100	-	-
SAMN10763118	ST23	KL1	O1/O2v2	Czech Republic	2014	Feces	14	128	-	CTX-M-15
SAMEA3499900	ST23	KL1	O1/O2v2	Austria	2014	Wound	4	128	-	-
SAMEA4668463	ST23	KL1	O1/O2v2	Vietnam	2012	Blood	4	101	-	-
SAMEA4364606	ST23	KL1	O1/O2v2	Thailand	2016	Unknown	5	128	OXA-232	-
SAMN03853196	ST23	KL1	O1/O2v2	USA	2012	Urine	4	129	-	-
SAMN11878538	ST23	KL1	O1/O2v2	Russia	2016	Unknown	4	128	-	-
SAMEA4668407	ST23	KL1	O1/O2v2	Vietnam	2011	Blood	4	121	-	-
SAMN15541467	ST23	KL1	O1/O2v2	Vietnam	Unknown	Nose	4	126	-	-
SAMEA4668455	ST23	KL1	O1/O2v2	Vietnam	2013	Blood	5	127	-	-
SAMEA3729824	ST23	KL1	O1/O2v2	Turkey	2013	Urine	5	127	OXA-48	-
SAMN15541423	ST23	KL1	O1/O2v2	Vietnam	Unknown	Lung	4	120	-	-
SAMN06112193	ST23	KL1	O1/O2v2	Singapore	Unknown	Liver	4	115	-	-
SAMEA4668445	ST23	KL1	O1/O2v2	Vietnam	2010	Blood	4	126	-	-
SAMEA4364639	ST23	KL1	O1/O2v2	Thailand	2016	Unknown	5	128	-	-
SAMN07256675	ST23	KL1	O1/O2v2	France	1980	Animal	9	130	-	-
SAMN12419070	ST23	KL1	O1/O2v2	Singapore	2014	Feces	8	128	KPC-2	-
SAMEA4668404	ST23	KL1	O1/O2v2	Vietnam	2011	Blood	4	112	-	-

SAMEA4362681	ST23	KL1	O1/O2v2	United Kingdom	2015	Environment	5	125	-	-
SAMN06112168	ST23	KL1	O1/O2v2	Singapore	2013	Liver	4	118	-	-
SAMN06112178	ST23	KL1	O1/O2v2	Singapore	2014	Liver	4	100	-	-
SAMEA4362689	ST23	KL1	O1/O2v2	United Kingdom	2015	Environment	5	124	-	-
SAMEA4364579	ST23	KL1	O1/O2v2	Thailand	2016	Unknown	6	100	OXA-232	-
SAMN08957373	ST23	KL1	O1/O2v2	China	2013	Unknown	4	128	-	-
SAMN11979488	ST23	KL1	O1/O2v2	Singapore	2014	Feces	8	128	KPC-2	-
SAMN06112135	ST23	KL1	O1/O2v2	Australia	2013	Blood	4	100	-	-
SAMEA4362690	ST23	KL1	O1/O2v2	United Kingdom	2015	Environment	5	125	-	-
SAMN15541427	ST23	KL1	O1/O2v2	Vietnam	Unknown	Lung	4	128	-	-
SAMEA3515178	ST23	KL1	O1/O2v2	Italy	2014	Environment	4	130	-	-
SAMN06112169	ST23	KL1	O1/O2v2	Singapore	2013	Liver	4	124	-	-
SAMEA4364601	ST23	KL1	O1/O2v2	Thailand	2016	Unknown	5	112	OXA-232	-
SAMN11878531	ST23	KL1	O1/O2v2	Russia	2015	Unknown	4	127	-	-
SAMN15541513	ST23	KL1	O1/O2v2	Vietnam	Unknown	Nose	4	128	-	-
SAMEA4668390	ST23	KL1	O1/O2v2	Vietnam	2011	Blood	4	127	-	-
SAMN06112176	ST23	KL1	O1/O2v2	Singapore	2014	Liver	4	126	-	-
SAMEA4668467	ST23	KL1	O1/O2v2	Vietnam	2012	Blood	4	127	-	-
SAMN06112179	ST23	KL1	O1/O2v2	Singapore	2014	Liver	4	111	-	-
SAMEA3499911	ST23	KL1	O1/O2v2	Estonia	2013	Aspirate	4	101	-	-
SAMEA4668429	ST23	KL1	O1/O2v2	Vietnam	2014	Blood	4	100	-	-
SAMN08957365	ST23	KL1	O1/O2v2	China	2012	Unknown	4	128	-	-
SAMN15541422	ST23	KL1	O1/O2v2	Vietnam	Unknown	Lung	4	125	-	-
SAMN07609107	ST23	KL1	O1/O2v2	China	2016	Unknown	4	130	-	-
SAMEA4364581	ST23	KL1	O1/O2v2	Thailand	2016	Unknown	4	128	-	-
SAMEA4362682	ST23	KL1	O1/O2v2	United Kingdom	2015	Environment	5	124	-	-
SAMN14792099	ST23	KL1	O1/O2v2	USA	2020	Blood	4	128	-	-
SAMN11878530	ST23	KL1	O1/O2v2	Russia	2014	Unknown	4	128	-	-
SAMN05581754	ST23	KL1	O1/O2v2	USA	2012	Abscess	4	128	-	-
SAMN07609171	ST23	KL1	O1/O2v2	China	2017	Unknown	4	128	-	-
SAMEA5232581	ST23	KL1	O1/O2v2	India	2017	Blood	4	94	-	-
SAMN06190126	ST23	KL1	O1/O2v2	Czech Republic	2014	Wound	17	128	-	CTX-M-15
SAMN11979487	ST23	KL1	O1/O2v2	Singapore	2014	Feces	10	127	KPC-2	-
SAMN06112177	ST23	KL1	O1/O2v2	Singapore	2014	Liver	4	124	-	-
SAMEA1712950	ST23	KL1	O1/O2v2	United Kingdom	2008	Unknown	4	128	-	-
SAMN11878534	ST23	KL1	O1/O2v2	Russia	2013	Aspirate	4	128	-	-

SAMN08624691	ST23	KL1	O1/O2v2	China	2016	Unknown	9	129	NDM-1	CTX-M-15
SAMEA3531861	ST23	KL1	O1/O2v2	Thailand	2015	Urine	18	129	-	CTX-M-15
SAMEA4364603	ST23	KL1	O1/O2v2	Thailand	2016	Unknown	16	129	OXA-232	-
SAMN07256683	ST23	KL1	O1/O2v2	France	1987	Animal	4	128	-	-
SAMN11979486	ST23	KL1	O1/O2v2	Singapore	2013	Sputum	7	128	KPC-2	-
SAMEA4362684	ST23	KL1	O1/O2v2	United Kingdom	2015	Environment	5	125	-	-
SAMN15541431	ST23	KL1	O1/O2v2	Vietnam	Unknown	Blood	4	127	-	-
SAMEA3729828	ST23	KL1	O1/O2v2	Turkey	2013	Aspirate	4	127	-	-
SAMEA4362687	ST23	KL1	O1/O2v2	United Kingdom	2015	Environment	5	125	-	-
SAMN12419067	ST23	KL1	O1/O2v2	Singapore	2013	Blood	12	128	KPC-2	-
SAMN06112143	ST23	KL1	O1/O2v2	Australia	2010	Blood	4	101	-	-
SAMEA4668398	ST23	KL1	O1/O2v2	Vietnam	2012	Blood	4	128	-	-
SAMN12419068	ST23	KL1	O1/O2v2	Singapore	2014	Feces	10	127	KPC-2	-
SAMN07508954	ST23	KL1	O1/O2v2	China	Unknown	Unknown	6	130	KPC-2	-
SAMEA4364645	ST23	KL1	O1/O2v2	Thailand	2016	Unknown	5	128	OXA-232	-
SAMN12419066	ST23	KL1	O1/O2v2	Singapore	2013	Sputum	7	128	KPC-2	-
SAMN12003074	ST23	KL1	O1/O2v2	India	2016	Blood	24	110	OXA-232	CTX-M-15
SAMEA23989918	ST23	KL1	O1/O2v2	Thailand	2016	Unknown	4	128	-	-
SAMEA4364640	ST23	KL1	O1/O2v2	Thailand	2016	Unknown	6	127	-	-
SAMN11878535	ST23	KL1	O1/O2v2	Russia	2012	Sputum	16	125	OXA-48	CTX-M-15
SAMEA4362683	ST23	KL1	O1/O2v2	United Kingdom	2015	Environment	14	125	-	-
SAMN08957364	ST23	KL1	O1/O2v2	China	2012	Unknown	4	128	-	-
SAMN06112185	ST23	KL1	O1/O2v2	Singapore	2014	Liver	4	123	-	-
SAMEA4362685	ST23	KL1	O1/O2v2	United Kingdom	2015	Environment	14	125	-	-
SAMN11054834	ST23	KL1	O1/O2v2	USA	2016	Urine	8	127	KPC-2	-
SAMN07256680	ST23	KL1	O1/O2v2	France	1985	Animal	7	127	-	-
SAMN11125719	ST23	KL1	O1/O2v2	France	2008	Unknown	4	124	-	-
SAMN06112188	ST23	KL1	O1/O2v2	Singapore	2015	Liver	4	125	-	-
SAMN15541425	ST23	KL1	O1/O2v2	Vietnam	Unknown	Lung	4	125	-	-
SAMEA4668391	ST23	KL1	O1/O2v2	Vietnam	2011	Blood	4	121	-	-
SAMN15541468	ST23	KL1	O1/O2v2	Vietnam	Unknown	Nose	4	124	-	-
SAMN13166969	ST23	KL1	O1/O2v2	USA	2016	Urine	8	127	KPC-2	-
SAMEA5232487	ST23	KL1	O1/O2v2	Vietnam	2015	Blood	4	127	-	-
SAMN12250733	ST23	KL1	O1/O2v2	USA	2015	Lung	4	126	-	-
SAMN07256672	ST23	KL1	O1/O2v2	Belgium	1997	Blood	4	118	-	-
SAMN08707458	ST23	KL1	O1/O2v2	China	2017	Unknown	16	129	-	CTX-M-3

SAMEA104208361	ST23	KL1	O1/O2v2	Laos	2016	Blood	4	128	-	-
SAMEA882041	ST23	KL1	O1/O2v2	Australia	2002	Unknown	4	128	-	-
SAMEA104208339	ST23	KL1	O1/O2v2	Cambodia	2016	Blood	4	128	-	-
SAMEA104208394	ST23	KL1	O1/O2v2	Laos	2016	Blood	4	128	-	-
SAMEA104208376	ST23	KL1	O1/O2v2	Laos	2015	Blood	4	128	-	-
SAMEA104208404	ST23	KL1	O1/O2v2	Laos	2016	Blood	9	128	-	CTX-M-63
SAMEA104208402	ST23	KL1	O1/O2v2	Laos	2016	Blood	4	128	-	-
SAMEA104208386	ST23	KL1	O1/O2v2	Laos	2015	Blood	4	128	-	-
SAMEA104208354	ST23	KL1	O1/O2v2	Laos	2015	Blood	4	128	-	-
SAMEA104208429	ST23	KL1	O1/O2v2	Hong Kong	2016	Blood	4	128	-	-
SAMEA7190509	ST23	KL1	O1/O2v2	Germany	Unknown	Unknown	11	123	-	-
SAMEA7190475	ST23	KL1	O1/O2v2	Germany	Unknown	Unknown	4	127	-	-
SAMEA7077202	ST23	KL1	O1/O2v2	Thailand	2018	Unknown	4	101	-	-
SAMEA7190506	ST23	KL1	O1/O2v2	Germany	Unknown	Unknown	4	126	-	-
SAMEA7701935	ST23	KL1	O1/O2v2	Spain	Unknown	Unknown	4	129	-	-
SAMEA7701922	ST23	KL1	O1/O2v2	Spain	Unknown	Unknown	8	121	-	-
SAMEA7773985	ST23	KL1	O1/O2v2	Norway	2015	Feces	5	119	-	-
SAMEA8948777	ST23	KL1	O1/O2v2	Norway	2015	Blood	4	124	-	-
SAMEA8919792	ST23	KL1	O1/O2v1	Germany	2007	Food	4	126	-	-
SAMEA8664744	ST23	KL1	O1/O2v2	Sweden	2018	Blood	4	128	-	-
SAMEA8948285	ST23	KL1	O1/O2v2	Norway	2001	Blood	4	124	-	-
SAMEA8948357	ST23	KL1	O1/O2v2	Norway	2001	Blood	9	127	-	-
SAMEA8948606	ST23	KL1	O1/O2v2	Norway	2009	Blood	4	128	-	-
SAMEA8948437	ST23	KL1	O1/O2v2	Norway	2009	Blood	4	128	-	-
SAMEA8948473	ST23	KL1	O1/O2v2	Norway	2009	Blood	4	127	-	-
SAMN18977290	ST23	KL1	O1/O2v2	USA	2016	Feces	14	128	KPC-3	-
SAMN18679915	ST23	KL57	O1/O2v2	Russia	2018	aspirate	18	86	-	CTX-M-15
SAMN17807608	ST23	KL1	O1/O2v2	Russia	2019	Feces	4	125	-	-
SAMN16357454	ST23	KL1	O1/O2v2	USA	Unknown	Unknown	4	128	-	-
SAMN17807609	ST23	KL1	O1/O2v2	Russia	2019	Feces	4	126	-	-
SAMN18108799	ST23	KL1	O1/O2v2	USA	2017	Unknown	5	110	-	-
SAMN17846707	ST23	KL1	O1/O2v2	Australia	2020	Unknown	4	128	-	-
SAMN17846708	ST23	KL1	O1/O2v2	Australia	2020	Unknown	4	128	-	-
SAMEA8997930	ST23	KL1	O1/O2v2	South Africa	2017	Unknown	4	128	-	-
SAMN18898210	ST23	KL57	O1/O2v2	Russia	2019	Urine	27	83	NDM-1;OXA-48	CTX-M-55
SAMN17846705	ST23	KL1	O1/O2v2	Australia	2020	Unknown	4	128	-	-

SAMN19016284	ST23	KL1	O1/O2v2	China	2017	Sputum	6	128	KPC-2	-
SAMN15869010	ST23	KL1	O1/O2v2	USA	2016	Sputum	12	128	KPC-2	-
SAMN19016118	ST23	KL1	O1/O2v2	China	2018	Sputum	15	127	KPC-2	CTX-M-3
SAMN15869164	ST23	KL1	O1/O2v2	USA	2016	Urine	7	125	KPC-2	-
SAMN19015818	ST23	KL1	O1/O2v2	Singapore	2018	Abdomen fluid	14	123	-	CTX-M-3
SAMN19015898	ST23	KL1	O1/O2v2	China	2017	Sputum	5	128	KPC-2	-
SAMN18720461	ST23	KL1	O1/O2v2	Australia	2018	Leg	5	100	-	-
SAMN20033625	ST23	KL1	O1/O2v2	Australia	2019	Unknown	5	126	KPC-2	-
SAMN19658556	ST23	KL1	O1/O2v2	Canada	2017	Sputum	4	127	-	-
SAMN20338552	ST23	KL1	O1/O2v1	Russia	2020	Lung	4	114	-	-
SAMN21380261	ST23	KL1	O1/O2v2	China	2019	sputum	4	128	-	-
SAMN07436583	ST23	KL1	O1/O2v2	China	2013	sputum	4	128	-	-
SAMN12886385	ST23	KL1	O1/O2v2	China	2019	Unknown	5	128	-	-
SAMN12886388	ST23	KL1	O1/O2v2	China	2019	Unknown	4	128	-	-
SAMN07436585	ST23	KL1	O1/O2v2	China	2013	sputum	9	128	-	CTX-M-14
SAMN12886386	ST23	KL1	O1/O2v2	China	2019	Unknown	4	128	-	-
SAMN12886382	ST23	KL1	O1/O2v2	China	2019	Unknown	4	128	-	-
SAMN07436570	ST23	KL1	O1/O2v2	China	2013	sputum	4	101	-	-
SAMN12886383	ST23	KL1	O1/O2v2	China	2019	Unknown	4	128	-	-
SAMN12886381	ST23	KL1	O1/O2v2	China	2019	Unknown	4	123	-	-
SAMN07436579	ST23	KL1	O1/O2v2	China	2013	sputum	9	128	-	-
SAMN12886387	ST23	KL1	O1/O2v2	China	2019	Unknown	9	128	-	-
SAMN12886389	ST23	KL1	O1/O2v2	China	2019	Unknown	4	128	-	-
SAMN12886391	ST23	KL1	O1/O2v2	China	2019	Unknown	9	128	-	CTX-M-14
SAMN07436550	ST23	KL1	O1/O2v2	China	2009	sputum	4	123	-	-
SAMN12886390	ST23	KL1	O1/O2v2	China	2019	Unknown	9	128	-	CTX-M-14
SAMN07436572	ST23	KL1	O1/O2v2	China	2013	sputum	4	128	-	-
SAMN07436567	ST23	KL1	O1/O2v2	China	2013	sputum	9	128	-	CTX-M-14
SAMN16378183	ST23	KL5	O3/O3a	Russia	2014	Urine	16	103	OXA-48	CTX-M-15
SAMN16378182	ST23	KL1	O1/O2v2	Russia	2013	aspirate	4	127	-	-
SAMN16558209	ST23	KL1	O1/O2v2	USA	2017	Urine	13	128	-	-
SAMN16378174	ST23	KL57	O1/O2v2	Russia	2015	Urine	17	86	-	CTX-M-55
SAMN16378184	ST23	KL1	O1/O2v2	Russia	2014	Urine	16	124	OXA-48	CTX-M-15
SAMN16378185	ST23	KL1	O1/O2v2	Russia	2012	sputum	16	120	OXA-48	CTX-M-15
SAMN16579468	ST23	KL1	O1/O2v2	Algeria	2011	Wound	6	128	-	CTX-M-15
SAMN15904708	ST23	KL1	O1/O2v2	Hong Kong	2016	Feces	17	118	NDM-5	CTX-M-3
SAMN16378176	ST23	KL1	O1/O2v2	Russia	2015	Throat	4	127	-	-

SAMN16822476	ST23	KL1	O1/O2v2	USA	2020	Blood	4	128	-	-
SAMN16378187	ST23	KL107	O1/O2v2	Russia	2016	Lung	21	110	OXA-48	CTX-M-15
SAMN16378175	ST23	KL1	O1/O2v2	Russia	2014	Throat	4	128	-	-
SAMN16226490	ST23	KL1	O1/O2v2	USA	2014	Animal	4	112	-	-
SAMN16378172	ST23	KL57	O1/O2v2	Russia	2014	Nose	17	84	-	CTX-M-55
SAMEA3180855	ST23	KL1	O1/O2v2	Taiwan	1996	Liver	4	127	-	-
SAMN17807611	ST23	KL1	O1/O2v2	Russia	2019	Throat	4	125	-	-
SAMEA3180854	ST23	KL1	O1/O2v2	Taiwan	1996	Liver	4	128	-	-
SAMEA3182015	ST23	KL1	O1/O2v2	South Africa	1997	Lung	4	129	-	-
SAMEA2633709	ST23	KL1	O1/O2v2	France	Unknown	Blood	4	123	-	-
SAMEA3180851	ST23	KL1	O1/O2v2	Taiwan	1996	Liver	4	128	-	-
SAMEA3182012	ST23	KL1	O1/O2v2	Taiwan	1997	Liver	4	128	-	-
SAMEA3172287	ST23	KL1	O1/O2v2	USA	2005	Liver	4	128	-	-
SAMEA2633714	ST23	KL1	O1/O2v2	France	2010	Blood	4	100	-	-
SAMEA3182708	ST23	KL1	O1/O2v2	Canada	2005	Liver	4	128	-	-
SAMEA3180385	ST23	KL1	O1/O2v2	USA	2007	Liver	4	128	-	-
SAMEA3172315	ST23	KL1	O1/O2v2	USA	2005	Liver	4	128	-	-
SAMEA3178291	ST23	KL1	O1/O2v2	Canada	2006	Liver	4	89	-	-
SAMEA3182013	ST23	KL1	O1/O2v2	South Africa	1996	Lung	4	128	-	-
SAMEA3180382	ST23	KL1	O1/O2v2	Canada	2006	Liver	4	127	-	-
SAMEA3182897	ST23	KL1	O1/O2v2	Norway	2008	Liver	4	123	-	-
SAMEA3180849	ST23	KL1	O1/O2v2	Norway	2010	Liver	4	100	-	-
SAMEA3182011	ST23	KL1	O1/O2v2	Taiwan	1996	Liver	4	127	-	-
SAMEA2637224	ST23	KL1	O1/O2v2	Taiwan	Unknown	Liver	4	123	-	-
SAMEA3182898	ST23	KL1	O1/O2v2	Denmark	2008	Liver	11	127	-	-
SAMEA3180850	ST23	KL1	O1/O2v2	Norway	2010	Liver	4	101	-	-
SAMEA3182705	ST23	KL1	O1/O2v2	USA	2004	Liver	4	129	-	-
SAMEA3182895	ST23	KL1	O1/O2v2	USA	2006	Liver	4	128	-	-
SAMEA3180853	ST23	KL1	O1/O2v2	Taiwan	1996	Liver	4	128	-	-
SAMEA3182706	ST23	KL1	O1/O2v2	Canada	2005	Liver	4	128	-	-
SAMEA3180383	ST23	KL1	O1/O2v2	Canada	2006	Liver	4	130	-	-
SAMEA2637223	ST23	KL1	O1/O2v2	Taiwan	Unknown	Liver	4	109	-	-
SAMEA3182906	ST23	KL1	O1/O2v2	Norway	2012	Liver	4	128	-	-
SAMEA2637231	ST23	KL1	O1/O2v2	Netherlands	1997	Blood	4	128	-	-
SAMEA3180384	ST23	KL1	O1/O2v2	USA	2007	Liver	4	128	-	-
SAMD00212274	ST23	KL1	O1/O2v2	Japan	2015	Unknown	4	128	-	-
SAMD00212275	ST23	KL1	O1/O2v2	Japan	2017	Unknown	4	128	-	-

SAMD00212276	ST23	KL1	O1/O2v2	Japan	2017	Unknown	4	128	-	-
SAMD00212277	ST23	KL1	O1/O2v2	Japan	2018	Unknown	4	128	-	-
SAMD00212278	ST23	KL1	O1/O2v2	Japan	2018	Unknown	4	128	-	-
SAMD00212279	ST23	KL1	O1/O2v2	Japan	2018	Unknown	4	128	-	-
SAMD00287320	ST23	KL1	O1/O2v2	Japan	2017	Unknown	4	125	-	-
SAMD00287321	ST23	KL1	O1/O2v2	Japan	2017	Unknown	4	127	-	-
SAMD00287322	ST23	KL1	O1/O2v2	Japan	2017	Unknown	4	128	-	-
SAMD00287338	ST23	KL1	O1/O2v2	Japan	2018	Unknown	4	128	-	-
SAMD00287348	ST23	KL1	O1/O2v2	Japan	2018	Unknown	4	128	-	-
SAMD00287351	ST23	KL1	O1/O2v2	Japan	2018	Unknown	4	128	-	-
SAMD00287387	ST23	KL1	O1/O2v2	Japan	2018	Unknown	4	126	-	-
SAMD00287398	ST23	KL1	O1/O2v2	Japan	2018	Unknown	4	128	-	-
SAMD00287412	ST23	KL1	O1/O2v2	Japan	2018	Unknown	4	127	-	-
SAMD00287417	ST23	KL1	O1/O2v2	Japan	2019	Unknown	4	127	-	-
SAMD00287433	ST23	KL1	O1/O2v2	Japan	2019	Unknown	4	127	-	-
SAMD00399245	ST23	KL1	O1/O2v2	Japan	2016	Unknown	4	128	-	-
SAMD00399260	ST23	KL1	O1/O2v2	Japan	2017	Unknown	4	128	-	-
SAMD00399262	ST23	KL1	O1/O2v2	Japan	2016	Unknown	4	127	-	-
SAMD00399267	ST23	KL1	O1/O2v2	Japan	2016	Unknown	4	128	-	-
SAMD00399312	ST23	KL1	O1/O2v2	Japan	2017	Unknown	4	128	-	-
SAMD00399314	ST23	KL1	O1/O2v2	Japan	2017	Unknown	4	128	-	-
SAMD00399315	ST23	KL1	O1/O2v2	Japan	2017	Unknown	4	127	-	-
SAMD00399322	ST23	KL1	O1/O2v2	Japan	2017	Unknown	4	123	-	-
SAMD00399325	ST23	KL1	O1/O2v2	Japan	2017	Unknown	4	128	-	-
SAMD00399328	ST23	KL1	O1/O2v2	Japan	2016	Unknown	4	128	-	-
SAMD00399330	ST23	KL1	O1/O2v2	Japan	2016	Unknown	4	128	-	-
SAMD00399331	ST23	KL1	O1/O2v2	Japan	2016	Unknown	4	128	-	-
SAMD00399337	ST23	KL1	O1/O2v2	Japan	2016	Unknown	4	128	-	-
SAMD00399347	ST23	KL1	O1/O2v2	Japan	2016	Unknown	4	130	-	-
SAMD00399350	ST23	KL1	O1/O2v2	Japan	2016	Unknown	4	122	-	-
SAMD00399351	ST23	KL1	O1/O2v2	Japan	2016	Unknown	4	127	-	-
SAMD00399356	ST23	KL1	O1/O2v2	Japan	2016	Unknown	4	128	-	-
SAMD00399360	ST23	KL1	O1/O2v2	Japan	2016	Unknown	4	124	-	-
SAMD00399379	ST23	KL1	O1/O2v2	Japan	2015	Unknown	4	128	-	-
SAMD00399393	ST23	KL1	O1/O2v2	Japan	2015	Unknown	4	128	-	-
SAMEA13183815	ST23	KL1	O1/O2v2	Italy	2021	Liver	4	128	-	-
SAMN15541508	ST23	KL1	O1/O2v2	Vietnam	Unknown	Nose	4	127	-	-

SAMN16657683	ST23	KL1	O1/O2v2	Spain	2016	sputum	20	122	OXA-48	CTX-M-15
SAMN17080012	ST23	KL1	O1/O2v2	China	2014	Unknown	4	128	-	-
SAMN17080011	ST23	KL1	O1/O2v2	China	2014	Unknown	4	128	-	-
SAMN17080010	ST23	KL1	O1/O2v2	China	2014	Unknown	4	128	-	-
SAMN17080009	ST23	KL1	O1/O2v2	China	2014	Unknown	4	128	-	-
SAMN17080018	ST23	KL1	O1/O2v2	China	2018	Unknown	4	118	-	-
SAMN17080017	ST23	KL1	O1/O2v2	China	2018	Unknown	4	128	-	-
SAMN17080016	ST23	KL1	O1/O2v2	China	2018	Unknown	4	128	-	-
SAMN17080015	ST23	KL1	O1/O2v2	China	2017	Unknown	7	128	-	CTX-M-3
SAMN17080014	ST23	KL1	O1/O2v2	China	2010	Unknown	5	128	-	-
SAMN17080013	ST23	KL1	O1/O2v2	China	2009	Unknown	5	128	-	-
SAMN20704581	ST23	KL1	O1/O2v2	China	2020	Urine	4	128	-	-
SAMN20967296	ST23	KL1	O1/O2v2	Singapore	Unknown	Unknown	8	122	KPC-2	-
SAMN20967346	ST23	KL1	O1/O2v2	Singapore	Unknown	Unknown	8	127	KPC-2	-
SAMN20967156	ST23	KL1	O1/O2v2	Singapore	Unknown	Unknown	10	126	KPC-2	-
SAMN20967129	ST23	KL1	O1/O2v2	Singapore	Unknown	Unknown	7	127	KPC-2	-
SAMN22024788	ST23	KL1	O1/O2v2	Taiwan	Unknown	Liver	4	111	-	-
SAMN22024790	ST23	KL1	O1/O2v2	France	2008	Liver	4	128	-	-
SAMN21988161	ST23	KL1	O1/O2v2	China	Unknown	Unknown	10	129	KPC-2	CTX-M-65
SAMN23898323	ST23	KL1	O1/O2v2	China	2020	sputum	8	128	-	-
SAMN23898322	ST23	KL1	O1/O2v2	China	2020	sputum	10	128	-	CTX-M-15
SAMN24019082	ST23	KL1	O1/O2v2	United Kingdom	2015	Unknown	20	129	NDM-1	CTX-M-15;SHV-12
SAMN24020289	ST23	KL1	O1/O2v2	United Kingdom	2016	Unknown	5	129	OXA-48	-
SAMN16614929	ST23	KL1	O1/O2v2	USA	2018	Liver	4	126	-	-
SAMN16614925	ST23	KL1	O1/O2v2	USA	2018	Liver	4	123	-	-
SAMN16614890	ST23	KL1	O1/O2v2	USA	2019	Liver	4	98	-	-
SAMN25345067	ST23	KL1	O1/O2v2	Singapore	Unknown	Unknown	12	129	KPC-2	-
SAMN26366789	ST23	KL1	O1/O2v2	Australia	2001	Urine	4	128	-	-
SAMN26366787	ST23	KL1	O1/O2v2	Australia	2001	sputum	4	128	-	-
SAMN26369166	ST23	KL1	O1/O2v2	Australia	2020	sputum	4	128	-	-
SAMN26369161	ST23	KL1	O1/O2v2	Australia	2020	Urine	14	128	-	CTX-M-27
SAMN26368940	ST23	KL1	O1/O2v2	Australia	2020	Lung	4	127	-	-
SAMN26369138	ST23	KL1	O1/O2v2	Australia	2020	Blood	4	128	-	-
SAMN26369092	ST23	KL1	O1/O2v2	Australia	2020	Urine	4	128	-	-
SAMN26369012	ST23	KL1	O1/O2v2	Australia	2020	Aspirate	4	128	-	-

SAMN05581770	ST23	KL1	O1/O2v2	Denmark	Unknown	Unknown	9	129	KPC-3	-
SAMN07256684	ST23	KL1	O1/O2v2	France	1987	Animal	5	129	-	-
SAMN07256677	ST23	KL1	O1/O2v2	France	1985	Animal	9	129	-	-
SAMN07256681	ST23	KL1	O1/O2v2	France	1985	Animal	8	129	-	-
SAMEA112282253	ST23	KL1	O1/O2v2	India	Unknown	SPUTUM	4	128	-	-
SAMEA112282350	ST23	KL1	O1/O2v2	India	Unknown	SPUTUM	4	103	-	-
SAMEA112198892	ST23	KL57	O1/O2v2	Germany	2022	Urine	20	83	NDM-1	CTX-M-55
SAMEA111504893	ST23	KL1	O1/O2v2	Lithuania	2020	sputum	4	128	-	-
SAMEA111506237	ST23	KL1	O1/O2v2	Thailand	2020	sputum	4	123	-	-
SAMEA112199032	ST23	KL57	O1/O2v2	Germany	2022	Unknown	22	85	NDM-1;OXA-48	CTX-M-55
SAMEA112199047	ST23	KL57	O1/O2v2	Germany	2022	Feces	22	83	NDM-1;OXA-48	CTX-M-55
SAMEA112282345	ST23	KL1	O1/O2v2	India	Unknown	Abscess	4	128	-	-
SAMEA112198916	ST23	KL57	O1/O2v2	Germany	2022	Unknown	23	84	NDM-1	CTX-M-55
SAMEA112198947	ST23	KL57	O1/O2v2	Germany	2022	Unknown	23	84	NDM-1	CTX-M-55
SAMEA112198877	ST23	KL57	O1/O2v2	Germany	2022	Urine	24	84	NDM-1	CTX-M-55
SAMEA112199009	ST23	KL57	O1/O2v2	Germany	2022	Wound	22	86	NDM-1;OXA-48	CTX-M-55
SAMEA112199051	ST23	KL57	O1/O2v2	Germany	2022	Wound	22	85	NDM-1;OXA-48	CTX-M-55
SAMEA111504741	ST23	KL1	O1/O2v2	Hong Kong	2020	Blood	4	128	-	-
SAMEA112199024	ST23	KL57	O1/O2v2	Germany	2022	Feces	22	86	NDM-1;OXA-48	CTX-M-55
SAMEA14252882	ST23	KL1	O1/O2v2	Netherlands	2021	Unknown	4	127	-	-
SAMEA13959355	ST23	KL1	O1/O2v2	Malaysia	2016	Throat	4	101	-	-
SAMEA14252881	ST23	KL1	O1/O2v2	Netherlands	2020	Unknown	4	125	-	-
SAMEA13959343	ST23	KL1	O1/O2v2	Malaysia	2016	Throat	4	112	-	-
SAMEA14449080	ST23	KL57	O1/O2v2	Russia	2017	Lung	17	85	-	CTX-M-55
SAMEA13959378	ST23	KL1	O1/O2v2	Malaysia	2016	Throat	4	126	-	-
SAMEA13959333	ST23	KL1	O1/O2v2	Malaysia	2016	Throat	4	122	-	-
SAMEA13959321	ST23	KL1	O1/O2v2	Malaysia	2016	Throat	4	100	-	-
SAMEA9800573	ST23	KL1	O1/O2v2	Unknown	Unknown	Unknown	4	129	-	-
SAMEA9800504	ST23	KL1	O1/O2v2	Unknown	Unknown	Unknown	4	129	-	-
SAMEA112282161	ST23	KL1	O1/O2v2	India	Unknown	Abscess	4	128	-	-
SAMEA112282252	ST23	KL1	O1/O2v2	India	Unknown	Abscess	20	109	-	-
SAMEA112198976	ST23	KL57	O1/O2v2	Germany	2022	Feces	23	80	NDM-1	CTX-M-55

SAMEA112199011	ST23	KL57	O1/O2v2	Germany	2022	Wound	22	85	NDM-1;OXA-48	CTX-M-55
SAMEA111506233	ST23	KL1	O1/O2v2	Thailand	2020	Wound	4	101	-	-
SAMEA112282230	ST23	KL1	O1/O2v2	India	Unknown	Blood	9	125	-	-
SAMEA112198997	ST23	KL57	O1/O2v2	Germany	2022	Wound	22	85	NDM-1;OXA-48	CTX-M-55
SAMEA112199053	ST23	KL57	O1/O2v2	Germany	2022	Blood	29	86	NDM-1;OXA-48	CTX-M-15
SAMEA112282287	ST23	KL1	O1/O2v2	India	Unknown	Abscess	4	101	-	-
SAMEA13959369	ST23	KL1	O1/O2v2	Malaysia	2016	Throat	4	128	-	-
SAMEA13959360	ST23	KL1	O1/O2v2	Malaysia	2016	Throat	4	127	-	-
SAMEA13959324	ST23	KL1	O1/O2v2	Malaysia	2016	Throat	4	101	-	-
SAMEA13959361	ST23	KL1	O1/O2v2	Malaysia	2016	Throat	4	128	-	-
SAMEA9800611	ST23	KL1	O1/O2v2	Unknown	Unknown	Unknown	4	94	-	-
SAMEA13959330	ST23	KL1	O1/O2v2	Malaysia	2016	Throat	4	122	-	-
SAMEA9800570	ST23	KL1	O1/O2v2	Unknown	Unknown	Unknown	4	128	-	-
SAMEA8457514	ST23	KL1	O1/O2v1	Unknown	Unknown	Unknown	4	126	-	-
SAMEA13959332	ST23	KL1	O1/O2v2	Malaysia	2016	Throat	4	128	-	-
SAMEA13959356	ST23	KL1	O1/O2v2	Malaysia	2016	Throat	4	98	-	-
SAMEA13959377	ST23	KL1	O1/O2v2	Malaysia	2016	Throat	4	128	-	-
SAMEA13959364	ST23	KL1	O1/O2v2	Malaysia	2016	Throat	4	128	-	-
SAMEA13959354	ST23	KL1	O1/O2v2	Malaysia	2016	Throat	4	101	-	-
SAMEA13959373	ST23	KL1	O1/O2v2	Malaysia	2016	Throat	4	126	-	-
SAMN31807500	ST23	KL57	O1/O2v2	Netherlands	2022	Unknown	20	85	NDM-1	CTX-M-55
SAMN17981817	ST23	KL1	O1/O2v2	Singapore	2019	Blood	9	128	KPC-2	-
SAMN24256183	ST23	KL1	O1/O2v2	China	2017	Unknown	10	128	-	-
SAMN24256184	ST23	KL1	O1/O2v2	China	2015	Unknown	16	129	KPC-2	-
SAMN31807475	ST23	KL57	O1/O2v2	Netherlands	2022	Unknown	22	86	NDM-1;OXA-48	CTX-M-55
SAMN31269458	ST23	KL1	O1/O2v2	Unknown	2018	Unknown	10	122	OXA-48	CTX-M-15
SAMN31269464	ST23	KL1	O1/O2v2	Unknown	2019	Unknown	9	117	OXA-48	CTX-M-15
SAMN24256192	ST23	KL1	O1/O2v2	China	2014	Unknown	24	123	KPC-2	CTX-M-14
SAMN30274886	ST23	KL1	O1/O2v2	USA	2022	Liver	4	128	-	-
SAMN30383139	ST23	KL1	O1/O2v2	China	2017	Unknown	4	128	-	-
SAMN30274884	ST23	KL1	O1/O2v2	USA	2021	Liver	4	126	-	-
SAMN31269462	ST23	KL1	O1/O2v2	Unknown	2019	Unknown	10	123	OXA-48	CTX-M-15
SAMN31807489	ST23	KL57	O1/O2v2	Netherlands	2022	Unknown	27	86	NDM-1	CTX-M-55
SAMN24256185	ST23	KL1	O1/O2v2	China	2017	Unknown	10	128	KPC-2	CTX-M-65

SAMD00501837	ST23	KL1	O1/O2v2	Japan	2019	sputum	5	128	-	CTX-M-14*
SAMD00498312	ST23	KL1	O1/O2v2	Japan	2020	Urine	7	128	-	CTX-M-14
SAMD00501568	ST23	KL1	O1/O2v1	Japan	2019	Lung	4	121	-	-
SAMD00502488	ST23	KL1	O1/O2v2	Japan	2019	sputum	16	127	-	-
SAMD00498165	ST23	KL1	O1/O2v2	Japan	2020	sputum	11	101	-	CTX-M-14
SAMD00499489	ST23	KL1	O1/O2v2	Japan	2020	Urine	6	128	-	CTX-M-14
SAMD00502511	ST23	KL1	O1/O2v2	Japan	2019	sputum	17	128	-	-
SAMD00502460	ST23	KL1	O1/O2v2	Japan	2019	Blood	16	126	-	-
SAMD00502456	ST23	KL1	O1/O2v2	Japan	2019	Blood	21	126	-	CTX-M-14
SAMD00502446	ST23	KL1	O1/O2v2	Japan	2019	Unknown	13	127	-	-
SAMD00498163	ST23	KL1	O1/O2v2	Japan	2020	sputum	11	128	-	CTX-M-14
SAMEA114094075	ST23	KL1	O1/O2v2	Vietnam	2016	Blood	4	125	-	-
SAMEA114094087	ST23	KL1	O1/O2v2	Vietnam	2018	Blood	4	124	-	-
SAMEA114094106	ST23	KL113	O1/O2v2	Vietnam	2019	Blood	4	95	-	-
SAMEA114228262	ST23	KL1	O1/O2v2	Germany	2022	Food	4	121	-	-
SAMEA114094130	ST23	KL1	O1/O2v2	Vietnam	2020	Blood	4	120	-	-
SAMD00397314	ST23	KL1	O1/O2v2	Japan	2018	Unknown	4	127	-	-
SAMEA114094101	ST23	KL1	O1/O2v2	Vietnam	2012	Blood	4	115	-	-
SAMN28747366	ST23	KL1	O1/O2v2	Spain	2012	Blood	4	126	-	-
SAMEA114094124	ST23	KL1	O1/O2v2	Vietnam	2020	Blood	4	122	-	-
SAMEA114094080	ST23	KL1	O1/O2v2	Vietnam	2018	Blood	4	109	-	-
SAMEA114094051	ST23	KL1	O1/O2v2	Vietnam	2014	Blood	4	127	-	-
SAMEA114094070	ST23	KL1	O1/O2v2	Vietnam	2016	Blood	4	123	-	-
SAMN30274883	ST23	KL1	O1/O2v2	USA	2021	Liver	4	128	-	-
SAMEA114094096	ST23	KL1	O1/O2v2	Vietnam	2018	Blood	4	110	-	-
SAMEA114094093	ST23	KL1	O1/O2v2	Vietnam	2018	Blood	4	127	-	-
SAMN31309194	ST23	KL57	O1/O2v2	Germany	2022	Unknown	20	85	NDM-1	CTX-M-55
SAMEA114094082	ST23	KL1	O1/O2v2	Vietnam	2010	Blood	4	107	-	-
SAMEA114094107	ST23	KL1	O1/O2v2	Vietnam	2019	Blood	4	126	-	-
SAMEA114094058	ST23	KL1	O1/O2v1	Vietnam	2015	Blood	4	113	-	-
SAMEA114094094	ST23	KL1	O1/O2v2	Vietnam	2018	Blood	4	125	-	-
SAMN33768328	ST23	KL57	O1/O2v2	Denmark	2021	Unknown	28	86	NDM-1;OXA-48	CTX-M-15
SAMN33797289	ST23	KL1	O1/O2v2	Cambodia	2022	Blood	4	112	-	-
SAMN36687980	ST23	KL1	O1/O2v2	China	Unknown	Unknown	6	127	NDM-1	-
SAMN34411498	ST23	KL1	O1/O2v2	Cambodia	2022	Blood	4	127	-	-
SAMN34098886	ST23	KL1	O1/O2v2	France	2022	Unknown	4	111	-	-

SAMN35037654	ST23	KL1	O1/O2v2	Chile	2022	Aspirate	7	127	KPC-2	-
SAMD00397246	ST23	KL1	O1/O2v2	Japan	2014	Unknown	10	125	-	CTX-M-2
SAMD00397221	ST23	KL1	O1/O2v2	Japan	2016	Unknown	4	127	-	-
SAMD00397218	ST23	KL113	O1/O2v1	Japan	2016	Unknown	4	89	-	-
SAMEA114094129	ST23	KL1	O1/O2v2	Vietnam	2020	Blood	4	128	-	-
SAMEA114094090	ST23	KL1	O1/O2v2	Vietnam	2018	Blood	4	124	-	-
SAMEA114094118	ST23	KL1	O1/O2v2	Vietnam	2020	Blood	4	128	-	-
SAMEA114094049	ST23	KL1	O1/O2v2	Vietnam	2010	Blood	4	124	-	-
SAMEA114094100	ST23	KL1	O1/O2v2	Vietnam	2018	Blood	4	127	-	-
SAMEA114094048	ST23	KL1	O1/O2v2	Vietnam	2014	Blood	4	121	-	-
SAMEA114094132	ST23	KL1	O1/O2v2	Vietnam	2020	Blood	4	128	-	-
SAMEA114094119	ST23	KL1	O1/O2v2	Vietnam	2019	Blood	4	122	-	-
SAMEA114094105	ST23	KL1	O1/O2v2	Vietnam	2019	Blood	4	125	-	-
SAMEA114094073	ST23	KL1	O1/O2v2	Vietnam	2017	Blood	4	118	-	-
SAMEA114094077	ST23	KL1	O1/O2v2	Vietnam	2017	Blood	4	125	-	-
SAMEA114094052	ST23	KL1	O1/O2v2	Vietnam	2015	Blood	4	124	-	-
SAMEA114094083	ST23	KL1	O1/O2v2	Vietnam	2018	Blood	8	111	-	-
SAMEA114094079	ST23	KL1	O1/O2v2	Vietnam	2017	Blood	4	125	-	-
SAMEA114094068	ST23	KL1	O1/O2v2	Vietnam	2016	Blood	4	127	-	-
SAMEA114228259	ST23	KL1	O1/O2v2	Germany	2022	Blood	4	119	-	-
SAMN28743185	ST23	KL1	O1/O2v2	Australia	2019	Abdomen fluid	5	113	-	CTX-M-14
SAMN30274894	ST23	KL1	O1/O2v2	USA	2022	Abdomen fluid	4	130	-	-
SAMN30274889	ST23	KL1	O1/O2v2	USA	2022	Aspirate	4	128	-	-
SAMN29894068	ST23	KL57	O1/O2v2	USA	2022	Urine	17	83	OXA-48	CTX-M-55
SAMN34098889	ST23	KL3	O1/O2v2	France	2022	Unknown	4	98	-	-
SAMN36470478	ST23	KL1	O1/O2v1	Russia	2023	Lung	13	123	-	CTX-M-15
SAMN33768325	ST23	KL57	O1/O2v2	Denmark	2021	Unknown	28	85	NDM-1;OXA-48	CTX-M-15
SAMN31309209	ST23	KL57	O1/O2v2	Germany	2022	Unknown	22	86	NDM-1;OXA-48	CTX-M-55
SAMN30274890	ST23	KL1	O1/O2v2	USA	2022	Neck	4	128	-	-
SAMN30274887	ST23	KL1	O1/O2v2	USA	2020	Eye	4	123	-	-
SAMEA113605108	ST23	KL1	O1/O2v2	Norway	2017	Blood	4	124	-	-
SAMD00502452	ST23	KL1	O1/O2v2	Japan	2019	Urine	20	126	-	CTX-M-14
SAMEA113605938	ST23	KL1	O1/O2v2	Norway	2017	Blood	4	127	-	-
SAMEA113606020	ST23	KL1	O1/O2v2	Norway	2018	Blood	5	116	-	-
SAMD00501667	ST23	KL1	O1/O2v2	Japan	2019	Unknown	10	101	-	CTX-M-14
SAMN24256182	ST23	KL1	O1/O2v2	China	2015	Unknown	16	129	KPC-2	-

SAMD00499532	ST23	KL1	O1/O2v2	Japan	2020	Urine	4	115	-	-
SAMD00498948	ST23	KL1	O1/O2v2	Japan	2019	sputum	9	128	-	CTX-M-2
SAMN14649975	ST23	KL1	O1/O2v1	Singapore	2018	Sputum	13	119	KPC-2	-
SAMD00501310	ST23	KL1	O1/O2v2	Japan	2019	Abscess	15	130	-	CTX-M-15
SAMN28955526	ST23	KL1	O1/O2v2	China	2020	Environment	4	111	-	-
SAMN30274888	ST23	KL1	O1/O2v2	USA	2021	Aspirate	4	128	-	-
SAMN26937857	ST23	KL1	O1/O2v2	China	2020	Unknown	6	111	KPC-2	-
SAMN25184611	ST23	KL1	O1/O2v2	China	2021	Wound	4	128	-	-
SAMN30816129	ST23	KL1	O1/O2v2	Australia	2020	Blood	4	128	-	-
SAMN27533723	ST23	KL1	O1/O2v2	USA	Unknown	Blood	5	128	-	-
SAMN28174100	ST23	KL1	O1/O2v2	USA	2019	Blood	24	128	-	CTX-M-15
SAMN30274892	ST23	KL1	O1/O2v2	USA	2022	Lung	4	128	-	-
SAMN30274891	ST23	KL1	O1/O2v2	USA	2021	Blood	4	130	-	-
SAMN26816074	ST23	KL1	O1/O2v2	Taiwan	2020	Abscess	5	128	-	-
SAMN26976436	ST23	KL1	O1/O2v2	USA	2018	Feces	5	124	KPC-2	-
SAMD00497777	ST23	KL1	O1/O2v2	Japan	2019	sputum	6	128	-	CTX-M-14
SAMD00497586	ST23	KL1	O1/O2v2	Japan	2019	sputum	10	128	-	CTX-M-8
SAMD00397239	ST23	KL1	O1/O2v2	Japan	2016	Unknown	4	126	-	-
SAMEA114094045	ST23	KL1	O1/O2v2	Vietnam	2013	Blood	4	125	-	-
SAMEA114094042	ST23	KL1	O1/O2v2	Vietnam	2013	Blood	4	126	-	-
SAMEA114094122	ST23	KL1	O1/O2v2	Vietnam	2019	Blood	4	127	-	-
SAMEA114094064	ST23	KL1	O1/O2v2	Vietnam	2016	Blood	9	123	-	-
SAMD00397263	ST23	KL1	O1/O2v2	Japan	2014	Unknown	5	127	-	-
SAMEA114094088	ST23	KL1	O1/O2v1	Vietnam	2018	Blood	4	118	-	-
SAMEA114094110	ST23	KL1	O1/O2v2	Vietnam	2019	Blood	4	124	-	-
SAMEA114094056	ST23	KL1	O1/O2v1	Vietnam	2015	Blood	4	101	-	-
SAMD00397271	ST23	KL1	O1/O2v2	Japan	2015	Unknown	10	127	-	CTX-M-2
SAMEA114094086	ST23	KL1	O1/O2v2	Vietnam	2018	Blood	4	115	-	-
SAMD00397277	ST23	KL1	O1/O2v2	Japan	2015	Unknown	5	124	-	-
SAMN34098885	ST23	KL156-D1	O1/O2v2	France	2022	Unknown	4	93	-	-
SAMN26816067	ST23	KL1	O1/O2v2	Taiwan	2020	Abscess	4	128	-	-
SAMN25349844	ST23	KL1	O1/O2v2	Singapore	Unknown	Unknown	9	128	KPC-2	-
SAMN25349857	ST23	KL1	O1/O2v2	Singapore	Unknown	Unknown	8	122	-	-
SAMN26816068	ST23	KL1	O1/O2v2	Taiwan	2020	Abscess	4	128	-	-
SAMN25349852	ST23	KL1	O1/O2v2	Singapore	Unknown	Unknown	9	128	KPC-2	-
SAMN29204929	ST23	KL1	O1/O2v2	Bangladesh	2007	Unknown	16	112	-	CTX-M-15
SAMN28174101	ST23	KL1	O1/O2v2	USA	2019	Blood	24	128	-	CTX-M-15

SAMN25349861	ST23	KL1	O1/O2v2	Singapore	Unknown	Unknown	4	128	-	-
SAMN26816072	ST23	KL1	O1/O2v2	Taiwan	2020	Abscess	5	128	-	-
SAMN25349859	ST23	KL1	O1/O2v2	Singapore	Unknown	Unknown	4	118	-	-
SAMN25349851	ST23	KL1	O1/O2v2	Singapore	Unknown	Unknown	9	128	KPC-2	-
SAMN34098887	ST23	KL2	O1/O2v2	France	2022	Unknown	4	99	-	-
SAMN36663133	ST23	KL1	O1/O2v2	China	Unknown	Unknown	6	128	-	CTX-M-15
SAMN25041863	ST23	KL1	O1/O2v2	Guadeloupe	2019	Unknown	4	128	-	-
SAMN25041656	ST23	KL1	O1/O2v2	Guadeloupe	2019	Unknown	4	126	-	-
SAMN25349849	ST23	KL1	O1/O2v2	Singapore	Unknown	Unknown	9	118	KPC-2	-
SAMEA112785770	ST23	KL57	O1/O2v2	Unknown	Unknown	Unknown	22	85	NDM-1;OXA-48	CTX-M-55
SAMD00502501	ST23	KL1	O1/O2v2	Japan	2019	sputum	15	127	-	-
SAMEA113605721	ST23	KL113	O1/O2v2	Norway	2018	Blood	4	114	-	-
SAMEA113605381	ST23	KL1	O1/O2v2	Norway	2017	Blood	4	127	-	-
SAMEA113605045	ST23	KL1	O1/O2v2	Norway	2018	Blood	4	128	-	-
SAMEA113606003	ST23	KL1	O1/O2v2	Norway	2018	Blood	4	129	-	-
SAMEA113605019	ST23	KL1	O1/O2v2	Norway	2017	Blood	4	124	-	-
SAMEA113606014	ST23	KL107	O1/O2v2	Norway	2018	Blood	4	116	-	-
SAMEA113605723	ST23	KL113	O1/O2v2	Norway	2018	Blood	4	113	-	-
SAMEA113605358	ST23	KL1	O1/O2v2	Norway	2017	Blood	5	123	-	-
SAMN26567295	ST23	KL1	O1/O2v2	Japan	2019	Unknown	4	128	-	-
SAMN26976296	ST23	KL1	O1/O2v2	USA	2017	Feces	4	128	-	-
SAMN30525618	ST23	KL1	O1/O2v2	Pakistan	2018	Unknown	4	111	-	-
SAMN25349847	ST23	KL1	O1/O2v2	Singapore	Unknown	Unknown	9	118	KPC-2	-
SAMN25349854	ST23	KL1	O1/O2v2	Singapore	Unknown	Unknown	4	118	-	-
SAMN25041684	ST23	KL1	O1/O2v2	Guadeloupe	2019	Unknown	4	114	-	-
SAMN29204931	ST23	KL1	O1/O2v2	Bangladesh	2007	Unknown	4	122	-	-
SAMN28955527	ST23	KL1	O1/O2v2	China	2020	Environment	4	111	-	-
SAMN25349845	ST23	KL1	O1/O2v2	Singapore	Unknown	Unknown	9	127	KPC-2	-
SAMN27533751	ST23	KL1	O1/O2v2	USA	Unknown	Blood	4	128	-	-
SAMN25349848	ST23	KL1	O1/O2v2	Singapore	Unknown	Unknown	9	118	KPC-2	-
SAMN25349858	ST23	KL1	O1/O2v2	Singapore	Unknown	Unknown	4	128	-	-
SAMN26567294	ST23	KL1	O1/O2v2	Japan	2018	Unknown	4	126	-	-
SAMN26816073	ST23	KL1	O1/O2v2	Taiwan	2020	Abscess	4	128	-	-
SAMN25349855	ST23	KL1	O1/O2v2	Singapore	Unknown	Unknown	4	118	-	-
SAMN25349850	ST23	KL1	O1/O2v2	Singapore	Unknown	Unknown	9	118	KPC-2	-

SAMN26816071	ST23	KL1	O1/O2v2	Taiwan	2020	Abscess	4	129	-	-
SAMN28955528	ST23	KL1	O1/O2v2	China	2020	Environment	4	111	-	-
SAMN26816070	ST23	KL1	O1/O2v2	Taiwan	2020	Abscess	4	128	-	-
SAMN25349846	ST23	KL1	O1/O2v2	Singapore	Unknown	Unknown	9	118	KPC-2	-
SAMN26976476	ST23	KL1	O1/O2v2	USA	2018	Unknown	5	124	KPC-2	-
SAMN25184617	ST23	KL1	O1/O2v2	China	2021	sputum	4	128	-	-
SAMN25349862	ST23	KL1	O1/O2v2	Singapore	Unknown	Unknown	9	128	KPC-2	-
SAMN26816069	ST23	KL1	O1/O2v2	Taiwan	2020	Abscess	4	128	-	-
SAMN27533733	ST23	KL1	O1/O2v2	USA	Unknown	Blood	4	128	-	-
SAMN25349860	ST23	KL1	O1/O2v2	Singapore	Unknown	Unknown	4	128	-	-
SAMN32151078	ST23	KL1	O1/O2v2	India	2020	Urine	4	112	-	-
SAMN25349856	ST23	KL1	O1/O2v2	Singapore	Unknown	Unknown	4	122	-	-
SAMN25041580	ST23	KL1	O1/O2v2	Guadeloupe	2018	Unknown	4	117	-	-
SAMN25349853	ST23	KL1	O1/O2v2	Singapore	Unknown	Unknown	4	118	-	-
SAMN25041577	ST23	KL1	O1/O2v2	Guadeloupe	2018	Unknown	4	125	-	-
SAMN26816075	ST23	KL1	O1/O2v2	Taiwan	2020	Abscess	5	128	-	-
SAMN34098888	ST23	KL24	O1/O2v2	France	2022	Unknown	4	99	-	-
SAMD00502480	ST23	KL1	O1/O2v2	Japan	2020	Urine	11	122	-	-
SAMD00502534	ST23	KL1	O1/O2v2	Japan	2020	Unknown	13	128	-	CTX-M-15
SAMD00498218	ST23	KL1	O1/O2v2	Japan	2020	Blood	11	128	-	CTX-M-14
SAMD00501953	ST23	KL1	O1/O2v2	Japan	2019	sputum	5	127	-	-
SAMD00498800	ST23	KL1	O1/O2v2	Japan	2019	Urine	8	127	-	CTX-M-14
SAMD00502455	ST23	KL1	O1/O2v2	Japan	2019	Blood	20	124	-	CTX-M-14
SAMD00500785	ST23	KL1	O1/O2v2	Japan	2019	Feces	11	128	IMP-6	CTX-M-2
SAMD00497798	ST23	KL1	O1/O2v2	Japan	2020	sputum	11	113	IMP-1	CTX-M-2
SAMD00498215	ST23	KL1	O1/O2v2	Japan	2020	Wound	4	128	-	-
SAMN34258006	ST23	KL1	O1/O2v2	United Kingdom	2022	Blood	4	128	-	-
SAMD00497784	ST23	KL1	O1/O2v2	Japan	2019	sputum	6	127	-	CTX-M-14
SAMN07259332	ST23	KL1	O1/O2v2	China	2013	Unknown	9	130	KPC-2	CTX-M-55
SAMN08915824	ST23	KL1	O1/O2v2	China	2017	sputum	22	131	NDM-1	CTX-M-14;CTX-M-14
SAMN10461964	ST23	KL1	O1/O2v2	China	2015	Unknown	16	131	VIM-1	CTX-M-15
SAMN17108663	ST23	KL1	O1/O2v2	China	2017	sputum	5	120	KPC-2	-
SAMN26241723	ST23	KL1	O1/O2v2	China	2018	Blood	6	129	KPC-2	-
SAMN28515229	ST23	KL1	O1/O2v2	China	2020	Unknown	7	130	NDM-1	SHV-12
SAMN34111323	ST23	KL1	O1/O2v2	China	2018	Blood	5	112	OXA-48	-

SAMN37347794	ST23	KL1	O1/O2v2	China	2015	sputum	16	131	KPC-2	-
SAMN37347821	ST23	KL1	O1/O2v2	China	2016	sputum	7	130	NDM-1	SHV-12
SAMN37347822	ST23	KL1	O1/O2v2	China	2016	sputum	7	130	NDM-1	SHV-12
SAMN37347836	ST23	KL1	O1/O2v2	China	2014	Blood	26	125	KPC-2	CTX-M-14
SAMN37347837	ST23	KL1	O1/O2v2	China	2017	Blood	12	131	NDM-5	-
SAMN37347864	ST23	KL1	O1/O2v2	China	2015	sputum	16	131	KPC-2	-
SAMN37347871	ST23	KL1	O1/O2v2	China	2017	sputum	10	130	KPC-2	CTX-M-65
SAMN37347890	ST23	KL1	O1/O2v2	China	2016	Urine	6	130	KPC-2	-
SAMN39898430	ST23	KL1	O1/O2v2	China	2021	sputum	6	130	KPC-3	-
SAMD00060934	ST23	KL1	O1/O2v2	China	Unknown	Blood	4	112	-	-
SAMN06200334	ST23	KL1	O1/O2v2	China	2015	sputum	8	112	-	SHV-12;SHV-12
SAMN08714644	ST23	KL1	O1/O2v2	China	2017	sputum	18	131	KPC-2	CTX-M-14
SAMN08714730	ST23	KL1	O1/O2v2	China	2017	Food	17	131	KPC-2	CTX-M-15
SAMN39743267	ST23	KL1	O1/O2v2	China	2019	sputum	4	130	-	-
SAMN02603641	ST23	KL1	O1/O2v2	Unknown	Unknown	Unknown	4	120	-	-
SAMN05945909	ST23	KL1	O1/O2v2	China	2012	Unknown	4	98	-	-
SAMN05945851	ST23	KL1	O1/O2v2	China	2012	Unknown	4	127	-	-
SAMN07345013	ST23	KL1	O1/O2v2	China	2014	Blood	6	129	NDM-1	-
SAMN07345006	ST23	KL1	O1/O2v2	China	2015	Blood	7	125	KPC-2	-
SAMN05231874	ST23	KL1	O1/O2v2	China	2007	Sputum	4	130	-	-
SAMN03280393	ST23	KL1	O1/O2v2	Unknown	2014	Unknown	4	130	-	-
SAMN08117209	ST23	KL1	O1/O2v2	China	2009	Unknown	4	101	-	-
SAMN03280412	ST23	KL1	O1/O2v2	Unknown	2013	Unknown	4	125	-	-
SAMN03280413	ST23	KL1	O1/O2v2	Unknown	2014	Unknown	4	130	-	-
SAMN08117205	ST23	KL1	O1/O2v2	China	2009	Unknown	4	92	-	-
SAMN08281238	ST23	KL1	O1/O2v2	China	2012	Liver	4	115	-	-
SAMN08281172	ST23	KL1	O1/O2v2	China	2009	Unknown	4	129	-	-
SAMN08281171	ST23	KL1	O1/O2v2	China	2010	sputum	4	130	-	-
SAMN08116692	ST23	KL1	O1/O2v2	China	2009	Unknown	4	101	-	-
SAMN08578631	ST23	KL1	O1/O2v2	Unknown	2014	Unknown	16	114	OXA-48	CTX-M-15
SAMN08582315	ST23	KL1	O1/O2v2	China	2008	Wound	4	120	-	-
SAMN09736899	ST23	KL1	O1/O2v2	China	2013	Feces	4	128	-	-
SAMEA4364605	ST23	KL1	O1/O2v2	Thailand	2016	Unknown	5	130	-	-
SAMN08366844	ST23	KL1	O1/O2v2	China	2014	Unknown	5	123	-	CTX-M-24
SAMN05250855	ST23	KL1	O1/O2v2	India	2016	Blood	4	106	-	-
SAMN05413133	ST23	KL1	O1/O2v2	India	2015	Blood	4	128	-	-
SAMN10790857	ST23	KL1	O1/O2v2	China	2017	Blood	4	130	-	-

SAMN10908921	ST23	KL1	O1/O2v2	India	2018	Blood	18	110	-	CTX-M-15
SAMN10956502	ST23	KL1	O1/O2v2	India	2017	Blood	24	109	OXA-232	CTX-M-15;CTX-M-15
SAMD00129515	ST23	KL1	O1/O2v2	Japan	2013	Blood	4	127	-	-
SAMD00129540	ST23	KL1	O1/O2v2	Japan	2014	Blood	4	130	-	-
SAMD00129546	ST23	KL1	O1/O2v2	Japan	2014	Blood	4	130	-	-
SAMD00129551	ST23	KL1	O1/O2v2	Japan	2014	Blood	4	130	-	-
SAMD00129566	ST23	KL1	O1/O2v2	Japan	2014	Blood	4	130	-	-
SAMD00129567	ST23	KL1	O1/O2v2	Japan	2014	Blood	4	120	-	-
SAMD00129599	ST23	KL1	O1/O2v2	Japan	2014	Blood	4	130	-	-
SAMD00129641	ST23	KL1	O1/O2v2	Japan	2014	Blood	5	101	-	-
SAMD00163851	ST23	KL1	O1/O2v2	Japan	2013	Urine	4	120	-	-
SAMEA104567926	ST23	KL1	O1/O2v2	Grenada	Unknown	Unknown	4	129	-	-
SAMN12607206	ST23	KL1	O1/O2v2	Unknown	2017	Unknown	7	128	-	-
SAMN12607205	ST23	KL1	O1/O2v2	Unknown	2017	Unknown	4	128	-	-
SAMN12330908	ST23	KL1	O1/O2v2	China	2016	sputum	4	128	-	-
SAMN12330906	ST23	KL1	O1/O2v2	China	2016	sputum	4	128	-	-
SAMN12330905	ST23	KL1	O1/O2v1	China	2016	sputum	5	93	-	-
SAMN12330903	ST23	KL1	O1/O2v2	China	2016	sputum	4	127	-	-
SAMN12868600	ST23	KL1	O1/O2v2	China	2017	Abdomen fluid	4	128	-	-
SAMN12868598	ST23	KL1	O1/O2v2	China	2017	Liver	4	128	-	-
SAMN08366847	ST23	KL1	O1/O2v2	China	2009	Unknown	4	112	-	-
SAMN13153750	ST23	KL57	O1/O2v2	Russia	2017	Blood	12	85	OXA-48	-
SAMN13153743	ST23	KL57	O1/O2v2	Russia	2017	Aspirate	19	86	-	CTX-M-55
SAMN13701845	ST23	KL1	O1/O2v2	Ukraine	2014	Wound	4	128	-	-
SAMN08324158	ST23	KL1	O1/O2v2	Italy	2015	Cerebrospinal fluid	5	129	KPC-2	-
SAMEA6099336	ST23	KL1	O1/O2v2	China	2009	Unknown	4	127	-	-
SAMEA6099343	ST23	KL1	O1/O2v2	China	2010	Unknown	4	128	-	-
SAMEA6099341	ST23	KL1	O1/O2v2	China	2008	Unknown	4	110	-	-
SAMEA6099344	ST23	KL1	O1/O2v2	China	2009	Unknown	4	110	-	-
SAMEA6099337	ST23	KL1	O1/O2v2	China	2009	Unknown	4	128	-	-
SAMEA6099345	ST23	KL1	O1/O2v2	China	2009	Unknown	4	124	-	-
SAMEA6099346	ST23	KL1	O1/O2v2	China	2009	Unknown	4	128	-	-
SAMEA6099349	ST23	KL1	O1/O2v2	China	2010	Unknown	4	128	-	-
SAMEA6099338	ST23	KL1	O1/O2v2	China	2009	Unknown	4	128	-	-
SAMEA6099362	ST23	KL1	O1/O2v2	China	2011	Unknown	4	128	-	-

SAMEA6099361	ST23	KL1	O1/O2v2	China	2011	Unknown	4	128	-	-
SAMEA6099372	ST23	KL1	O1/O2v2	China	2012	Unknown	4	128	-	-
SAMEA6099371	ST23	KL1	O1/O2v2	China	2012	Unknown	4	128	-	-
SAMEA6099354	ST23	KL1	O1/O2v2	China	2011	Unknown	4	128	-	-
SAMEA6099366	ST23	KL1	O1/O2v2	China	2011	Unknown	5	128	-	-
SAMEA6099353	ST23	KL1	O1/O2v2	China	2011	Unknown	4	128	-	-
SAMEA6099370	ST23	KL1	O1/O2v2	China	2012	Unknown	6	128	-	-
SAMEA6099358	ST23	KL1	O1/O2v2	China	2011	Unknown	5	128	-	-
SAMEA6099359	ST23	KL1	O1/O2v2	China	2011	Unknown	11	128	-	CTX-M-14
SAMEA6099374	ST23	KL1	O1/O2v2	China	2012	Unknown	4	127	-	-
SAMEA6099379	ST23	KL1	O1/O2v2	China	2012	Unknown	4	128	-	-
SAMEA6099380	ST23	KL1	O1/O2v2	China	2012	Unknown	4	128	-	-
SAMEA6099460	ST23	KL1	O1/O2v2	China	2017	Unknown	8	129	-	-
SAMEA6099462	ST23	KL1	O1/O2v2	China	2017	Unknown	9	128	-	-
SAMEA6099464	ST23	KL1	O1/O2v2	China	2017	Unknown	9	129	-	-
SAMEA6099554	ST23	KL1	O1/O2v2	China	2017	Unknown	7	129	-	-
SAMEA6099553	ST23	KL1	O1/O2v2	China	2017	Unknown	10	130	-	-
SAMEA6099456	ST23	KL1	O1/O2v2	China	2017	Unknown	15	130	-	CTX-M-3
SAMEA6099571	ST23	KL1	O1/O2v2	China	2017	Unknown	5	128	-	-
SAMEA6099578	ST23	KL1	O1/O2v2	China	2017	Unknown	8	122	-	-
SAMEA6099575	ST23	KL1	O1/O2v2	China	2017	Unknown	8	128	-	-
SAMEA6099579	ST23	KL1	O1/O2v2	China	2017	Unknown	4	128	-	-
SAMEA6099573	ST23	KL1	O1/O2v2	China	2017	Unknown	4	127	-	-
SAMEA6099580	ST23	KL1	O1/O2v2	China	2017	Unknown	4	128	-	-
SAMEA6099598	ST23	KL1	O1/O2v2	China	2017	Unknown	5	128	-	-
SAMEA6099601	ST23	KL1	O1/O2v2	China	2017	Unknown	4	128	-	-
SAMEA6099478	ST23	KL1	O1/O2v2	China	2017	Unknown	4	128	-	-
SAMEA6099479	ST23	KL1	O1/O2v2	China	2017	Unknown	4	128	-	-
SAMEA6099475	ST23	KL1	O1/O2v2	China	2017	Unknown	8	128	-	-
SAMEA6099477	ST23	KL1	O1/O2v2	China	2017	Unknown	4	123	-	-
SAMEA6099483	ST23	KL1	O1/O2v2	China	2017	Unknown	4	128	-	-
SAMEA6099482	ST23	KL1	O1/O2v2	China	2017	Unknown	4	128	-	-
SAMEA6099491	ST23	KL1	O1/O2v2	China	2017	Unknown	4	128	-	-
SAMEA6099500	ST23	KL1	O1/O2v2	China	2017	Unknown	4	128	-	-
SAMEA6099504	ST23	KL1	O1/O2v2	China	2017	Unknown	4	128	-	-
SAMEA6099507	ST23	KL1	O1/O2v2	China	2017	Unknown	4	128	-	-
SAMEA6099494	ST23	KL1	O1/O2v2	China	2017	Unknown	4	129	-	-

SAMEA6099501	ST23	KL1	O1/O2v2	China	2017	Unknown	4	128	-	-
SAMEA6099511	ST23	KL1	O1/O2v2	China	2017	Unknown	4	128	-	-
SAMEA6099532	ST23	KL1	O1/O2v2	China	2017	Unknown	4	128	-	-
SAMEA6099526	ST23	KL1	O1/O2v2	China	2017	Unknown	4	128	-	-
SAMEA6099535	ST23	KL1	O1/O2v2	China	2017	Unknown	4	128	-	-
SAMEA6099534	ST23	KL1	O1/O2v2	China	2017	Unknown	4	128	-	-
SAMEA6099536	ST23	KL1	O1/O2v2	China	2017	Unknown	4	128	-	-
SAMEA6099537	ST23	KL1	O1/O2v2	China	2017	Unknown	4	128	-	-
SAMEA6099543	ST23	KL1	O1/O2v2	China	2017	Unknown	4	128	-	-
SAMN14277886	ST23	KL1	O1/O2v2	China	2017	Feces	4	130	-	-
SAMEA6939103	ST23	KL1	O1/O2v2	France	2011	Cerebrospinal fluid	4	127	-	-
SAMEA6939106	ST23	KL1	O1/O2v2	France	2013	Cerebrospinal fluid	4	127	-	-
SAMN16124401	ST23	KL1	O1/O2v2	India	2019	Blood	5	103	OXA-232	-
SAMN16386297	ST23	KL1	O1/O2v2	Russia	2014	Wound	16	129	OXA-48	CTX-M-15
SAMN16560600	ST23	KL1	O1/O2v2	China	2019	Feces	4	130	-	-
SAMN16560591	ST23	KL1	O1/O2v2	China	2019	Feces	4	129	-	-
SAMN16561436	ST23	KL1	O1/O2v2	China	2018	Blood	4	130	-	-
SAMN16561437	ST23	KL1	O1/O2v2	China	2018	Blood	4	130	-	-
SAMN16561429	ST23	KL1	O1/O2v2	China	2018	Unknown	5	130	KPC-2	-
SAMN16561285	ST23	KL1	O1/O2v2	China	2018	Unknown	5	130	KPC-2	-
SAMEA3357081	ST23	KL1	O1/O2v2	Unknown	Unknown	Unknown	4	130	-	-
SAMN15415360	ST23	KL1	O1/O2v2	France	2020	Blood	7	128	-	-
SAMN15415385	ST23	KL1	O1/O2v2	France	2017	Blood	4	126	-	-
SAMN13301699	ST23	KL1	O1/O2v2	Australia	2013	Blood	4	130	-	-
SAMN13301666	ST23	KL1	O1/O2v2	Australia	2014	Blood	4	130	-	-
SAMN13301662	ST23	KL1	O1/O2v2	Australia	2014	Blood	4	130	-	-
SAMN17766239	ST23	KL1	O1/O2v2	China	2019	Eye	4	130	-	-
SAMEA7846860	ST23	KL40	O1/O2v2	China	Unknown	Feces	4	101	-	-
SAMN18450111	ST23	KL1	O1/O2v2	China	2020	sputum	4	129	-	-
SAMN13741660	ST23	KL1	O1/O2v2	China	2016	sputum	5	130	-	CTX-M-24
SAMN16657684	ST23	KL1	O1/O2v2	Spain	2016	sputum	18	126	-	CTX-M-15
SAMN19700740	ST23	KL1	O1/O2v2	China	2017	Liver	4	129	-	-
SAMN19700744	ST23	KL1	O1/O2v2	China	2017	Liver	4	129	-	-
SAMN19700747	ST23	KL1	O1/O2v2	China	2018	Liver	4	129	-	-
SAMN19700746	ST23	KL1	O1/O2v2	China	2013	Liver	4	129	-	-
SAMN19700745	ST23	KL1	O1/O2v2	China	2014	Liver	4	129	-	-
SAMN19700742	ST23	KL1	O1/O2v2	China	2015	Liver	4	129	-	-

SAMN19700743	ST23	KL1	O1/O2v2	China	2017	Liver	4	129	-	-
SAMN19700741	ST23	KL1	O1/O2v2	China	2016	Liver	4	129	-	-
SAMN13563912	ST23	KL1	O1/O2v2	China	2014	Wound	4	128	-	-
SAMN13563913	ST23	KL1	O1/O2v2	China	2015	Wound	4	130	-	-
SAMN13563911	ST23	KL1	O1/O2v2	China	2014	Wound	6	130	-	CTX-M-14
SAMN13563909	ST23	KL1	O1/O2v2	China	2015	Wound	4	130	-	-
SAMN13563910	ST23	KL1	O1/O2v2	China	2015	Wound	4	130	-	-
SAMN13563905	ST23	KL1	O1/O2v2	China	2014	Wound	4	130	-	-
SAMN13563904	ST23	KL1	O1/O2v2	China	2014	Wound	4	130	-	-
SAMN13563896	ST23	KL1	O1/O2v2	China	2014	Wound	5	130	-	CTX-M-14
SAMN13563897	ST23	KL1	O1/O2v2	China	2014	Wound	4	130	-	-
SAMN13563892	ST23	KL1	O1/O2v2	China	2013	Wound	4	130	-	-
SAMN13563890	ST23	KL1	O1/O2v2	China	2013	Wound	4	130	-	-
SAMN13563902	ST23	KL1	O1/O2v2	China	2014	Wound	4	130	-	-
SAMN12349710	ST23	KL1	O1/O2v2	Germany	Unknown	Wound	9	128	OXA-48	-
SAMN20966673	ST23	KL1	O1/O2v2	China	2016	Unknown	6	130	IMP-4	-
SAMN20982458	ST23	KL1	O1/O2v2	Kazakhstan	2021	Throat	4	130	-	-
SAMN18874741	ST23	KL1	O1/O2v2	Jordan	2016	Unknown	16	118	NDM-1;OXA-48	CTX-M-14;CTX-M-15
SAMN19243880	ST23	KL57	O1/O2v2	Belarus	2020	sputum	15	72	OXA-48	CTX-M-15
SAMN16094139	ST23	KL1	O1/O2v2	China	2020	Blood	4	129	-	-
SAMN23002890	ST23	KL1	O1/O2v2	China	Unknown	Blood	4	130	-	-
SAMN23552373	ST23	KL1	O1/O2v2	Unknown	Unknown	Unknown	7	130	-	-
SAMN23552365	ST23	KL1	O1/O2v2	Unknown	Unknown	Unknown	7	130	-	-
SAMN15146843	ST23	KL1	O1/O2v2	Unknown	Unknown	Unknown	21	130	OXA-48	CTX-M-15
SAMN24562924	ST23	KL1	O1/O2v2	China	2020	sputum	10	130	-	CTX-M-15
SAMN24562925	ST23	KL1	O1/O2v2	China	2020	sputum	8	130	-	-
SAMN23898336	ST23	KL1	O1/O2v2	China	2020	sputum	10	130	-	CTX-M-71
SAMN24372389	ST23	KL1	O1/O2v2	Unknown	2021	Unknown	4	128	-	-
SAMN24108732	ST23	KL1	O1/O2v2	China	2019	sputum	6	129	KPC-2	-
SAMN24973877	ST23	KL1	O1/O2v2	India	2020	Blood	4	91	-	-
SAMN25608758	ST23	KL1	O1/O2v2	Russia	2012	Feces	5	128	-	-
SAMN25378372	ST23	KL1	O1/O2v2	China	2019	Blood	8	130	NDM-1	SHV-12
SAMN22567294	ST23	KL1	O1/O2v2	Unknown	2020	Environment	4	127	-	-
SAMN26018321	ST23	KL1	O1/O2v2	China	2019	Blood	4	128	-	-
SAMN26019433	ST23	KL1	O1/O2v2	China	2019	Blood	4	128	-	-

SAMN26020063	ST23	KL1	O1/O2v2	China	2019	Blood	20	129	-	CTX-M-3
SAMN26012836	ST23	KL1	O1/O2v2	China	2019	Blood	4	128	-	-
SAMN26012048	ST23	KL1	O1/O2v2	China	2019	Blood	4	128	-	-
SAMN26012945	ST23	KL1	O1/O2v2	China	2019	Blood	11	129	-	CTX-M-55
SAMN26012843	ST23	KL1	O1/O2v2	China	2019	Blood	4	129	-	-
SAMEA3357291	ST23	KL1	O1/O2v2	Australia	Unknown	Unknown	4	101	-	-
SAMN26498274	ST23	KL57	O1/O2v2	Poland	2019	Feces	17	84	OXA-48	CTX-M-55
SAMN26498273	ST23	KL57	O1/O2v2	Poland	2019	Aspirate	16	86	OXA-48	CTX-M-15
SAMN26498271	ST23	KL1	O1/O2v2	Poland	2019	Urine	17	130	VIM-1	SHV-12
SAMN26498272	ST23	KL57	O1/O2v2	Poland	2017	Feces	19	86	NDM-1	CTX-M-55
SAMN26498270	ST23	KL57	O1/O2v2	Poland	2015	Wound	18	86	OXA-48	CTX-M-55
SAMN26498268	ST23	KL57	O1/O2v2	Poland	2018	Urine	27	86	NDM-1	CTX-M-55
SAMN26498269	ST23	KL1	O1/O2v2	Poland	2018	Blood	7	129	KPC-2	-
SAMN26498267	ST23	KL57	O1/O2v2	Poland	2018	Feces	21	85	KPC-2	CTX-M-55
SAMN26498266	ST23	KL57	O1/O2v2	Poland	2018	Feces	31	85	KPC-2;NDM-1	CTX-M-55
SAMN26498265	ST23	KL57	O1/O2v2	Poland	2018	Feces	31	85	KPC-2;NDM-1	CTX-M-55
SAMN26498260	ST23	KL57	O1/O2v2	Poland	2018	Aspirate	30	86	KPC-2;NDM-1	CTX-M-55
SAMN26498262	ST23	KL57	O1/O2v2	Poland	2018	Feces	29	93	KPC-2;NDM-1	CTX-M-55
SAMN26498261	ST23	KL57	O1/O2v2	Poland	2018	Feces	30	94	KPC-2;NDM-1	CTX-M-55
SAMN26750072	ST23	KL1	O1/O2v2	Belgium	2014	Blood	4	129	-	-
SAMN26750074	ST23	KL1	O1/O2v2	Belgium	2014	Blood	4	129	-	-
SAMN26750087	ST23	KL1	O1/O2v2	Belgium	2019	Liver	4	128	-	-
SAMN26750085	ST23	KL1	O1/O2v2	Belgium	2019	Blood	5	128	-	-
SAMN26750080	ST23	KL1	O1/O2v2	Belgium	2017	Blood	4	129	-	-
SAMN26750076	ST23	KL1	O1/O2v2	Belgium	2016	Eye	4	129	-	-
SAMN26750071	ST23	KL1	O1/O2v2	Belgium	2014	Blood	4	123	-	-
SAMN27593784	ST23	KL1	O1/O2v2	China	2022	Unknown	4	120	-	-
SAMN28187128	ST23	KL1	O1/O2v2	China	2021	Abscess	4	130	-	-
SAMN25039377	ST23	KL1	O1/O2v2	India	2019	Urine	4	128	-	-
SAMN28447209	ST23	KL1	O1/O2v2	India	2017	Urine	4	101	-	-
SAMN28447051	ST23	KL1	O1/O2v2	India	2017	sputum	4	127	-	-
SAMN28447312	ST23	KL1	O1/O2v2	India	2017	sputum	4	101	-	-
SAMN26816214	ST23	KL1	O1/O2v2	India	2020	Aspirate	4	101	-	-
SAMN26554414	ST23	KL1	O1/O2v2	Italy	2021	Blood	4	129	-	-
SAMN26554415	ST23	KL1	O1/O2v2	Italy	2021	Lung	4	128	-	-

SAMN24814241	ST23	KL1	O1/O2v2	China	2018	Feces	4	129	-	-
SAMN24814228	ST23	KL1	O1/O2v2	China	2018	Feces	4	129	-	-
SAMN24814189	ST23	KL1	O1/O2v2	China	2018	Feces	4	130	-	-
SAMN17073120	ST23	KL1	O1/O2v2	China	2019	Animal	4	130	-	-
SAMN17073122	ST23	KL1	O1/O2v2	China	2019	Animal	4	130	-	-
SAMN17073097	ST23	KL1	O1/O2v2	China	2018	Animal	4	130	-	-
SAMN17073108	ST23	KL1	O1/O2v2	China	2017	Animal	4	130	-	-
SAMN17073130	ST23	KL1	O1/O2v2	China	2017	Animal	4	130	-	-
SAMN30252885	ST23	KL1	O1/O2v2	Unknown	2022	Unknown	6	130	KPC-2	-
SAMN30971612	ST23	KL107	O1/O2v1	Russia	2022	Blood	15	81	OXA-48	CTX-M-15
SAMN31116560	ST23	KL1	O1/O2v2	Unknown	2022	Blood	6	124	-	CTX-M-3
SAMN31116561	ST23	KL1	O1/O2v2	Unknown	2022	Vagina	6	130	-	CTX-M-3
SAMN28824987	ST23	KL1	O1/O2v2	China	2016	Environment	5	128	-	CTX-M-14
SAMD00406251	ST23	KL1	O1/O2v2	Japan	2020	Unknown	4	125	-	-
SAMD00406252	ST23	KL1	O1/O2v2	Japan	2020	Unknown	4	125	-	-
SAMD00406253	ST23	KL1	O1/O2v2	Japan	2020	Unknown	4	125	-	-
SAMN28824994	ST23	KL1	O1/O2v2	China	2016	Abscess	4	128	-	-
SAMN31116812	ST23	KL1	O1/O2v2	China	2018	Unknown	16	129	KPC-2	CTX-M-14;CTX-M-15
SAMN31116570	ST23	KL1	O1/O2v2	China	2018	Unknown	17	130	KPC-2	CTX-M-14;CTX-M-15
SAMN31116564	ST23	KL1	O1/O2v2	China	2018	Unknown	17	130	KPC-2	CTX-M-14;CTX-M-15
SAMN31113471	ST23	KL1	O1/O2v2	China	2018	Unknown	17	129	KPC-2	CTX-M-14;CTX-M-15
SAMN31113466	ST23	KL1	O1/O2v2	China	2018	Unknown	16	129	KPC-2	CTX-M-14;CTX-M-15
SAMN31113404	ST23	KL1	O1/O2v2	China	2018	Unknown	16	129	KPC-2	CTX-M-15
SAMN31113402	ST23	KL1	O1/O2v2	China	2018	Unknown	15	128	KPC-2	CTX-M-15
SAMN31113465	ST23	KL1	O1/O2v2	China	2018	Unknown	14	129	KPC-2	CTX-M-15
SAMN16177880	ST23	KL1	O1/O2v2	India	2019	Aspirate	4	130	-	-
SAMN28408403	ST23	KL1	O1/O2v2	China	2021	Unknown	4	128	-	-
SAMN28408383	ST23	KL1	O1/O2v2	China	2021	Unknown	4	128	-	-
SAMN28408363	ST23	KL1	O1/O2v2	China	2020	Unknown	4	128	-	-
SAMN28408355	ST23	KL1	O1/O2v2	China	2020	Unknown	4	128	-	-

SAMN28408351	ST23	KL1	O1/O2v2	China	2020	Unknown	4	128	-	-
SAMN28408333	ST23	KL1	O1/O2v2	China	2020	Unknown	4	128	-	-
SAMN28408330	ST23	KL1	O1/O2v2	China	2020	Unknown	4	130	-	-
SAMN28408339	ST23	KL1	O1/O2v2	China	2020	Unknown	4	128	-	-
SAMN28408316	ST23	KL1	O1/O2v2	China	2020	Unknown	7	129	-	CTX-M-3
SAMN28950853	ST23	KL1	O1/O2v2	China	2017	Feces	4	130	-	-
SAMN28950850	ST23	KL1	O1/O2v2	China	2017	Feces	4	124	-	-
SAMN31880353	ST23	KL1	O1/O2v2	China	2019	Liver	4	128	-	-
SAMN31880384	ST23	KL1	O1/O2v2	China	2021	Bile	4	129	-	-
SAMN31880385	ST23	KL1	O1/O2v2	China	2020	Bile	4	129	-	-
SAMN31880378	ST23	KL1	O1/O2v2	China	2019	Blood	4	128	-	-
SAMN31880383	ST23	KL1	O1/O2v2	China	2021	Blood	4	128	-	-
SAMN31880381	ST23	KL1	O1/O2v2	China	2019	Blood	4	128	-	-
SAMN31880368	ST23	KL1	O1/O2v2	China	2019	Bile	4	128	-	-
SAMN31880361	ST23	KL1	O1/O2v2	China	2019	sputum	7	128	-	TEM-116*
SAMN31880373	ST23	KL1	O1/O2v2	China	2021	Blood	4	128	-	-
SAMN31880365	ST23	KL1	O1/O2v2	China	2018	Blood	4	128	-	-
SAMN31880366	ST23	KL1	O1/O2v2	China	2019	Liver	4	128	-	-
SAMN31880372	ST23	KL1	O1/O2v2	China	2021	sputum	4	128	-	-
SAMN31880362	ST23	KL1	O1/O2v2	China	2019	Blood	4	128	-	-
SAMN31880379	ST23	KL1	O1/O2v2	China	2019	Liver	4	128	-	-
SAMN33279463	ST23	KL1	O1/O2v2	Japan	2022	Heart	4	127	-	-
SAMN33420516	ST23	KL1	O1/O2v2	China	2022	Unknown	6	112	NDM-1	-
SAMN33610088	ST23	KL1	O1/O2v2	Chile	2022	Aspirate	7	130	KPC-2	-
SAMN26241722	ST23	KL1	O1/O2v2	China	2018	Blood	9	129	-	CTX-M-55
SAMN26241716	ST23	KL1	O1/O2v2	China	2018	Blood	4	129	-	-
SAMN26241711	ST23	KL1	O1/O2v2	China	2018	Blood	4	129	-	-
SAMN26241698	ST23	KL1	O1/O2v2	China	2020	Pleural Effusion	4	129	-	-
SAMN26241697	ST23	KL1	O1/O2v2	China	2020	Abscess	4	129	-	-
SAMN26241696	ST23	KL1	O1/O2v2	China	2020	Abscess	4	129	-	-
SAMN26241691	ST23	KL1	O1/O2v2	China	2020	Abscess	4	129	-	-
SAMN26241670	ST23	KL1	O1/O2v2	China	2019	Blood	4	129	-	-
SAMN26241669	ST23	KL1	O1/O2v2	China	2019	Blood	4	129	-	-
SAMN26241667	ST23	KL1	O1/O2v2	China	2019	Blood	4	129	-	-
SAMN26241668	ST23	KL1	O1/O2v2	China	2019	Blood	4	118	-	-
SAMN26241666	ST23	KL1	O1/O2v2	China	2019	Abscess	4	128	-	-
SAMN26241664	ST23	KL1	O1/O2v2	China	2019	Abscess	4	100	-	-

SAMN26241665	ST23	KL1	O1/O2v2	China	2019	Abscess	4	124	-	-
SAMN26241662	ST23	KL1	O1/O2v2	China	2019	Abscess	4	129	-	-
SAMN26241663	ST23	KL1	O1/O2v2	China	2019	Lung	4	129	-	-
SAMN33059483	ST23	KL1	O1/O2v2	China	2022	Blood	4	130	-	-
SAMN33059473	ST23	KL1	O1/O2v2	China	2022	Blood	4	130	-	-
SAMN33059467	ST23	KL1	O1/O2v2	China	2022	sputum	4	130	-	-
SAMN33059466	ST23	KL1	O1/O2v2	China	2022	Environment	4	130	-	-
SAMN33059464	ST23	KL1	O1/O2v2	China	2022	Environment	4	130	-	-
SAMN28546459	ST23	KL1	O1/O2v2	China	Unknown	Unknown	10	129	-	CTX-M-71
SAMN28546420	ST23	KL1	O1/O2v2	China	Unknown	Unknown	6	129	NDM-1	-
SAMN35028424	ST23	KL1	O1/O2v2	China	2022	sputum	20	130	-	CTX-M-3
SAMN35028419	ST23	KL1	O1/O2v2	China	2022	sputum	9	130	-	CTX-M-15
SAMN35028416	ST23	KL1	O1/O2v2	China	2022	sputum	7	131	-	CTX-M-55
SAMN35028415	ST23	KL1	O1/O2v2	China	2022	sputum	9	130	-	CTX-M-15
SAMN35028409	ST23	KL1	O1/O2v2	China	2022	sputum	9	130	-	CTX-M-15
SAMEA3531794	ST23	KL1	O1/O2v2	Thailand	2015	Blood	6	129	-	CTX-M-55
SAMN35995167	ST23	KL1	O1/O2v2	USA	2021	sputum	4	130	-	-
SAMEA114122464	ST23	KL1	O1/O2v2	China	Unknown	Feces	4	119	-	-
SAMN04532005	ST23	KL1	O1/O2v2	USA	2015	Bone	5	117	-	-
SAMN05425525	ST23	KL1	O1/O2v2	Taiwan	2006	Blood	4	120	-	-
SAMN10908546	ST23	KL1	O1/O2v2	South Korea	2017	Abscess	5	130	KPC-2	-
SAMN36766149	ST23	KL1	O1/O2v2	China	2019	Abscess	4	120	-	-
SAMN36766156	ST23	KL1	O1/O2v2	China	2016	sputum	4	120	-	-
SAMN36735522	ST23	KL1	O1/O2v2	Oman	2021	Unknown	4	128	-	-
SAMN36735518	ST23	KL1	O1/O2v2	Oman	2021	Unknown	8	122	-	CTX-M-15
SAMN36071315	ST23	KL1	O1/O2v2	Ukraine	2014	Sputum	4	130	-	-
SAMN36071313	ST23	KL1	O1/O2v2	USA	2014	Urine	4	129	-	-
SAMN36071311	ST23	KL1	O1/O2v2	USA	2016	Urine	14	130	-	CTX-M-15
SAMN36071310	ST23	KL1	O1/O2v2	USA	2012	Sputum	4	130	-	-
SAMN36071308	ST23	KL1	O1/O2v2	USA	2012	Blood	4	130	-	-
SAMN36071293	ST23	KL1	O1/O2v2	USA	2014	Urine	5	130	-	-
SAMN37270754	ST23	KL1	O1/O2v2	China	2021	Unknown	4	128	-	-
SAMN29340047	ST23	KL1	O1/O2v2	India	2021	Blood	4	128	-	-
SAMN29340049	ST23	KL1	O1/O2v2	India	2020	Blood	4	125	-	-
SAMN29340050	ST23	KL1	O1/O2v2	India	2020	Blood	6	128	-	-
SAMN37513143	ST23	KL1	O1/O2v2	Chile	2022	Aspirate	13	130	KPC-2;VIM-1	-

SAMN30170512	ST23	KL1	O1/O2v2	India	2021	Blood	4	127	-	-
SAMN37686350	ST23	KL1	O1/O2v2	Hungary	2023	Wound	4	130	-	-
SAMN37985424	ST23	KL1	O1/O2v2	Mexico	2023	Liver	4	128	-	-
SAMN38757442	ST23	KL1	O1/O2v2	China	2021	Abscess	12	131	-	CTX-M-3
SAMN34004029	ST23	KL1	O1/O2v2	Italy	2021	Unknown	4	129	-	-
SAMN39556033	ST23	KL57	O1/O2v2	Russia	2021	Throat	17	85	-	CTX-M-55
SAMN33040085	ST23	KL1	O1/O2v2	China	2019	Blood	4	130	-	-
SAMN33040086	ST23	KL1	O1/O2v2	China	2019	Unknown	4	130	-	-
SAMN33040082	ST23	KL1	O1/O2v2	China	2019	Blood	4	130	-	-
SAMN33040074	ST23	KL1	O1/O2v2	China	2018	Blood	4	130	-	-
SAMN33040072	ST23	KL1	O1/O2v2	China	2018	Blood	4	125	-	-
SAMN33040075	ST23	KL1	O1/O2v2	China	2018	Unknown	4	130	-	-
SAMN39714597	ST23	KL1	O1/O2v2	China	2019	Environment	4	130	-	-
SAMN39714598	ST23	KL1	O1/O2v2	China	2019	sputum	4	130	-	-
SAMEA8948741	ST23	KL1	O1/O2v2	Norway	2015	Unknown	4	129	-	-
SAMEA10468409	ST23	KL1	O1/O2v2	Ireland	Unknown	Unknown	12	130	OXA-48	-
SAMEA10468441	ST23	KL1	O1/O2v2	Ireland	Unknown	Unknown	12	130	OXA-48	-
SAMEA10468500	ST23	KL1	O1/O2v2	Ireland	Unknown	Unknown	12	130	OXA-48	-
SAMEA10468574	ST23	KL1	O1/O2v2	Ireland	Unknown	Unknown	12	130	OXA-48	-
SAMEA10468588	ST23	KL1	O1/O2v2	Ireland	Unknown	Unknown	12	130	OXA-48	-
SAMN33790395	ST23	KL57	O1/O2v2	Russia	2022	Unknown	19	85	-	CTX-M-55
SAMN40563566	ST23	KL1	O1/O2v2	Ireland	2019	Feces	5	128	OXA-48	-
SAMN40563565	ST23	KL1	O1/O2v2	Ireland	2019	Feces	12	125	OXA-48	-
SAMN40563581	ST23	KL1	O1/O2v2	Ireland	2020	Urine	5	128	OXA-48	-
SAMN40563579	ST23	KL1	O1/O2v2	Ireland	2019	Feces	16	127	OXA-48	CTX-M-15
SAMN40563578	ST23	KL1	O1/O2v2	Ireland	2019	Feces	16	128	OXA-48	CTX-M-15
SAMN40563582	ST23	KL1	O1/O2v2	Ireland	2020	Blood	15	130	-	CTX-M-15
SAMN40563577	ST23	KL1	O1/O2v2	Ireland	2019	Feces	16	127	OXA-48	CTX-M-15
SAMN40563583	ST23	KL1	O1/O2v2	Ireland	2020	Blood	5	128	OXA-48	-
SAMN40563564	ST23	KL1	O1/O2v2	Ireland	2019	Feces	16	127	OXA-48	CTX-M-15
SAMN40563580	ST23	KL1	O1/O2v2	Ireland	2020	Environment	12	128	OXA-48	-
SAMN40563562	ST23	KL1	O1/O2v2	Ireland	2019	Feces	12	127	OXA-48	-
SAMN40563574	ST23	KL1	O1/O2v2	Ireland	2019	Feces	16	128	OXA-48	CTX-M-15
SAMN40563561	ST23	KL1	O1/O2v2	Ireland	2019	Feces	17	128	OXA-48	CTX-M-15
SAMN40563560	ST23	KL1	O1/O2v2	Ireland	2019	Feces	5	128	OXA-48	-
SAMN40563576	ST23	KL1	O1/O2v2	Ireland	2019	Feces	16	128	OXA-48	CTX-M-15
SAMN40563575	ST23	KL1	O1/O2v2	Ireland	2019	Urine	11	126	OXA-48	-

SAMN40563572	ST23	KL1	O1/O2v2	Ireland	2019	Foot	16	127	OXA-48	CTX-M-15
SAMN40563571	ST23	KL1	O1/O2v2	Ireland	2019	Feces	5	127	OXA-48	-
SAMN40563573	ST23	KL1	O1/O2v2	Ireland	2019	Feces	12	128	OXA-48	-
SAMN40563570	ST23	KL1	O1/O2v2	Ireland	2019	Feces	5	128	OXA-48	-
SAMN40563568	ST23	KL1	O1/O2v2	Ireland	2019	Feces	11	128	OXA-48	-
SAMN40563563	ST23	KL1	O1/O2v2	Ireland	2019	Blood	16	128	-	CTX-M-15
SAMN40563569	ST23	KL1	O1/O2v2	Ireland	2019	Feces	12	128	OXA-48	-
SAMN40563567	ST23	KL1	O1/O2v2	Ireland	2019	Urine	11	126	OXA-48	-
SAMN40563637	ST23	KL57	O1/O2v2	Ireland	2023	Feces	20	86	NDM-1	-
SAMN40563632	ST23	KL1	O1/O2v2	Ireland	2022	Feces	5	128	OXA-48	-
SAMN40563636	ST23	KL1	O1/O2v2	Ireland	2023	Blood	11	128	-	-
SAMN40563616	ST23	KL1	O1/O2v2	Ireland	2022	Feces	5	128	OXA-48	-
SAMN40563640	ST23	KL1	O1/O2v2	Ireland	2023	Blood	4	128	-	-
SAMN40563634	ST23	KL1	O1/O2v2	Ireland	2022	Blood	10	128	-	-
SAMN40563625	ST23	KL57	O1/O2v2	Ireland	2022	Feces	23	86	-	CTX-M-55
SAMN40563631	ST23	KL1	O1/O2v2	Ireland	2022	Feces	12	128	OXA-48	-
SAMN40563610	ST23	KL1	O1/O2v2	Ireland	2022	Feces	11	128	OXA-48	-
SAMN40563623	ST23	KL1	O1/O2v2	Ireland	2022	Feces	10	128	OXA-48	-
SAMN40563645	ST23	KL1	O1/O2v2	Ireland	2023	Feces	5	128	OXA-48	-
SAMN40563621	ST23	KL1	O1/O2v2	Ireland	2022	Blood	16	128	-	CTX-M-15
SAMN40563635	ST23	KL1	O1/O2v2	Ireland	2023	Feces	6	128	OXA-48	-
SAMN40563629	ST23	KL1	O1/O2v2	Ireland	2022	Feces	11	128	OXA-48	-
SAMN40563628	ST23	KL1	O1/O2v2	Ireland	2022	Urine	5	128	OXA-48	-
SAMN40563617	ST23	KL1	O1/O2v2	Ireland	2022	Feces	5	128	OXA-48	-
SAMN40563604	ST23	KL1	O1/O2v2	Ireland	2021	Blood	5	128	-	-
SAMN40563624	ST23	KL1	O1/O2v2	Ireland	2022	Eye	4	128	-	-
SAMN40563614	ST23	KL1	O1/O2v2	Ireland	2022	Feces	5	128	OXA-48	-
SAMN40563613	ST23	KL1	O1/O2v2	Ireland	2022	Feces	6	128	OXA-48	-
SAMN40563609	ST23	KL1	O1/O2v2	Ireland	2022	Blood	10	128	-	-
SAMN40563588	ST23	KL1	O1/O2v2	Ireland	2020	Urine	6	128	OXA-48	-
SAMN40563605	ST23	KL1	O1/O2v2	Ireland	2021	Feces	5	128	OXA-48	-
SAMN40563592	ST23	KL1	O1/O2v2	Ireland	2020	Throat	5	128	OXA-48	-
SAMN40563602	ST23	KL1	O1/O2v2	Ireland	2021	Feces	6	128	OXA-48	-
SAMN40563599	ST23	KL1	O1/O2v2	Ireland	2020	Urine	5	129	OXA-48	-
SAMN40563603	ST23	KL1	O1/O2v2	Ireland	2021	Blood	4	128	-	-
SAMN40563612	ST23	KL1	O1/O2v2	Ireland	2022	Feces	5	128	OXA-48	-
SAMN40563598	ST23	KL1	O1/O2v2	Ireland	2020	Feces	12	128	OXA-48	-

SAMN40563611	ST23	KL1	O1/O2v2	Ireland	2022	Blood	5	128	OXA-48	-
SAMN40563596	ST23	KL1	O1/O2v2	Ireland	2020	Feces	5	124	OXA-48	-
SAMN40563607	ST23	KL1	O1/O2v2	Ireland	2022	Feces	5	130	OXA-48	-
SAMN40563606	ST23	KL1	O1/O2v2	Ireland	2021	Feces	10	128	OXA-48	-
SAMN40563593	ST23	KL1	O1/O2v2	Ireland	2020	Feces	5	128	OXA-48	-
SAMN40563601	ST23	KL1	O1/O2v2	Ireland	2021	Bile	4	127	-	-
SAMN40563597	ST23	KL1	O1/O2v2	Ireland	2020	Blood	4	128	-	-
SAMN40563600	ST23	KL1	O1/O2v2	Ireland	2021	Blood	10	128	OXA-48	-
SAMN40563594	ST23	KL1	O1/O2v2	Ireland	2020	Feces	5	123	OXA-48	-
SAMN40563627	ST23	KL1	O1/O2v2	Ireland	2022	Feces	6	128	OXA-48	-
SAMN40563649	ST23	KL1	O1/O2v2	Ireland	2023	Urine	6	127	OXA-48	-
SAMN40563622	ST23	KL1	O1/O2v2	Ireland	2022	Feces	5	128	OXA-48	-
SAMN40563586	ST23	KL1	O1/O2v2	Ireland	2020	Feces	6	128	OXA-48	-
SAMN40563646	ST23	KL1	O1/O2v2	Ireland	2023	Feces	5	128	OXA-48	-
SAMN40563591	ST23	KL1	O1/O2v2	Ireland	2020	Feces	6	128	OXA-48	-
SAMN40563595	ST23	KL1	O1/O2v2	Ireland	2020	Feces	12	127	OXA-48	-
SAMN40563590	ST23	KL1	O1/O2v2	Ireland	2020	Feces	5	128	OXA-48	-
SAMN40563587	ST23	KL1	O1/O2v2	Ireland	2020	Feces	6	128	OXA-48	-
SAMN40563589	ST23	KL1	O1/O2v2	Ireland	2020	Liver	4	128	-	-
SAMN40563585	ST23	KL1	O1/O2v2	Ireland	2020	Feces	12	128	OXA-48	-
SAMN40563584	ST23	KL1	O1/O2v2	Ireland	2020	Liver	4	128	-	-
SAMN40563618	ST23	KL1	O1/O2v2	Ireland	2022	Environment	10	128	OXA-48	-
SAMN40563619	ST23	KL1	O1/O2v2	Ireland	2022	Feces	5	128	OXA-48	-
SAMN40563608	ST23	KL1	O1/O2v2	Ireland	2022	Feces	11	128	OXA-48	-
SAMN40563630	ST23	KL1	O1/O2v2	Ireland	2022	Feces	12	127	OXA-48	-
SAMN40563620	ST23	KL1	O1/O2v2	Ireland	2022	Feces	11	128	OXA-48	-
SAMN40563641	ST23	KL1	O1/O2v2	Ireland	2023	Feces	5	127	OXA-48	-
SAMN40563648	ST23	KL1	O1/O2v2	Ireland	2023	Feces	5	128	OXA-48	-
SAMN40563626	ST23	KL1	O1/O2v2	Ireland	2022	sputum	6	128	OXA-48	-
SAMN40563615	ST23	KL1	O1/O2v2	Ireland	2022	Feces	5	128	OXA-48	-
SAMN40563643	ST23	KL1	O1/O2v2	Ireland	2023	Feces	6	128	OXA-48	-
SAMN40563644	ST23	KL1	O1/O2v2	Ireland	2023	Urine	15	130	OXA-181	CTX-M-15
SAMN40563647	ST23	KL1	O1/O2v2	Ireland	2023	Feces	6	128	OXA-48	-
SAMN40563638	ST23	KL1	O1/O2v2	Ireland	2023	Feces	5	128	OXA-48	-
SAMN40563633	ST23	KL1	O1/O2v2	Ireland	2022	Blood	4	128	-	-
SAMN40563639	ST23	KL1	O1/O2v2	Ireland	2023	Feces	5	128	OXA-48	-
SAMN40579959	ST23	KL1	O1/O2v2	Ukraine	2023	Wound	4	128	-	-

SAMN05425526	ST23	KL1	O1/O2v2	Taiwan	2006	Blood	4	112	-	-
SAMN05413580	ST23	KL1	O1/O2v2	Unknown	2014	Unknown	11	130	-	CTX-M-15
SAMN05412805	ST23	KL57	O1/O2v2	Germany	2014	Wound	18	86	OXA-48	-
SAMN40563642	ST23	KL1	O1/O2v2	Ireland	2023	Feces	5	128	OXA-48	-
SAMN40181871	ST23	KL1	O1/O2v2	China	2023	Unknown	4	128	-	-
SAMN40181865	ST23	KL1	O1/O2v2	China	2023	Unknown	4	128	-	-
SAMN40181858	ST23	KL1	O1/O2v2	China	2023	Unknown	4	128	-	-
SAMN40181844	ST23	KL1	O1/O2v2	China	2022	Unknown	3	98	-	-
SAMN40181846	ST23	KL1	O1/O2v2	China	2022	Unknown	4	128	-	-
SAMN40181843	ST23	KL1	O1/O2v2	China	2022	Unknown	4	128	-	-
SAMN40181839	ST23	KL1	O1/O2v2	China	2022	Unknown	4	128	-	-
SAMN40181833	ST23	KL1	O1/O2v2	China	2022	Unknown	4	119	-	-
SAMN40181830	ST23	KL1	O1/O2v2	China	2022	Unknown	4	128	-	-
SAMN40181828	ST23	KL1	O1/O2v2	China	2022	Unknown	4	128	-	-
SAMN40181824	ST23	KL1	O1/O2v2	China	2021	Unknown	4	128	-	-
SAMN40181822	ST23	KL1	O1/O2v2	China	2021	Unknown	4	127	-	-
SAMN40181793	ST23	KL1	O1/O2v2	China	2020	Unknown	4	121	-	-
SAMN40181786	ST23	KL1	O1/O2v2	China	2020	Unknown	4	128	-	-
SAMN40181778	ST23	KL1	O1/O2v2	China	2020	Unknown	4	128	-	-
SAMN40181775	ST23	KL1	O1/O2v2	China	2020	Unknown	4	128	-	-
SAMN40755494	ST23	KL1	O1/O2v2	Russia	2023	Lung	4	128	-	-
SAMN40981038	ST23	KL1	O1/O2v2	China	2023	Unknown	7	130	NDM-1	SHV-12
SAMN40981037	ST23	KL1	O1/O2v2	China	2023	Unknown	7	130	NDM-1	SHV-12
SAMN40981036	ST23	KL1	O1/O2v2	China	2023	Unknown	7	130	NDM-1	SHV-12
SAMN41002031	ST23	KL1	O1/O2v2	China	2021	Liver	19	131	-	-
SAMN41028361	ST23	KL1	O1/O2v2	China	2023	Unknown	4	107	-	-
SAMN41018409	ST23	KL1	O1/O2v2	USA	2012	Blood	4	130	-	-
SAMN40991831	ST23	KL1	O1/O2v2	China	2023	sputum	6	126	KPC-44	-
SAMN05928591	ST23	KL1	O1/O2v2	China	2015	Liver	4	130	-	-
SAMN04521860	ST23	KL1	O1/O2v2	USA	2015	Unknown	4	119	-	-
SAMN04377968	ST23	KL1	O1/O2v2	China	2015	Blood	4	130	-	-
SAMN10523731	ST23	KL1	O1/O2v2	Unknown	Unknown	Unknown	8	101	-	-
SAMN13503956	ST23	KL1	O1/O2v2	South Korea	2017	Feces	4	130	-	-
SAMN13503954	ST23	KL1	O1/O2v2	South Korea	2017	Liver	4	130	-	-
SAMN12740646	ST23	KL107	OL102	Madagascar	2017	sputum	4	99	-	-
SAMN18344183	ST23	KL1	O1/O2v2	Unknown	Unknown	Unknown	8	125	KPC-2	-
SAMN33948602	ST23	KL1	O1/O2v2	Chile	2020	Aspirate	4	130	-	-

SAMN34258171	ST23	KL1	O1/O2v2	United Kingdom	2022	Blood	4	130	-	-
SAMD00500794	ST23	KL1	O1/O2v2	Japan	Unknown	Unknown	11	128	IMP-6	CTX-M-2
SAMN06562536	ST23	KL1	O1/O2v2	United Kingdom	2016	Feces	5	130	OXA-48;OXA-48	-
SAMN27753784	ST23	KL1	O1/O2v2	China	2017	Unknown	5	130	KPC-2	-
SAMN27753785	ST23	KL1	O1/O2v2	China	2017	Unknown	15	129	KPC-2	CTX-M-3
SAMN27753786	ST23	KL1	O1/O2v2	China	2017	Unknown	6	130	KPC-2	-
SAMN27753787	ST23	KL1	O1/O2v2	China	2017	Unknown	8	130	KPC-2	SHV-12
SAMN46080740	ST23	KL1	O1/O2v2	China	2014	Blood	7	130	NDM-1	SHV-12
SAMN46080741	ST23	KL1	O1/O2v2	China	2019	Blood	5	120	KPC-2	-
SAMN46080743	ST23	KL1	O1/O2v2	China	2019	Blood	15	130	NDM-1	CTX-M-14
SAMN46080742	ST23	KL1	O1/O2v2	China	2020	Blood	4	130	-	-
SAMN46080744	ST23	KL1	O1/O2v2	China	2020	Blood	17	130	OXA-48	CTX-M-15
SAMN46080745	ST23	KL1	O1/O2v2	China	2021	Blood	5	130	KPC-2	-
SAMN46080746	ST23	KL1	O1/O2v2	China	2021	Blood	14	130	NDM-1;NDM-1	CTX-M-15;SHV-12
SAMN46080747	ST23	KL1	O1/O2v2	China	2021	Blood	13	130	NDM-1	CTX-M-15
SAMN46080748	ST23	KL1	O1/O2v2	China	2023	Blood	6	130	KPC-2	-
SAMN46080749	ST23	KL1	O1/O2v2	China	2023	Blood	5	130	KPC-2	-
SAMN53233793	ST23	KL1	O1/O2v2	China	2020	Blood	4	130	-	-
SAMN53233819	ST23	KL1	O1/O2v2	China	2014	Blood	4	130	-	-
SAMN53233860	ST23	KL1	O1/O2v2	China	2018	Blood	10	130	-	-
SAMN53233871	ST23	KL1	O1/O2v2	China	2018	Blood	4	130	-	-
SAMN53233881	ST23	KL1	O1/O2v2	China	2018	Blood	4	130	-	-
SAMN53233798	ST23	KL1	O1/O2v2	China	2022	Blood	4	130	-	-
SAMN53233799	ST23	KL1	O1/O2v2	China	2022	Blood	20	131	-	CTX-M-3
SAMN53233800	ST23	KL1	O1/O2v2	China	2023	Blood	4	130	-	-
SAMN53233784	ST23-1LV	KL1	O1/O2v2	China	2013	Liver	4	130	-	-
SAMN53233785	ST23-1LV	KL1	O1/O2v2	China	2013	Liver	4	130	-	-
SAMN53233786	ST23	KL1	O1/O2v2	China	2013	Liver	4	130	-	-
SAMN53233787	ST23	KL1	O1/O2v2	China	2013	Liver	4	130	-	-
SAMN53233788	ST23	KL1	O1/O2v2	China	2013	Liver	4	122	-	-
SAMN53233789	ST23	KL1	O1/O2v2	China	2013	Liver	4	130	-	-
SAMN53233790	ST23	KL1	O1/O2v2	China	2013	Liver	4	130	-	-
SAMN53233791	ST23	KL1	O1/O2v2	China	2013	Liver	4	125	-	-
SAMN53233792	ST23	KL1	O1/O2v2	China	2013	Liver	4	130	-	-
SAMN53233794	ST23	KL1	O1/O2v2	China	2014	Liver	4	130	-	-

SAMN53233795	ST23	KL1	O1/O2v2	China	2014	Liver	4	130	-	-
SAMN53233796	ST23	KL1	O1/O2v2	China	2014	Liver	4	130	-	-
SAMN53233797	ST23	KL1	O1/O2v2	China	2014	Liver	4	130	-	-
SAMN53233801	ST23	KL1	O1/O2v2	China	2014	Liver	4	130	-	-
SAMN53233802	ST23	KL1	O1/O2v2	China	2014	Liver	4	130	-	-
SAMN53233803	ST23	KL1	O1/O2v2	China	2014	Liver	4	130	-	-
SAMN53233804	ST23	KL1	O1/O2v2	China	2014	Liver	4	130	-	-
SAMN53233805	ST23	KL1	O1/O2v2	China	2014	Liver	4	130	-	-
SAMN53233806	ST23	KL1	O1/O2v2	China	2014	Liver	4	130	-	-
SAMN53233807	ST23	KL1	O1/O2v2	China	2015	Liver	4	130	-	-
SAMN53233808	ST23	KL1	O1/O2v2	China	2015	Liver	4	130	-	-
SAMN53233809	ST23	KL1	O1/O2v2	China	2015	Liver	4	130	-	-
SAMN53233810	ST23	KL1	O1/O2v2	China	2015	Liver	4	109	-	-
SAMN53233811	ST23	KL1	O1/O2v2	China	2015	Liver	4	130	-	-
SAMN53233812	ST23	KL1	O1/O2v2	China	2015	Liver	4	130	-	-
SAMN53233813	ST23	KL1	O1/O2v2	China	2015	Liver	4	130	-	-
SAMN53233814	ST23	KL1	O1/O2v2	China	2015	Liver	4	130	-	-
SAMN53233815	ST23	KL1	O1/O2v2	China	2015	Liver	4	128	-	-
SAMN53233816	ST23	KL1	O1/O2v2	China	2015	Liver	4	130	-	-
SAMN53233817	ST23	KL1	O1/O2v2	China	2015	Liver	4	130	-	-
SAMN53233818	ST23	KL1	O1/O2v2	China	2015	Liver	4	129	-	-
SAMN53233820	ST23	KL1	O1/O2v2	China	2015	Liver	4	130	-	-
SAMN53233821	ST23	KL1	O1/O2v2	China	2015	Liver	4	130	-	-
SAMN53233822	ST23	KL1	O1/O2v2	China	2015	Liver	4	130	-	-
SAMN53233823	ST23	KL1	O1/O2v2	China	2016	Liver	11	130	-	-
SAMN53233824	ST23	KL1	O1/O2v2	China	2016	Liver	4	130	-	-
SAMN53233825	ST23	KL1	O1/O2v2	China	2016	Liver	4	130	-	-
SAMN53233826	ST23	KL1	O1/O2v2	China	2016	Liver	4	130	-	-
SAMN53233827	ST23	KL1	O1/O2v2	China	2016	Liver	4	130	-	-
SAMN53233828	ST23	KL1	O1/O2v2	China	2016	Liver	4	130	-	-
SAMN53233829	ST23	KL1	O1/O2v2	China	2016	Liver	4	130	-	-
SAMN53233830	ST23	KL1	O1/O2v2	China	2016	Liver	4	130	-	-
SAMN53233831	ST23	KL1	O1/O2v2	China	2016	Liver	4	130	-	-
SAMN53233832	ST23	KL1	O1/O2v2	China	2016	Liver	4	130	-	-
SAMN53233833	ST23	KL1	O1/O2v2	China	2016	Liver	4	130	-	-
SAMN53233834	ST23	KL1	O1/O2v1	China	2016	Liver	4	117	-	-
SAMN53233835	ST23	KL1	O1/O2v2	China	2016	Liver	4	130	-	-

SAMN53233836	ST23	KL1	O1/O2v2	China	2016	Liver	4	130	-	-
SAMN53233837	ST23	KL1	O1/O2v2	China	2016	Liver	4	125	-	-
SAMN53233838	ST23	KL1	O1/O2v2	China	2016	Liver	4	130	-	-
SAMN53233839	ST23	KL1	O1/O2v2	China	2016	Liver	4	130	-	-
SAMN53233840	ST23	KL1	O1/O2v2	China	2017	Liver	4	130	-	-
SAMN53233841	ST23	KL1	O1/O2v2	China	2017	Liver	4	130	-	-
SAMN53233842	ST23	KL1	O1/O2v2	China	2017	Liver	4	119	-	-
SAMN53233843	ST23-1LV	KL1	O1/O2v2	China	2017	Liver	4	109	-	-
SAMN53233844	ST23	KL1	O1/O2v2	China	2017	Liver	4	130	-	-
SAMN53233845	ST23	KL1	O1/O2v2	China	2017	Liver	4	130	-	-
SAMN53233846	ST23	KL1	O1/O2v2	China	2017	Liver	4	130	-	-
SAMN53233847	ST23	KL1	O1/O2v2	China	2017	Liver	4	130	-	-
SAMN53233848	ST23	KL1	O1/O2v2	China	2017	Liver	4	130	-	-
SAMN53233849	ST23	KL1	O1/O2v2	China	2017	Liver	4	130	-	-
SAMN53233850	ST23	KL1	O1/O2v2	China	2017	Liver	4	130	-	-
SAMN53233851	ST23	KL1	O1/O2v2	China	2017	Liver	4	130	-	-
SAMN53233852	ST23	KL1	O1/O2v2	China	2017	Liver	4	130	-	-
SAMN53233853	ST23	KL1	O1/O2v2	China	2018	Liver	4	109	-	-
SAMN53233854	ST23	KL1	O1/O2v2	China	2018	Liver	10	130	-	-
SAMN53233855	ST23	KL1	O1/O2v2	China	2018	Liver	4	130	-	-
SAMN53233856	ST23	KL1	O1/O2v2	China	2018	Liver	4	130	-	-
SAMN53233857	ST23	KL1	O1/O2v2	China	2018	Liver	4	130	-	-
SAMN53233858	ST23	KL1	O1/O2v2	China	2018	Liver	4	130	-	-
SAMN53233859	ST23	KL1	O1/O2v2	China	2018	Liver	4	130	-	-
SAMN53233861	ST23	KL1	O1/O2v2	China	2018	Liver	4	130	-	-
SAMN53233862	ST23	KL1	O1/O2v2	China	2018	Liver	4	130	-	-
SAMN53233863	ST23	KL1	O1/O2v2	China	2018	Liver	4	130	-	-
SAMN53233864	ST23-1LV	KL1	O1/O2v2	China	2018	Liver	4	130	-	-
SAMN53233865	ST23	KL1	O1/O2v2	China	2018	Liver	4	130	-	-
SAMN53233866	ST23	KL1	O1/O2v2	China	2018	Liver	4	130	-	-
SAMN53233867	ST23	KL1	O1/O2v2	China	2018	Liver	4	129	-	-
SAMN53233868	ST23	KL1	O1/O2v2	China	2018	Liver	4	126	-	-
SAMN53233869	ST23	KL1	O1/O2v2	China	2018	Liver	4	130	-	-
SAMN53233870	ST23	KL1	O1/O2v2	China	2018	Liver	4	130	-	-
SAMN53233872	ST23	KL1	O1/O2v2	China	2018	Liver	4	130	-	-
SAMN53233873	ST23	KL1	O1/O2v2	China	2018	Liver	4	130	-	-
SAMN53233888	ST23	KL1	O1/O2v2	China	2018	Liver	4	130	-	-

SAMN53233874	ST23	KL1	O1/O2v2	China	2018	Liver	4	130	-	-
SAMN53233875	ST23	KL1	O1/O2v2	China	2018	Liver	4	130	-	-
SAMN53233876	ST23	KL1	O1/O2v2	China	2018	Liver	4	130	-	-
SAMN53233877	ST23	KL1	O1/O2v2	China	2018	Liver	4	130	-	-
SAMN53233878	ST23	KL1	O1/O2v2	China	2018	Liver	4	130	-	-
SAMN53233879	ST23	KL1	O1/O2v2	China	2018	Liver	4	130	-	-
SAMN53233880	ST23	KL1	O1/O2v2	China	2013	Liver	4	130	-	-
SAMN53233882	ST23	KL1	O1/O2v2	China	2018	Liver	4	130	-	-
SAMN53233883	ST23	KL1	O1/O2v2	China	2018	Liver	4	130	-	-
SAMN53233884	ST23	KL1	O1/O2v2	China	2013	Liver	4	130	-	-
SAMN53233885	ST23	KL1	O1/O2v2	China	2013	Liver	4	130	-	-
SAMN53233886	ST23	KL1	O1/O2v2	China	2013	Liver	4	130	-	-
SAMN53233887	ST23	KL1	O1/O2v2	China	2013	Liver	4	130	-	-

Num ARGs, Number of antibiotic resistance genes; Num VGs, Number of virulence genes; CPKP, carbapenemase-producing *Klebsiella pneumoniae.*
